# Broadcast ritual dynamics among US Catholics during the 2025 papal conclave

**DOI:** 10.1093/pnasnexus/pgag307

**Published:** 2026-09-16

**Authors:** David Willinger, Selina Volsa, Stefan Stieger

**Affiliations:** Department of Psychological Methodology, Faculty of Psychology, Karl Landsteiner University, Dr.-Karl-Dorrek-Straße 30, Krems an der Donau 3500, Austria; Department of Psychological Methodology, Faculty of Psychology, Karl Landsteiner University, Dr.-Karl-Dorrek-Straße 30, Krems an der Donau 3500, Austria; Department of Psychological Methodology, Faculty of Psychology, Karl Landsteiner University, Dr.-Karl-Dorrek-Straße 30, Krems an der Donau 3500, Austria

**Keywords:** collective emotion, social identity, religious rituals, media events, experience sampling

## Abstract

The 2025 papal conclave offered a natural experiment to trace how shared emotion and group identity evolve on the timescale of hours to days after a global broadcast ritual. Smartphone prompts delivered four surveys per day to 117 US Catholics from 2 days before the first ballot until 12 days after the inaugural blessing, yielding over 5,900 observations. Continuous-time models estimated that collective enthusiasm showed a small, instantaneous surge (0.25 SD) at the “Habemus Papam” announcement and then decayed by half within 16 hours. Religious identity changed more slowly, with a half-life of 42 h, and exhibited a comparable jump (0.20 SD) at the announcement, both jumps measured relative to the prevailing balloting-phase level. Dynamic cross-effects showed that elevated enthusiasm predicted modest gains in identity (cross-drift = 0.18 SD day^−1^), whereas higher identity predicted reduced enthusiasm (cross-drift = −0.39 SD day^−1^), suggesting regulatory feedback. Ritual phases also shifted trajectories: during secret balloting, constant inputs for enthusiasm (+0.13 SD) and identity (+0.12 SD) were modestly elevated relative to preballoting, whereas postannouncement phases slightly undershot preritual baselines. Finally, participants with above-average combined engagement showed little to no decline in self-rated religiosity, whereas those below average declined by 0.41 SD. These findings demonstrate a two-step process—an immediate wave of collective affective alignment followed by a slower, lasting identity build—and show that mediated rituals can align dispersed audiences and prolong the salience of group identity.

Significance statement Because major global events are today predominantly experienced via screens rather than through physical copresence, understanding how they shape people's connections is critical for leaders and organizers seeking to foster community. This study shows that global rites ignite a brief wave of shared enthusiasm that fades within hours while strengthening group identity for days. Recognizing this two-step process clarifies when engagement is most intense, when disengagement is most likely, and how continued participation can prolong the salience of group identity. Beyond religion, these dynamics are likely relevant to national ceremonies, memorials, and sports—any event that gathers thousands or millions at once—offering insight into how broadcast coverage can temporarily align group identification among dispersed audiences.

## Introduction

On May 8, 2025, white smoke rose above St. Peter's Square and more than 1.3 billion Catholics tuned in within minutes. As religious and civic rituals increasingly shift from physical gatherings to digital screens (1, 2), research on media rituals shows that live broadcasting aligns attention almost instantly, and mediatization studies argue that round-the-clock coverage stretches that impact well beyond the live moment (2, 3). Social identity models address that shared attention is briefly inflating a sense of “us” (4), but leave unclear when and why that state endures. A temporal puzzle remains: does collective emotion increase, fade, or stabilize in the hours and days after a global event? To our knowledge, this is the first study to trace minute to week trajectory in dispersed groups.

Empirical work has relied on minute level, session level, and multiday designs. Minute level field studies have often captured physiological or behavioral coupling: heart rates align during fire walking (5), cooperation improves after synchronized marching (6), and autonomic arousal rises throughout multihour football fan rituals (7). Session level laboratory studies show that ritual participation lifts generosity within the same visit (8) and that ordeal severity shapes donations minutes later (9). Multiday panels have been used in the context of demonstrations, festivals, and televised tournaments: perceived emotional synchrony predicts identification and empowerment over roughly 2 weeks (10, 11) and tracks national mood during football events (12–15). A five decade meta-analysis confirms that collective rites reliably boost unity (16).

Yet, important gaps remain. Minute level work stops within a day, missing how shared feeling rises and falls during the subsequent broadcast and social-media coverage that keep public attention alive, whereas multiday studies collect only two or three coarse waves, masking peaks and troughs between surveys. Investigations that follow purely broadcast audiences are rarer still: existing large-scale projects gather post event snapshots or sample citizens inside the physical crowd (15, 17). Whether a shared broadcast moment can generate both an immediate surge and a continuous bond therefore remains open, demanding data with high temporal resolution across a multiweek horizon. Resolving the temporal sequence is crucial to examine neo-Durkheimian claims (18), about whether shared emotion fosters stable social identity or is merely a brief byproduct of media events.

The 2025 papal conclave offered an unusual opportunity to fill that gap. Its fixed liturgy, nonstop coverage, and climactic “Habemus Papam” announcement aligned the attention of Catholics across time zones without physical copresence. We followed 117 US Catholics with smartphone experience sampling four prompts per day from 2 days before the first ballot through 12 days after the inaugural blessing. Each prompt recorded *perceived collective enthusiasm* (current intensity, valence, sharedness) and religious *identity salience* (momentary importance of belonging to the Catholic community). We traced the cross-lagged dynamics between the two states, and pre/postshifts of self-rated religiosity. We tested five predictions: (i) enthusiasm spikes would precede rises in identity; (ii) both states would show phase-specific baseline shifts during balloting and after the new pope's blessing; (iii) the “Habemus Papam” moment would trigger instantaneous jumps in intensity; and (iv) would also align perceived emotional states, resulting in a reduction in interindividual variance; and (v) higher average engagement across the 2 weeks would be associated with higher postconclave religiosity.

Embedding dense experience sampling within a globally followed rite therefore yields a continuous portrait of how collective emotion unfolds and how firmly it binds believers to their group in the short-term. The data show a two-step process: a brief emotional peak drives a longer-lived identity surge, and that surge is associated with a mitigated postevent cooldown in religiosity.

## Results

In our study, 117 participants completed a total of 5,903 questionnaires (Fig. 1). The average number of observations per person was *k* = 51 (SD = 20) over a mean study length of 13.5 days (SD = 2.6). To facilitate interpretation, all reported parameters and coefficients are expressed in standardized units (see Materials and methods for details on effect sizes).

**Figure 1:**
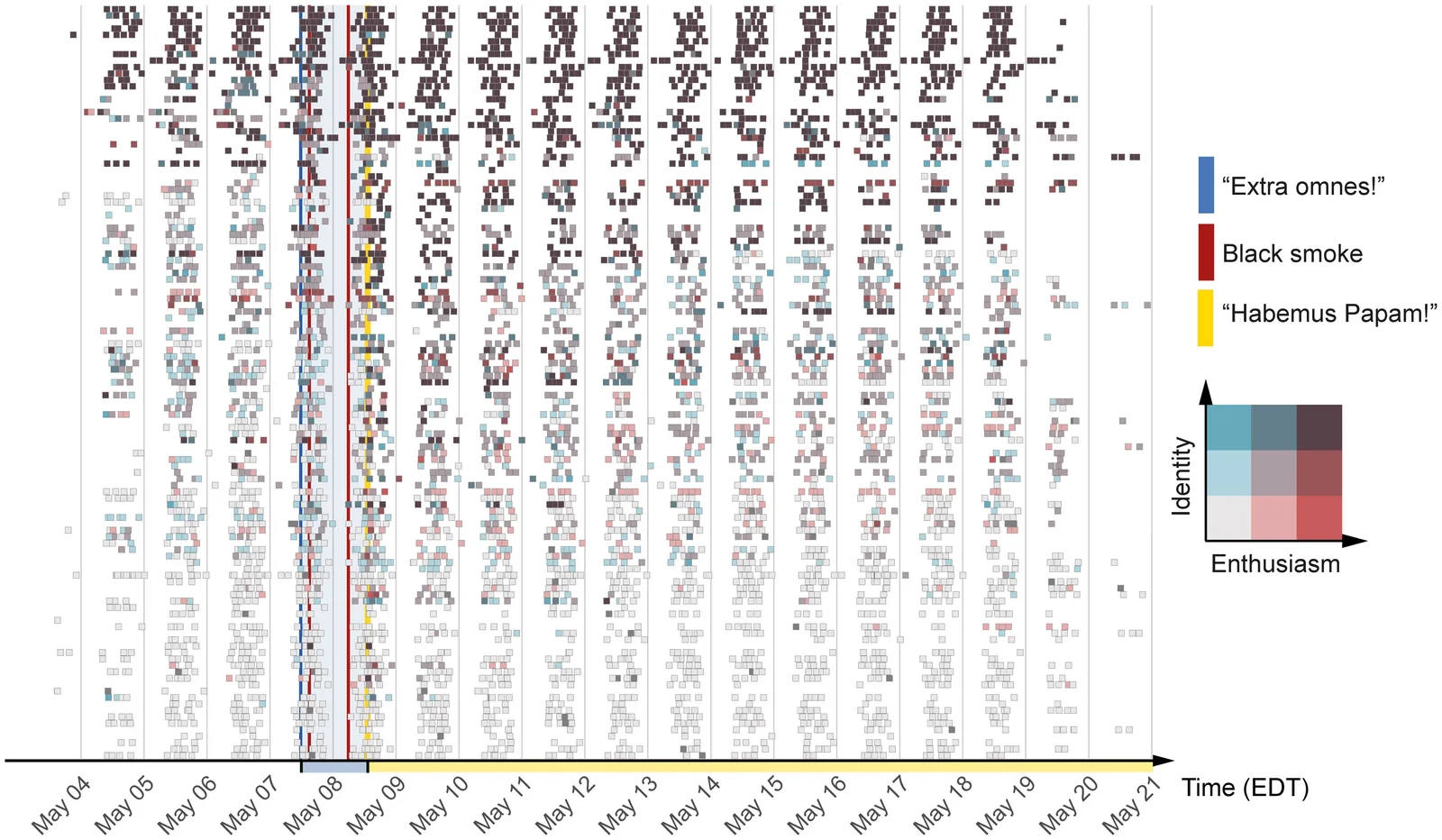
Valid responses across the 2-week study period. Each row represents one of the 117 participants, and each dot marks a valid experience-sampling response at the corresponding time (*x*-axis, Eastern Daylight Time [EDT]). Dot color encodes the simultaneous levels of collective enthusiasm and religious identity, with the bivariate key. Vertical lines highlight ritual milestones: the “Extra omnes!” call (leftmost, blue), black-smoke signals (red), and the “Habemus Papam” announcement (rightmost, yellow).

### Participants evaluated the conclave positively

Participants reacted favorably to the new pope Leo XIV and to the conclave overall. Visual-analog ratings (1–100) indicated high satisfaction with the pope (*M* = 82.7, SD = 17.9, *n* = 110), strong wonder evoked by the conclave (*M* = 71.9, SD = 22.0, *n* = 110), and considerable hope for the Church's future (*M* = 78.5, SD = 20.7, *n* = 109). Self-rated religiousness declined slightly after the conclave (*M* = 68.4, SD = 21.0, *n* = 110; preconclave: *M* = 72.6, SD = 19.1; *t*[103] = −2.83, *P* = 0.006, Cohen's *d* = −0.28, 95% CI [−0.47, −0.08]; analyses restricted to participants with both measurements). Theological orientation was slightly more liberal after the conclave (*M* = 52.5, SD = 28.9, *n* = 109; preconclave: *M* = 46.1, SD = 28.4; *t*[102] = 2.56, *P* = 0.012, Cohen's *d* = 0.25, 95% CI [0.06, 0.44]). A binary item revealed that around 18% of respondents questioned their belonging to the Catholic Church after the end of the study. At the end of the study, participants also reported higher positive affect (Positive and Negative Affect Schedule [PANAS]-PA: *M* = 38.8, SD = 7.64) than negative affect (PANAS-NA: *M* = 14.3, SD = 6.60, *n* = 117) regarding the conclave; and a high level of collective connectedness during the conclave in retrospect (*M* = 43.5, SD = 8.88, *n* = 117; possible range: 8–56). Participants also noted that sharing US nationality with the newly elected pope had an impact on their feelings toward him (scale 1–9 impact of US nationality: *M* = 7.04, SD = 1.60, *n* = 114). Bivariate correlation analyses revealed that this national identification was moderately, positively correlated with overall satisfaction with the new pope (*r* = 0.39, *t*[106] = 4.33, *P* < 0.001) and positively correlated with the pre-to-post change in self-rated religiosity (*r* = 0.23, *t*[101] = 2.37, *P* = 0.020).

### Dynamic relations between enthusiasm and identity

Continuous-time modeling of smartphone prompts across participants showed a markedly faster return-to-baseline for *perceived collective enthusiasm* than for *identity salience* (Fig. 2A; all continuous-time SEM results summarized in Table 1). *Perceived collective enthusiasm* surges halved in ≈16 h (*A*_PCE → PCE_ = −1.06 SD day^−1^, 95% CI [−1.36, −0.81]); *identity* declines were slower, halving in ≈42 h (*A*_IS → IS_ = −0.39 SD day^−1^, 95% CI [−0.59, −0.19]). Thus, brief “in-sync” waves faded overnight, whereas heightened Catholic identity persisted into subsequent days.

**Figure 2:**
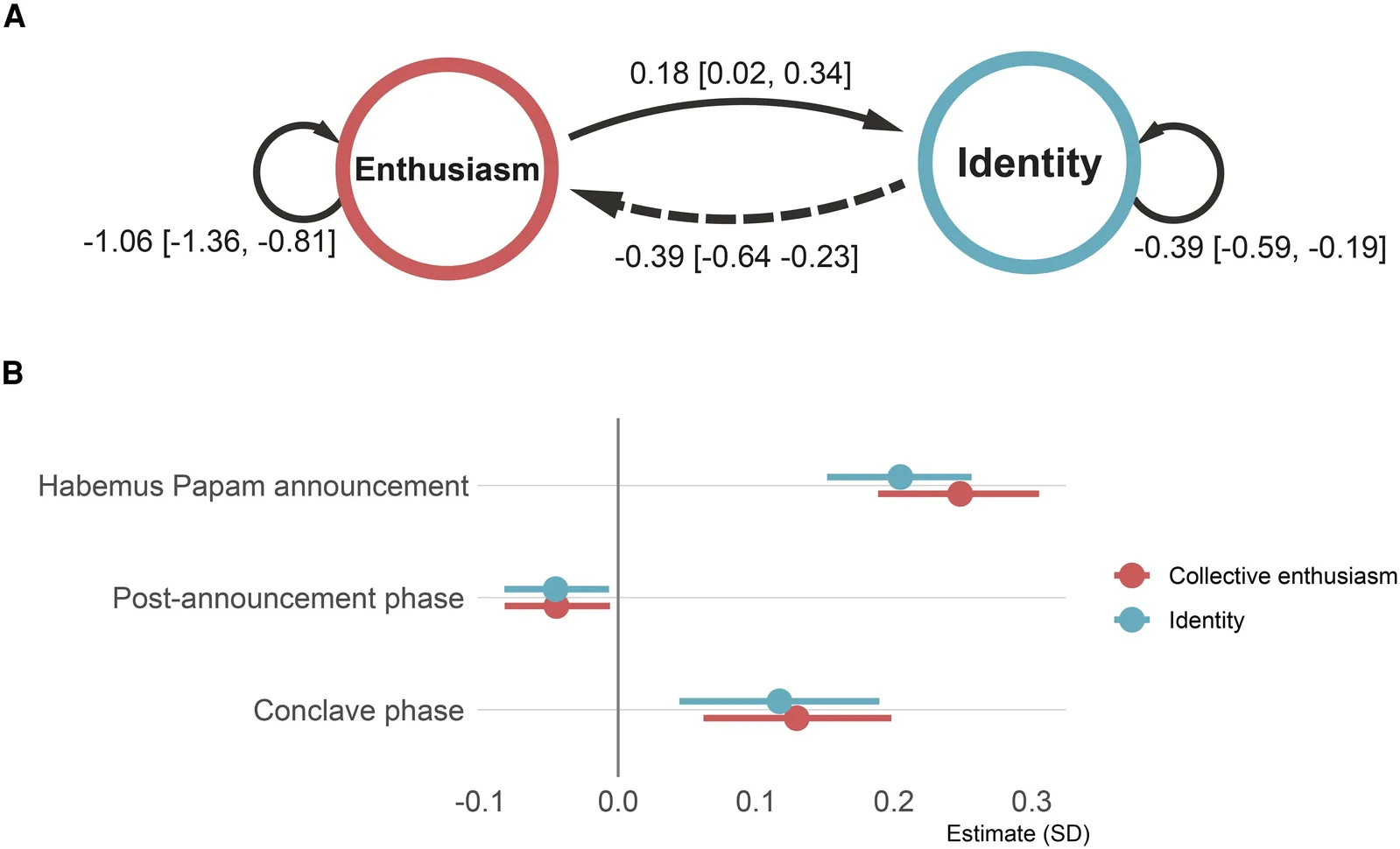
Temporal dynamics of perceived collective enthusiasm and religious identity salience. A) Schematic of the continuous-time model showing *self-regulation* and *cross-drift* effects between perceived collective enthusiasm (left, red) and religious identity (right, blue). Loop arrows display drift coefficients (negative values) that quantify the rate of return toward the set-point for each state; the solid arrow shows the directional coupling of enthusiasm and identity, and the dashed arrow shows the reverse. All estimates are in SD units, with 95% CI in brackets. B) Phase-specific constant inputs and event-related impulses. Circles mark the continuous intercepts of enthusiasm (red) and identity (blue) during the conclave and postannouncement phases; triangles mark the instantaneous impulses triggered by the Habemus Papam event. Horizontal lines indicate 95% CI around each estimate.

**Table 1:** Parameters and 95% confidence intervals for continuous-time SEM parameters tracking perceived collective enthusiasm and religious identity salience around the papal conclave.

Parameter	Mean	95% CI
Measurement model		
*λ*_Valence → PCE_	1.00	(Fixed)
*λ*_Intensity → PCE_	1.13	[1.10, 1.15]
*λ*_Sharedness → PCE_	1.10	[1.07, 1.13]
*λ*_ID1 → IS_	1.00	(Fixed)
*λ*_ID2 → IS_	1.05	[1.04, 1.08]
*λ*_ID3 → IS_	1.08	[1.06, 1.11]
Var(ε_valence_)	0.59	[0.58, 0.61]
Var(ε_intensity_)	0.42	[0.41, 0.43]
Var(ε_sharedness_)	0.46	[0.45, 0.48]
Var(ε_ID1_)	0.52	[0.51, 0.53]
Var(ε_ID2_)	0.43	[0.42, 0.44]
Var(ε_ID3_)	0.38	[0.37, 0.39]
Drift matrix (A)		
A_PCE → PCE_	−1.06	[−1.36, −0.81]
A_IS → PCE_	−0.39	[−0.64, −0.23]
A_PCE → IS_	0.18	[0.02, 0.34]
A_IS → IS_	−0.39	[−0.59, −0.19]
Diffusion covariance (GG^T^)		
Var(PCE)	0.06	[0.05, 0.07]
Cov(PCE, IS)	0.05	[0.05, 0.06]
Var(IS)	0.05	[0.05, 0.06]
Continuous intercepts (*b*)		
*b*_PCE, conclave_	0.13	[0.06, 0.20]
*b*_PCE, postannouncement_	−0.04	[−0.08, −0.01]
*b*_IS, conclave_	0.12	[0.04, 0.19]
*b*_IS, postannouncement_	−0.05	[−0.08, −0.01]
*u*_baseline_	0.01	[−0.04, 0.06]
Exogenous events		
*γ*_PCE,habemus papam_	0.25	[0.19, 0.31]
*γ*_IS,habemus papam_	0.20	[0.15, 0.26]

*N* = 117 participants, *k* = 5,903 assessments, all manifest variables were standardized prior to analysis. PCE is indicated by valence, intensity, and sharedness items; Religious identity salience (IS) by three identity items. Drift (A) parameters: negative values imply regression toward baseline, positive values imply amplification. Cross-drift effects show directional coupling between PCE and IS. Continuous intercepts (b) reflect phase shifts: “conclave” (active voting) and “postannouncement” (after Habemus Papam) relative to the baseline intercept. Exogenous events (*γ*) capture the immediate impact of the “habemus papam” announcement on each latent.

A 1-SD increase in *perceived collective enthusiasm* was associated with subsequent increases in identity at a rate of *A*_PCE → IS_ = 0.18 SD day^−1^, 95% CI [0.02, 0.34], consistent with the idea of collective emotion strengthening group identification. The reverse path was larger and negative: elevated *identity* was associated with accelerated decline of subsequent *enthusiasm* at a rate of *A*_IS → PCE_ = −0.39 SD day^−1^, 95% CI [−0.64, −0.23], suggesting diminishing “returns” once identity is on a higher level. Importantly, this negative coupling remained robust even when excluding participants with near-maximum average identity scores (person-mean >6.0; leaving *n* = 79), yielding a cross-drift of −0.43 SD day^−1^, 95% CI [−0.71, −0.18].

*Enthusiasm* and *identity* were tightly coupled, as identified by the diffusion correlation = 0.96, indicating that external events (e.g. news and social chatter) tended to push both constructs in the same direction.

### Phase- and event-linked perturbations: balloting and “Habemus Papam”

During the conclave, when the secret ballots took place, a constant input added ≈0.13 SD to *enthusiasm*'s daily trajectory and ≈0.12 SD to *identity*'s. After “Habemus Papam,” those inputs flipped sign, drawing *perceived collective enthusiasm* about 0.04 SD and *identity salience* 0.05 SD below their preconclave baselines (Fig. 2B). Thus, the ritual lifted both feelings while it unfolded and slightly overcorrected compared to the preconclave phase once complete.

The Habemus Papam announcement was associated with instantaneous jumps of 0.25 SD [0.19, 0.31] in *perceived collective enthusiasm* and 0.20 SD [0.15, 0.26] in *identity salience*.

### “Habemus Papam” associated with affective convergence

To ensure that the observed spikes in *perceived collective enthusiasm* reflected genuine group-level emotional alignment rather than independent, general positive affect, we used two mixed-effects models to assess the interindividual variation over time. We calculated each participant's absolute deviation from the momentary group mean, binned into 4 h wide time bins.

A model predicting this deviation based on the absolute temporal distance (in hours) from the “Habemus Papam” announcement revealed a significant positive effect (standardized *β* = 0.09, 95% CI [0.06, 0.12], *P* < 0.001; marginal *R*^2^ = 0.004, conditional *R*^2^ = 0.681), indicating the importance of the resolution of the conclave: interindividual variance reached its absolute minimum at the announcement.

Furthermore, a phase-based linear mixed-effects model (LMM) confirmed that this alignment was maintained throughout the anticipatory baseline and balloting phases but dissolved significantly in the postannouncement phase (standardized *β* = 0.13, 95% CI [0.03, 0.23], *P* = 0.014; marginal *R*^2^ = 0.010, conditional *R*^2^ = 0.632). Thus, the conclave not only elevated aggregate *enthusiasm* but aligned the emotional states of dispersed participants—an alignment which systematically decayed after resolution.

### Increased engagement linked to higher postconclave religiosity

To test whether experiencing stronger shared emotion/identity helped sustain religiosity, we computed an *Engagement* composite (person-mean *Collective enthusiasm* + *Religious identity*; *r* = 0.92) and fit a mixed-effects model predicting self-rated religiosity from time (pre vs. postconclave), Engagement, and their interaction. We controlled for sex, age, nationality influence, and whether participants questioned their belonging to the Church.

Overall religiosity declined modestly (standardized *β* = −0.22, 95% CI [−0.38, −0.06], *P* = 0.007). Higher Engagement predicted higher baseline religiosity (standardized *β* = 0.45, 95% CI [0.29, 0.61], *P* < 0.001) and weakened the decline (time × engagement: standardized *β* = 0.19, 95% CI [0.03, 0.35], *P* = 0.031). Although we lack baseline population data to determine whether an 18% rate of questioning one's belonging to the Church is unusually high for such an event, our models demonstrate that the participants who did question their belonging reported lower religiosity overall (standardized *β* = −0.19, 95% CI [−0.35, −0.03], *P* = 0.021), but the questioning did not moderate the interaction of Engagement (three-way interaction: *P* = 0.655). Males reported lower religiosity overall (standardized *β* = −0.28, 95% CI [−0.56, −0.01], *P* = 0.046); age and perceived nationality influence were not significant (*P* > 0.60). These findings suggest that individuals who, on average, experienced stronger shared emotion and identity during the conclave were able to maintain their anticipatory level of religious feelings. Overall, this model explained a substantial portion of the variance in religiosity (marginal *R*^2^ = 0.370, conditional *R*^2^ = 0.671).

## Discussion

This study provides a continuous portrait of how *perceived collective enthusiasm* and *religious identity* evolve after a globally broadcast religious ritual. During the 2025 conclave, dense experience sampling from 117 US Catholics was analyzed using a continuous-time model showing that enthusiasm peaks and then recedes within hours, whereas identity builds more slowly and endures over days. Enthusiasm modestly boosts identity, but elevated identity, in turn, dampens later enthusiasm. Balloting phases sustained both affect and identity, and the Habemus Papam announcement triggered immediate but short-lived spikes. Finally, greater overall engagement across 2 weeks mitigated the modest postconclave decline.

These findings extend classic media rituals theory by demonstrating that mass broadcast events not only unify attention but also structure group bonds over multiple temporal layers. Here, the continuous model tests the claims of Dayan and Katz (3) and Couldry et al. (2), that live broadcasting synchronizes audience focus, and continuous coverage elongates ritual impact: the ballots phase elevated the constant inputs for enthusiasm and identity, sustaining collective emotion for successive hours, while the postannouncement phase produced a corrective dip relative to the anticipatory baseline. This ebb-and-flow dynamic suggests that mediatized rituals generate an immediate affective peak and then guide the trajectory of group belonging beyond the live signal. By operationalizing phase-specific constant inputs and impulse effects, this work refines the mediatization thesis with specific conditions for when and how broadcast rituals support collective engagement.

Based on neo-Durkheimian models, such as interaction ritual (18), theoretical expectations dictate a short-term, acute spike in collective *effervescence* during the ritual climax, followed by a relatively rapid decay. Long-term, however, a successful ritual is theorized to recharge “emotional energy” and leave a more prolonged effect on one's group identity. By demonstrating that momentary perceived collective enthusiasm fades relatively fast while heightened Catholic identity salience persists significantly longer, our continuous-time model empirically supports this view. Furthermore, resolving the precedence provides longitudinal support for the core Durkheimian mechanism: it suggests that shared emotion acts as a catalyst for forming a stable social identity, rather than the other way around.

At the microprocess level, the divergent stability of *collective enthusiasm* and *religious identity* reflects fundamental differences in group processes. Social *identity salience*, the temporary activation of group membership, generally declines as contextual cues fade (19). The observed half-lives quantify these processes: enthusiasm dissipates within hours, whereas identity salience slowly decays over days or weeks. Moreover, the asymmetric cross-drift coupling, where enthusiasm drives identity more weakly than identity restraining enthusiasm, reveals feedback loops that govern affective and cognitive dimensions of group integration. The negative feedback from identity to enthusiasm may reflect emotional homeostasis—affective systems restoring equilibrium after peaks (20)—or cognitive satiation once belonging feels secure (21). Quantifying these dynamics establishes the timeframes in which enthusiasm and identity salience operate. These constraints may guide laboratory and field experiments designed to manipulate each process selectively.

The association between sustained engagement and maintained personal belief underscores the lasting consequences of broadcast rituals. A composite engagement index predicted individual differences in religiosity change. Whereas the overall sample exhibited a modest religiosity decline postconclave, participants with higher than average engagement experienced no significant drop, while those below average experienced a loss. This interaction highlights how deeper integration into collective affect and identity during a ritual is linked to the preservation of personal commitment against typical postevent cooldown. These results extend meta-analytic work demonstrating that collective rites boost unity (16) by showing that mediated participation can sustain individual belief in the absence of physical copresence.

These empirical insights carry broad implications for theory, method, and practice. Theoretically, they complement existing literature on rituals by demonstrating that physical copresence is not a strict requirement for the short-term maintenance of group identity. Our results show that mediatized events can align the identity salience of dispersed participants through a two-phase process of affective arousal and identity consolidation. The mechanism for this remote alignment likely differs from the physiological and embodied synchrony observed in face-to-face rituals (22, 23). In physical gatherings, shared arousal and sensory intensity drive visceral bonding. In contrast, broadcast rituals likely rely on synchronized attention and narrative immersion (2). While this cognitive alignment is sufficient to maintain identity salience over several days, it may not translate into the same levels of parochial altruism or “fused” commitment generated by the physical discomfort and shared ordeals of embodied rituals (24).

Methodologically, the combination of dense smartphone experience sampling and continuous-time structural equation modeling establishes a replicable blueprint for analyzing temporal dynamics in large-scale naturalistic settings. Practically, organizers of religious, civic, or cultural broadcasts can leverage phase-specific narrative inputs to shape collective trajectories—amplifying engagement during critical windows or diffusing intensity to prevent burnout.

Nevertheless, several limitations warrant consideration. The US-only, smartphone-access sample limits cross-national and demographic generalizability. Participants recruited via crowdsourcing platforms like Prolific are arguably more tech-savvy than the general, older than average Catholic population (25, 26). Furthermore, because they were compensated for their participation, their continued engagement was partially extrinsically motivated. Additionally, the high frequency of ESM prompts and the in-app graphical feedback may have induced measurement reactivity. Specifically, seeing their own longitudinal trajectories could have anchored subsequent responses through a self-monitoring pathway, potentially inflating the observed stability or intensity of these states.

Second, our participants followed the ritual via screens. This differs from the multisensory overload, physical proximity, and direct social contagion experienced by Catholics attending the event in person at St. Peter's Square. Remote participation relies on a “perceived” shared emotion rather than physical copresence. Third, cultural and political factors may have modulated our effects. Because the 2025 conclave resulted in the election of Pope Leo XIV, the first US-born pope, national identification and domestic media salience may have amplified attention, enthusiasm, and identity in our US sample.

Finally, the preconclave observation window spanned only 2 days; baseline measures, especially religious identity, may already have been shifted by the death of Pope Francis (24) and the associated mourning period, complicating the isolation of purely anticipatory effects. Reliance on self-report measures precluded objective validation of *synchrony*, and the absence of physiological or social-media indicators constrains inference about the underlying mechanisms. Future research should replicate these dynamics in diverse cultural settings, test the framework on negative or ambivalent mass events, and incorporate biometric sensors or digital trace data to triangulate shared emotion and identity salience.

In sum, real-time data from a global broadcast ritual reveal that collective emotion surges almost instantly, then transitions into a period of heightened group identity—validating *cor unum et anima una* (“one heart and one soul,” Acts of the Apostles 4:32) in empirical form. This two-step trajectory resolves the temporal puzzle of mediatized rituals and lays the groundwork for understanding how shared experiences maintain connection in dispersed groups over the short-to-medium term.

## Materials and methods

### Participants

The study enrolled 134 participants and excluded 17 for non-Catholic affiliation (*n* = 3), dropout before the final questionnaire (*n* = 13), or inconsistent demographic responses (*n* = 1). Independent-samples *t*-tests showed that neither baseline theological orientation, *t*(119) = 0.67, *P* = 0.505, Cohen's *d* = 0.20, 95% CI [−0.38, 0.77] (completers: *M* = 46.51, dropouts *M* = 40.92), nor political orientation, *t*(119) = 0.61, *P* = 0.544, Cohen's *d* = 0.19, 95% CI [−0.41, 0.78] (completers: *M* = 47.85, dropouts: *M* = 42.92) did predict dropout. On average, dropping-out participants completed 6.54 daily ESM prompts (SD = 8.15, range: 0–27) during the longitudinal phase before withdrawing. For pre-to-post comparison analyses, these 13 participants were excluded via listwise deletion.

The final sample comprised 117 US citizens (47.9% female; age *M* = 45.3 years, SD = 12.0, range 19–76). Relationship status was married (47.9%), partnered (17.1%), divorced (9.4%), single (23.9%), or widowed (0.9%). Self-reported religiousness at baseline averaged 72.7 (SD = 19.1, range 17–100), theological orientation averaged 46.1 (SD = 28.4, range 1–100), and political orientation averaged 47.4 (SD = 27.0, range 1–100; see Measures for scale anchors and coding) and correlated strongly with theological orientation (*r* = 0.88).

Simulation work indicates that about 2,000 person-occasions (assessments × participants) are sufficient for stable and unbiased estimation with adequate power under a wide range of conditions for bivariate continuous-time models (27). Our design far exceeds this threshold (≈5,900 observations), so we expected adequate model performance. To obtain an a priori estimation of power specific to continuous-time cross-lagged effects, we used the Shiny application by Hecht and Zitzmann (28). Even for a very small peak cross-lag (*β* = 0.01), the expected power was ≥0.99 in our design; for larger effects reported in prior meta-analytic work the power is at ceiling. Because our final model was more complex than the one used by Hecht and Zitzmann, our estimates should be regarded as approximate.

### Procedure

Participants were recruited via the crowd-working platform *Prolific* and followed three eligibility filters: US citizenship, native English proficiency, and self-identified Christianity. To limit enrollment to Roman Catholics in communion with the Pope, the study advertisement opened with a clarification notice specifying that participation required Roman Catholic affiliation and active religious practice (e.g. Mass attendance).

Data acquisition used ESMira, an open-source experience-sampling software for mobile devices, that provided study information and consent and delivered the assessment prompts via notifications (29). Participants installed the ESMira app on Android or iOS devices. Data collection began 3rd of May 2025, four days before the Conclave, and continued for 14 days.

After completing the baseline questionnaire, the field phase started. During the field phase, participants were prompted with four time-based questionnaires each day at semirandom intervals via the app between 08:00 and 20:00 local time. At 20:00 participants were asked to fill out an end-of-day questionnaire and reminded 1 h later (see Supplementary Information S1 for all items). To accommodate irregular schedules, participants could change the exact time of the end-of-day notification. Event-contingent prompts appeared after each *black-smoke* signal and immediately after the *Habemus Papam* announcement, monitored and triggered by the authors via an anonymous chat-function in ESMira. After the announcement, the prompts included a binary item that asked whether participants knew the identity of the new pope already. The items matched the content of the time-based questionnaires. On study day 14, a final survey opened at 09:00 local time for the post-study questionnaire.

Throughout participation, the app displayed real-time graphical feedback on individual and aggregate data. Aggregate feedback was restricted to general study statistics (overall gender distribution and total number of completed questionnaires). Feedback regarding the core psychological constructs (enthusiasm and identity) was limited to participants’ own personal longitudinal trajectories. An in-app frequently asked questions (FAQ) section addressed common questions and arguably supported retention. Compensation required completion of the baseline and final surveys plus at least one time-based and one end-of-day questionnaire. Payment scaled with survey count and could reach a maximum of £11.50.

Written informed consent was obtained, and the study protocol adhered to the Declaration of Helsinki, the standards of German and Austrian Psychological Societies, the American Psychological Association. Participation was voluntary and anonymous, and participants had the right to withdraw at any time. In accordance with the guidelines of the Commission on Ethics and Scientific Integrity of the Karl Landsteiner University, Krems, Austria, formal ethical review was not required. Based on the institutional criteria for ethical self-assessment, the study was exempt because it did not involve the collection of identifiable personal data, health- or disease-related data, nor did it include clinical patients or vulnerable demographic groups.

### Measures

At baseline, participants reported age, sex, relationship status, nationality, religious confession, coarse geolocation, and three single-item visual-analog ratings: religiousness (1 = *not at all religious*, 100 = *very religious*), theological orientation (visual analog scale [VAS], 1 = *conservative*, 100 = *liberal*), and political orientation (VAS, 1 = *very conservative*, 100 = *very progressive*). Participants also completed the Satisfaction With Life Scale (SWLS (30)) and the Religious Commitment Inventory (RCI-10 (31)) and a modified PVQ-21 (32) focusing on preferred values and characteristics of the next pope, however, this was not analyzed herein.

At the end of the study, they repeated these VAS ratings and completed five conclave-specific items: the 20-item PANAS (33) anchored to the feelings about the conclave (1 = *very slightly*, 5 = *extremely*); the collective effervescence questionnaire (34); the perceived influence of the pope's US nationality (−4 = more negative, 4 = more positive); satisfaction with the new pope (1 = *not at all satisfied*, 100 = *very satisfied*); a sense of wonder elicited by the conclave (1 = *strongly disagree*, 100 = *strongly agree*); and hope for the Church's future (1 = *strongly disagree*, 100 = *strongly agree*).

Lastly, participants completed the Meaning in Life Questionnaire at the end of each day (35), but is reported elsewhere (36).

All scales reported here, SWLS (α = 0.94), PANAS (α = 0.94 for both positive and negative affect subscales), RCI-10 (α = 0.91), Collective effervescence (α = 0.94), demonstrated excellent internal consistency. Pre- (*r* = 0.73, *P* < 0.001) and postconclave (*r* = 0.65, *P* < 0.001) religiousness ratings showed substantial correlation with the RCI-10 assessed at the beginning of the study.

#### Perceived collective enthusiasm

In the time-based questionnaire, we assessed *perceived collective enthusiasm* with three items capturing valence, intensity, and sharedness of participants’ momentary affect in response to the papal conclave. Items were rated on 7-point Likert-type scales (1 = *not at all*, 7 = *very much*). The items were (i) “Right now, how positive or negative are your feelings about the conclave?,” (ii) “Right now, how strong are your feelings about the conclave?,” and (iii) “Right now, how much do you feel your emotions about the conclave are shared by other Catholics?.”

Collective enthusiasm formed a strong single factor and was highly reliable. In a two-level confirmatory factor analysis (CFA; robust maximum likelihood [MLR] estimation, *lavaan*), the three items showed solid standardized loadings (*λ *_within_ = 0.66–0.70). At the between-person level, we specified a random-intercept factor by fixing the loadings to 1, resulting in standardized loadings *λ* _between_ = 0.90–0.94. The model demonstrated adequate fit (root mean square error of approximation [RMSEA] = 0.069, 90% CI [0.053, 0.085]; standardized root mean square residual [SRMR]_within_ <0.001; SRMR_between_ = 0.020; robust CFI = 0.693; note that CFI can be artificially depressed with very few degrees of freedom, *df* = 2). In a fully crossed person × item × occasion G-study, 92% of variance was between persons, yielding a relative G-coefficient of 0.933 and dependability Φ = 0.921, indicating that person-mean enthusiasm scores are both structurally coherent and highly reliable.

Convergent and discriminant validity were examined by correlating the person-level average scores of these items with established positive and negative affect measures from the PANAS and the collective effervescence questionnaire. The averaged collective enthusiasm items correlated positively and strongly with positive affect (*r* = 0.66–0.70; all *P*s < 0.001) and effervescence (*r* = 0.77–0.83, all *P*s < 0.001), and showed weak, nonsignificant negative correlations with negative affect (*r* = −0.14 to −0.11; all *P*s > 0.12). These results support the convergent and discriminant validity of the state affect scale, indicating it reflects positive emotional responses specific to the event. Furthermore, our analysis of affective convergence (see Results) supports that these individual ratings converged into a synchronized group state during the ritual's climax (“Habemus papam”).

#### Religious identity

The salience of (Catholic) religious identity was assessed with three items measuring the momentary relevance of participants’ religious identity in the context of the papal conclave, rated on the same 7-point scale. The items were (i) “Right now, how important is being Catholic to your sense of who you are?,” (ii) “Right now, how connected do you feel to other Catholics worldwide because of the conclave?,” and (iii) “Right now, how much is the conclave making you reflect about what being Catholic means to you?.”

Religious identity also formed a coherent, unidimensional construct with excellent reliability. Using the same design for the two-level CFA, the within-person factor showed strong standardized loadings (*λ *_within_ = 0.60–0.78; standardized loadings for the random-intercept factor for the between-level resulted in *λ* _between_ = 0.91–0.97). Model fit was excellent: RMSEA = 0.014; SRMR_within_ <0.001; SRMR_between_ = 0.008; robust CFI = 0.995. The G-study attributed 95% of variance to between-person differences, yielding a relative G-coefficient of 0.950 and dependability Φ of 0.949, confirming that momentary identity scores are both unidimensional and highly reliable.

To establish convergent validity, averaged scores of the religious identity items were correlated with the RCI-10. The state identity items correlated moderately and significantly with RCI-10 total scores (*r* = 0.46–0.53, all *P*s < 0.001). Discriminant validity was evaluated by comparing state identity scores with the SWLS, showing small, nonsignificant correlations (*r* = 0.14–0.17, all *P*s > 0.05).

### Statistical analysis

All statistical analyses were performed in *R* version 4.4.3 using the *ctsem* package for continuous-time modeling (37), *gtheory* package for generalizability theory (38), *lavaan* for confirmatory factor analyses (39), and the *psych* package for reliability estimation (40).

#### Data quality and missingness

Overall compliance was 90% (SD = 18%), yielding an average of 51 observations per person (SD = 20) over 13.5 days (SD = 2.6). A mixed-effects logistic model of missingness on prompt occasion (OR = 1.033 per prompt, 95% CI [1.027, 1.040], *P* < 0.001) indicates a statistically significant but negligible increase in missing probability from 0.2% at the first prompt to 0.8% at prompt 50, supporting the use of full-information maximum likelihood (FIML) under MAR assumptions.

#### Multilevel confirmatory factor analyses for state measures

We used multilevel CFA to verify that each construct (state collective enthusiasm and religious identity) formed a unidimensional factor at both the within-person and between-person levels. At the within-person level, each latent construct was indicated by its three momentary items with factor loadings and residual variances freely estimated. At the between-person level, we specified a random-intercept factor by fixing all between-level loadings to 1, so the between-person latent variable represents a common person-level elevation across items; between-level residual variances and the latent variance were freely estimated. No cross-level equality constraints were imposed. Models were fit using MLR; missing data were handled with FIML. Because models were estimated with MLR, we report Yuan–Bentler-scaled indices; with very small degrees of freedom and a large robustness correction applied to the baseline model, robust comparative fit index (CFI)/Tucker-Lewis index (TLI) can appear artificially depressed, so we interpret them cautiously and place greater weight on robust RMSEA (90% CI) and the level-specific SRMR_within/SRMR_between. We evaluated the fit via RMSEA <0.06, and SRMR <0.08 at both levels (41, 42).

#### Reliability of state measures

To assess reliability of our composite scores across prompts, we applied Generalizability theory (38) using a *persons*  *×*  *items*  *×*  *occasions* design and estimated their variance components and the variance components of their interactions in a G-study. These components informed the decision studies (D-studies) to compute the generalizability coefficient (G) and the dependability coefficient (Φ) for each composite for our actual sample (three items × all prompts). We report G and Φ, interpreting values ≥0.80 as acceptable reliability for intensive longitudinal measurement.

#### Continuous-time structural equation model

A bivariate *continuous-time* model in *ctsem* tracked how *collective enthusiasm* and *religious identity* changed from prompt to prompt (37) to investigate their temporal dynamics that remain invisible to conventional linear mixed-model approaches (43). The core differential equation of *ctsem* is typically noted as:


$$\begin{array}{c} d\eta (t)=[A\eta (t)+b(t)+\Gamma \chi (t)]dt+GdW(t) \end{array},$$

where η(t) is a vector of latent processes (enthusiasm and identity), *A* represents the drift matrix (how latent processes influence each other over time), *b* denotes the continuous-time intercept (constant “force” or input acting on the process), Γχ denotes time-dependent covariates (the Habemus Papam event), *G* is the diffusion matrix, and d*W*(*t*) represents Gaussian white noise, with the latter two comprising the “system noise” *GdW*(*t*). In our model, the continuous-time intercept itself is phase-dependent, by including the dummy variables for *conclave is active* and *postannouncement*, so that the constant input acting on each latent process differs during these periods.


$$\begin{array}{rl} b(t)\;= & u_{\mathrm{baseline}}+\;b_{\mathrm{conclave}}ConclaveActive(t)\\ & +b_{\mathrm{post}}PostAnnouncement(t). \end{array}$$

A measurement model links the latent factors to the observed variables:


$$\begin{array}{c} y(t)=\Lambda \eta (t)+\tau +\varepsilon (t),\;\;\varepsilon (t)\;∼\;N(0_{c},\;\Theta )\; \end{array},$$

where *y*(*t*) is the vector of observed variables at each prompt, Λ contains factor loadings linking latents to observed, *τ* represents measurement intercepts, and ε(t) is the measurement error with covariance matrix Θ.

Each construct was treated as a latent process observed through three manifest, momentary items assessed via the time-based questionnaire. Manifest means were fixed to zero, measurement errors were estimated freely, and initial latent means and variances were left unrestricted. We specified random intercepts for each latent process, random individual differences in the global continuous intercept, and random “Habemus papam”-event impulse slopes. All parameters were obtained with full-information maximum likelihood.

All manifest variables were standardized (*z*-scored) prior to analysis. Because person-mean collective enthusiasm and religious identity were almost interchangeable (*r *= 0.92), we also fitted a unidimensional (1-state) model with all six items loading on one latent process. The 2-state model nevertheless outperformed the 1-state solution by a wide margin (Δ Akaike information criterion [AIC] = 2,654; likelihood ratio test *P* < 0.001), indicating that even though the two constructs overlap at the between-person level their *within-person* temporal dynamics (decay rates, phase-specific inputs, and cross-couplings) are meaningfully distinct and cannot be captured by a single latent trajectory. To further assess the robustness of these dynamics and to ensure specifically that the negative cross-coupling was not an artifact of ceiling effects, we conducted a sensitivity analysis by re-estimating the bivariate model excluding participants with near-maximum person-level identity scores (mean scores >6.0).

Absolute model fit was evaluated by comparing the empirical lagged covariance structure with the model-implied covariance structure of the continuous-time model. This is conceptually similar to the SRMR used in traditional structural equation modeling. Specifically, we generated 500 datasets from the fitted model parameters and compared the empirical covariances across lags of 0 to 10 with the 95% simulation intervals.

Model assessment revealed excellent fit: across all variables and lags, 96.0% of the empirical lagged covariances fell within the model-implied 95% simulation intervals. Furthermore, residual variances indicated that the latent factors explained roughly two-thirds of item variance.

#### Mixed-effects modeling of affective convergence

To determine whether the observed spikes in perceived collective enthusiasm reflected genuine emotional convergence rather than general increase in positive affect, we analyzed the interindividual variance over time. Data were grouped into 4-h bins to account for the semirandom sampling intervals. Within each bin, we calculated the group mean for *perceived collective enthusiasm*. The outcome variable was defined as the absolute deviation of each participant's momentary score from that bin's group mean, where lower values indicate greater convergence with the group.

We fitted two LMMs: the first model examined phase-based differences, predicting absolute deviation from the ritual phase (Baseline, Conclave, and PostAnnouncement) with random intercepts and slopes for participants. The second model tested the temporal decay of alignment by predicting absolute deviation from the absolute temporal distance (in hours) from the “Habemus Papam” announcement. Significance was assessed using Satterthwaite's degrees of freedom. Standardized effect sizes and 95% CI are reported.

#### Mixed-effects modeling longitudinal change of religiousness

To examine whether stronger average engagement during the conclave was associated with changes in self-reported religiousness, we created a person-level *Engagement* index by first standardizing each participant's mean collective enthusiasm and mean identity (*z*-scores) and then averaging them (*α* = 0.96). The outcome variable was self-rated religiousness, which was measured twice per person around 2–3 days before the conclave and 11–12 days afterward. We fitted an LMM predicting religiousness from fixed effects of time (pre vs. post), *Engagement*, their interaction, and covariates (sex, age, perceived influence of the pope's nationality), with a random intercept for each participant. This interaction term directly tests the hypothesis that individuals higher in average *Engagement* experience a higher feeling of religiosity after the conclave. Additionally, we included a binary covariate capturing whether participants reported that the experience of the conclave led them to question their belonging to the Catholic Church (1 = *yes* [*n* = 21], 0 = *no*). As above, significance was assessed via *t*-tests with Satterthwaite's approximation, and standardized effect sizes for main and interaction effects were computed.

#### Effect sizes and parameter standardization

All continuous manifest variables in the continuous-time models were standardized prior to analysis. Consequently, the drift parameters (*A*), continuous-time intercept parameters (*b*), and exogenous event jumps (*γ*) are expressed in standardized units. Discrete event jumps represent standardized step changes (conceptually analogous to Cohen's *d*), while drift parameters represent standardized rates of change in SD units. Standardized parameters for the LMMs were computed using the *R* package *effectsize* (44). Accordingly, all reported regression coefficients represent standardized effect sizes (i.e. continuous associations are expressed as standardized slopes, whereas categorical contrasts are expressed as standardized mean differences, again conceptually similar to Cohen's *d*).

## Supplementary Material

pgag307_Supplementary_Data

## Data Availability

The data and code for the analysis underlying this article are available at https://osf.io/gkvwe/.
