# Supplementary material for: Broadcast ritual dynamics among US Catholics during the 2025 papal conclave
Source: PNAS Nexus. 2026 Sep 16;5(10):pgag307. doi: 10.1093/pnasnexus/pgag307 (PMC13627957; doi:10.1093/pnasnexus/pgag307)
Supplement: Supplementary file 1 [file pgag307_supplementary_data.pdf]

07:26

Conclave: the pope's election

psychologie.forschung@kl.ac.at

Welcome to the study "**Conclave: the pope's election**". This study is conducted by a team of researchers at the Karl Landsteiner University of Health Sciences in Austria (David Willinger, Stefan Stieger, Selina Volsa). Using questionnaires presented with a scientific smartphone app (ESMira) we investigate people's emotions longitudinally before, during, and after the pope's election – the conclave.

To participate, you only have to fill in a questionnaire at the begin of the study (50 items) once, at the end of the study (30 items) once, a daily questionnaire with **6 short questions** 4 times a day, and an end-of-day questionnaire (10 items) for 14 days via the survey software ESMira. You can view and follow your own progress over time via personalized graphs.

The study is completely anonymous. No direct personal questions will be asked (name, address, etc.), nor can any connection be made between your data and your person at any time. For more details, please see the consent form and FAQs.

The key data of the study:

- The study runs over a period of 2 weeks.
- Individualized graphics (based on your data) in ESMira
- An initial survey with general questions (50 items) - 1.00 £
- An end-of-day questionnaire every evening (10 items) - each 0.13 £
- A daily questionnaire administered four times daily (6 items) - each 0.13 £
- A final questionnaire after 2 weeks (30 items) - 1.00 £

You will receive up to 11 £ as a compensation depending on the number of completed questionnaires.

Contact address:
Division of Psychological Methodology
Karl Landsteiner University of Health Sciences
Dr.-Karl-Dorrek-Strasse 30
3500 Krems an der Donau / Austria
I: http://www.kl.ac.at/
E: psychologie.forschung@kl.ac.at

‹ Zurück | Einwilligung ›

07:26

1. Einverständniserklärung

Um fortfahren zu können, lesen Sie sich bitte die Einverständniserklärung durch und stimmen Sie dieser zu.

Zeige Einverständniserklärung

2. Benachrichtigungen

Wofür?

‹ Zurück | Teilnehmen ✓

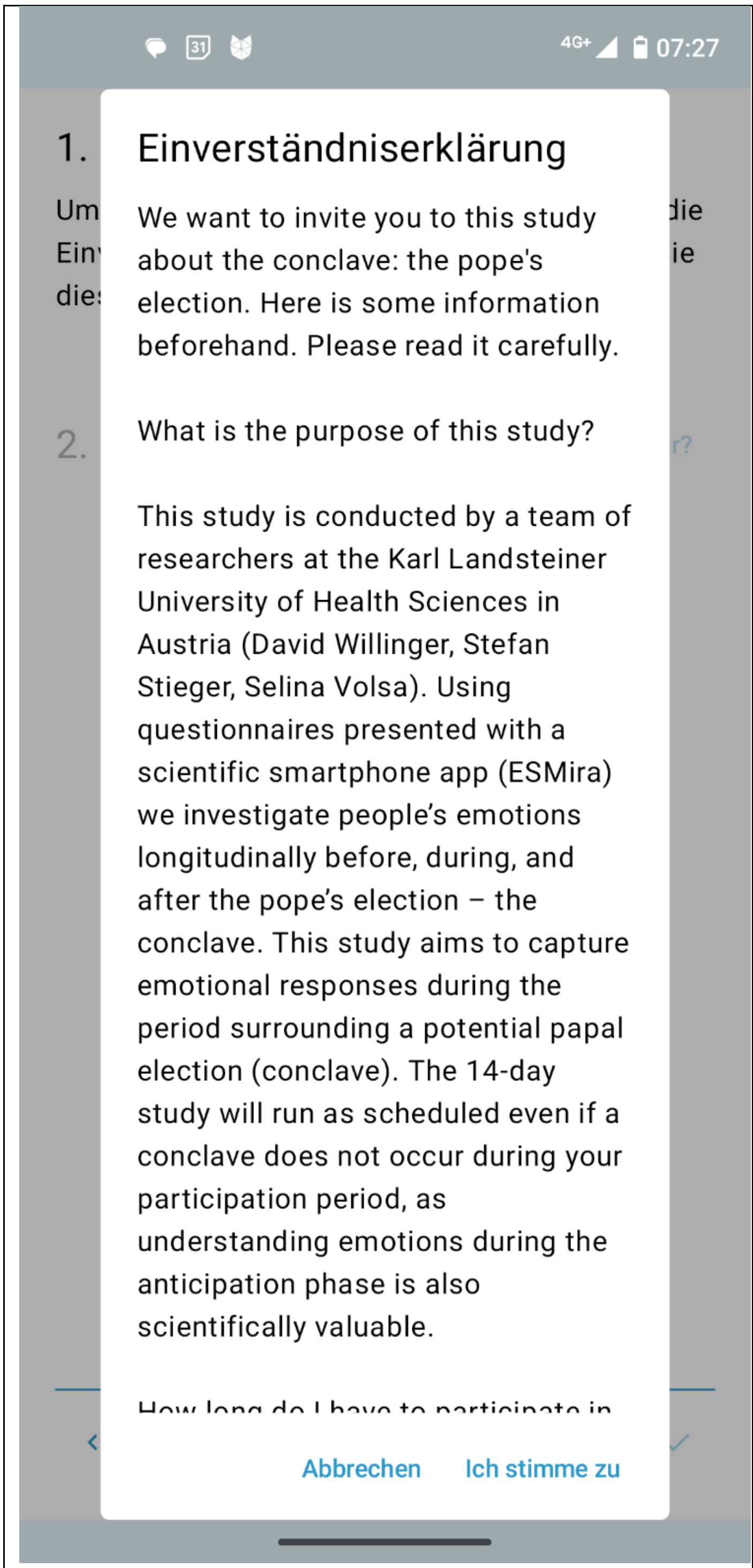
07:27
Einverständniserklärung
We want to invite you to this study about the conclave: the pope's election. Here is some information beforehand. Please read it carefully.
What is the purpose of this study?
This study is conducted by a team of researchers at the Karl Landsteiner University of Health Sciences in Austria (David Willinger, Stefan Stieger, Selina Volsa). Using questionnaires presented with a scientific smartphone app (ESMira) we investigate people's emotions longitudinally before, during, and after the pope's election – the conclave. This study aims to capture emotional responses during the period surrounding a potential papal election (conclave). The 14-day study will run as scheduled even if a conclave does not occur during your participation period, as understanding emotions during the anticipation phase is also scientifically valuable.
How long do I have to participate in
Abbrechen
Ich stimme zu

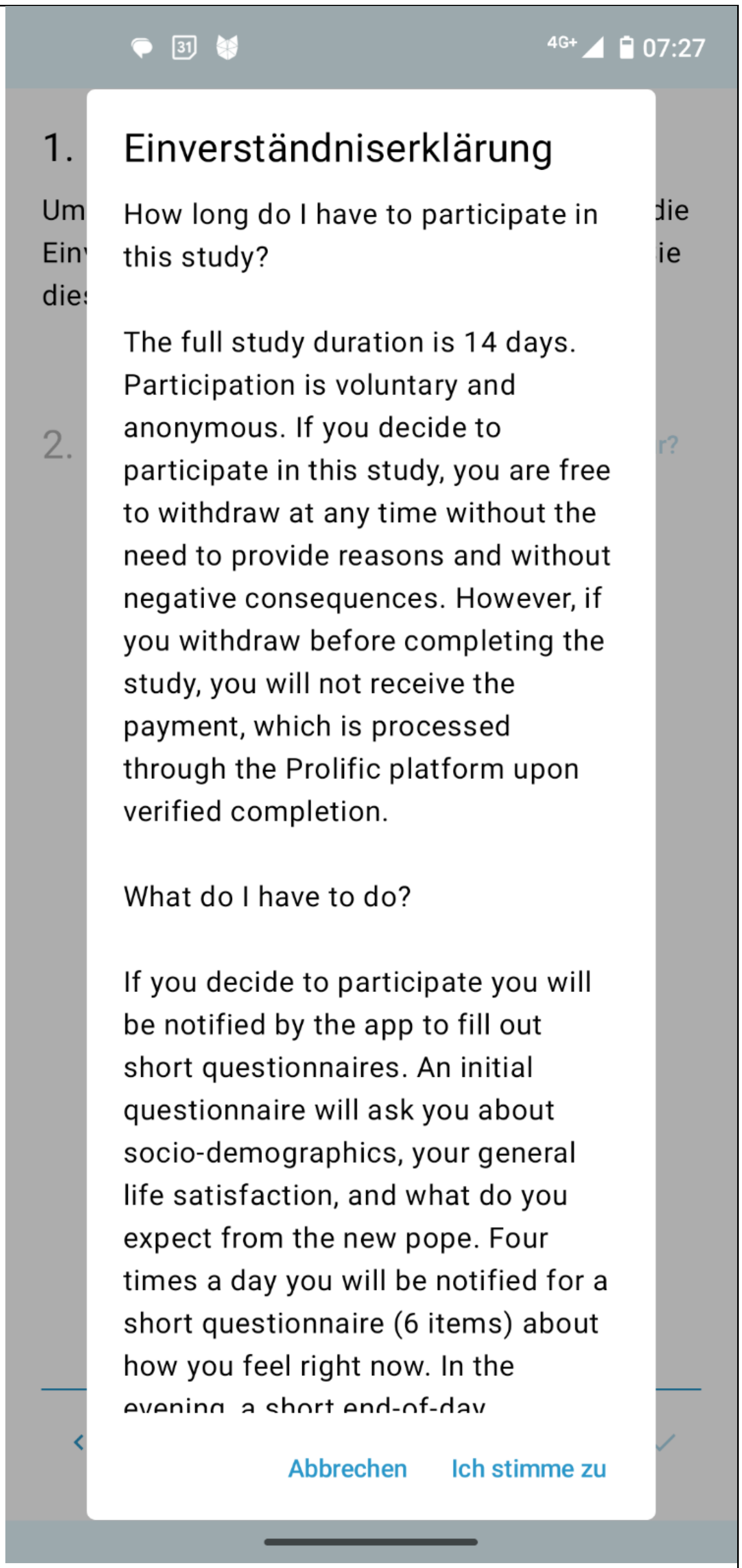
07:27
Einverständniserklärung
How long do I have to participate in this study?
The full study duration is 14 days. Participation is voluntary and anonymous. If you decide to participate in this study, you are free to withdraw at any time without the need to provide reasons and without negative consequences. However, if you withdraw before completing the study, you will not receive the payment, which is processed through the Prolific platform upon verified completion.
What do I have to do?
If you decide to participate you will be notified by the app to fill out short questionnaires. An initial questionnaire will ask you about socio-demographics, your general life satisfaction, and what do you expect from the new pope. Four times a day you will be notified for a short questionnaire (6 items) about how you feel right now. In the evening, a short end-of-day
Abbrechen
Ich stimme zu

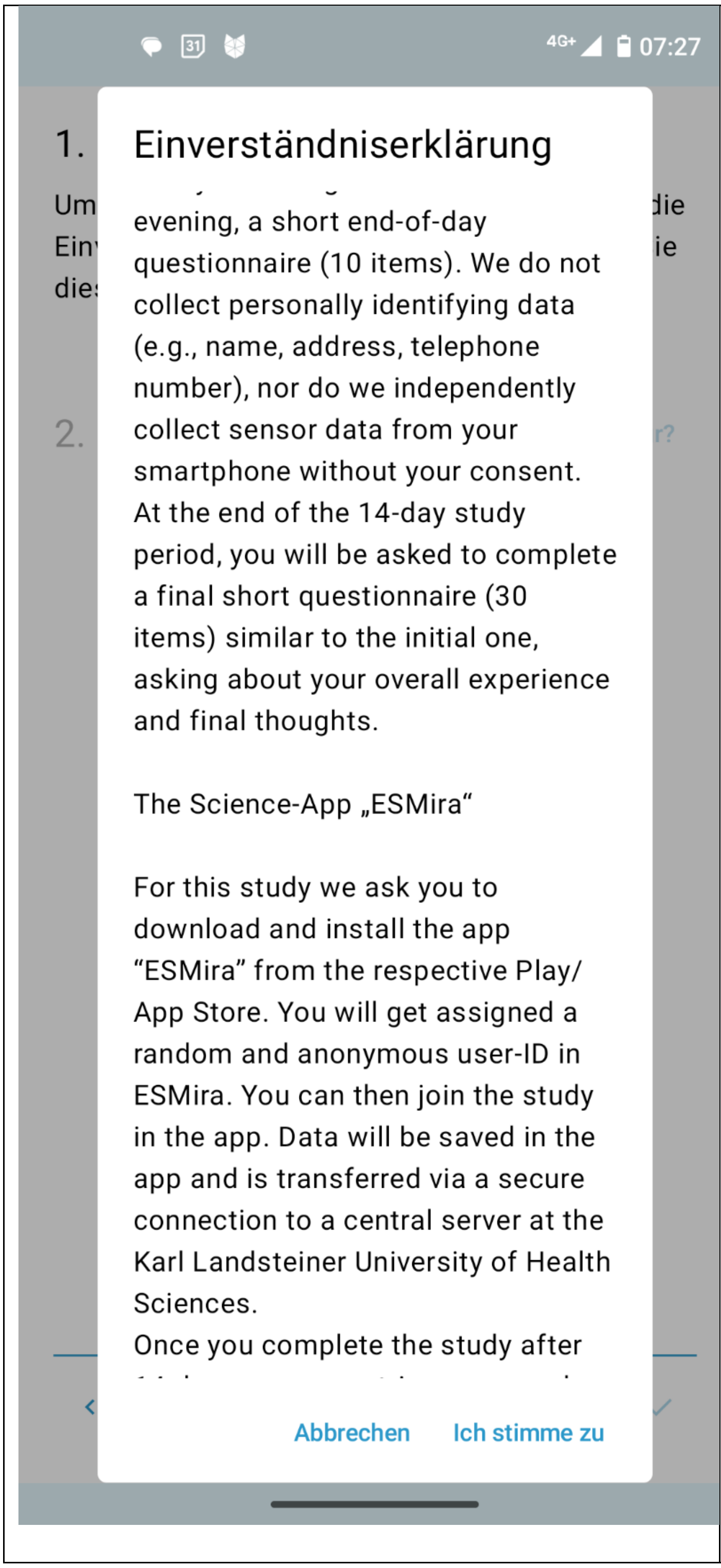

Einverständniserklärung

evening, a short end-of-day questionnaire (10 items). We do not collect personally identifying data (e.g., name, address, telephone number), nor do we independently collect sensor data from your smartphone without your consent. At the end of the 14-day study period, you will be asked to complete a final short questionnaire (30 items) similar to the initial one, asking about your overall experience and final thoughts.

The Science-App „ESMira“

For this study we ask you to download and install the app “ESMira” from the respective Play/ App Store. You will get assigned a random and anonymous user-ID in ESMira. You can then join the study in the app. Data will be saved in the app and is transferred via a secure connection to a central server at the Karl Landsteiner University of Health Sciences.
Once you complete the study after

Abbrechen Ich stimme zu

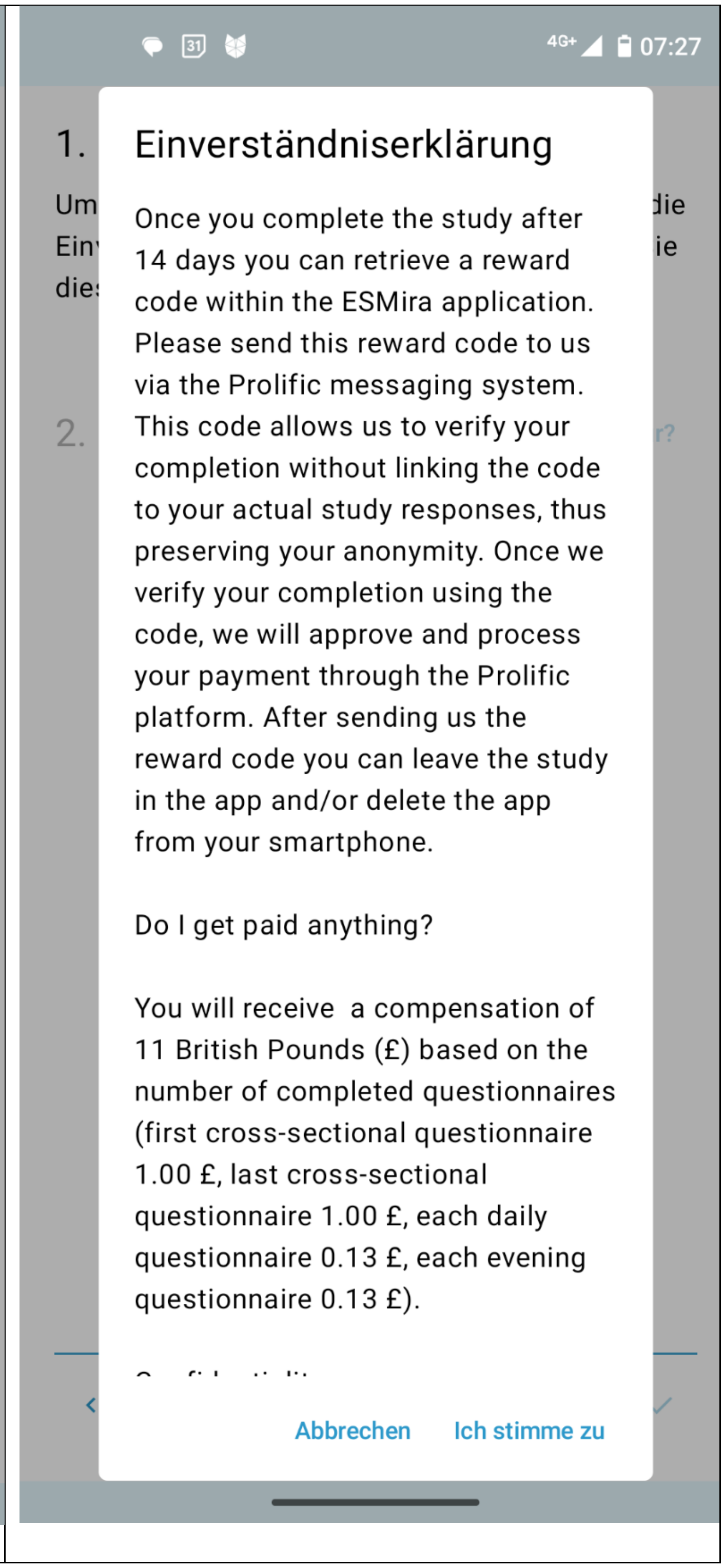

Einverständniserklärung

Once you complete the study after 14 days you can retrieve a reward code within the ESMira application. Please send this reward code to us via the Prolific messaging system. This code allows us to verify your completion without linking the code to your actual study responses, thus preserving your anonymity. Once we verify your completion using the code, we will approve and process your payment through the Prolific platform. After sending us the reward code you can leave the study in the app and/or delete the app from your smartphone.

Do I get paid anything?

You will receive a compensation of 11 British Pounds (£) based on the number of completed questionnaires (first cross-sectional questionnaire 1.00 £, last cross-sectional questionnaire 1.00 £, each daily questionnaire 0.13 £, each evening questionnaire 0.13 £).

Abbrechen Ich stimme zu

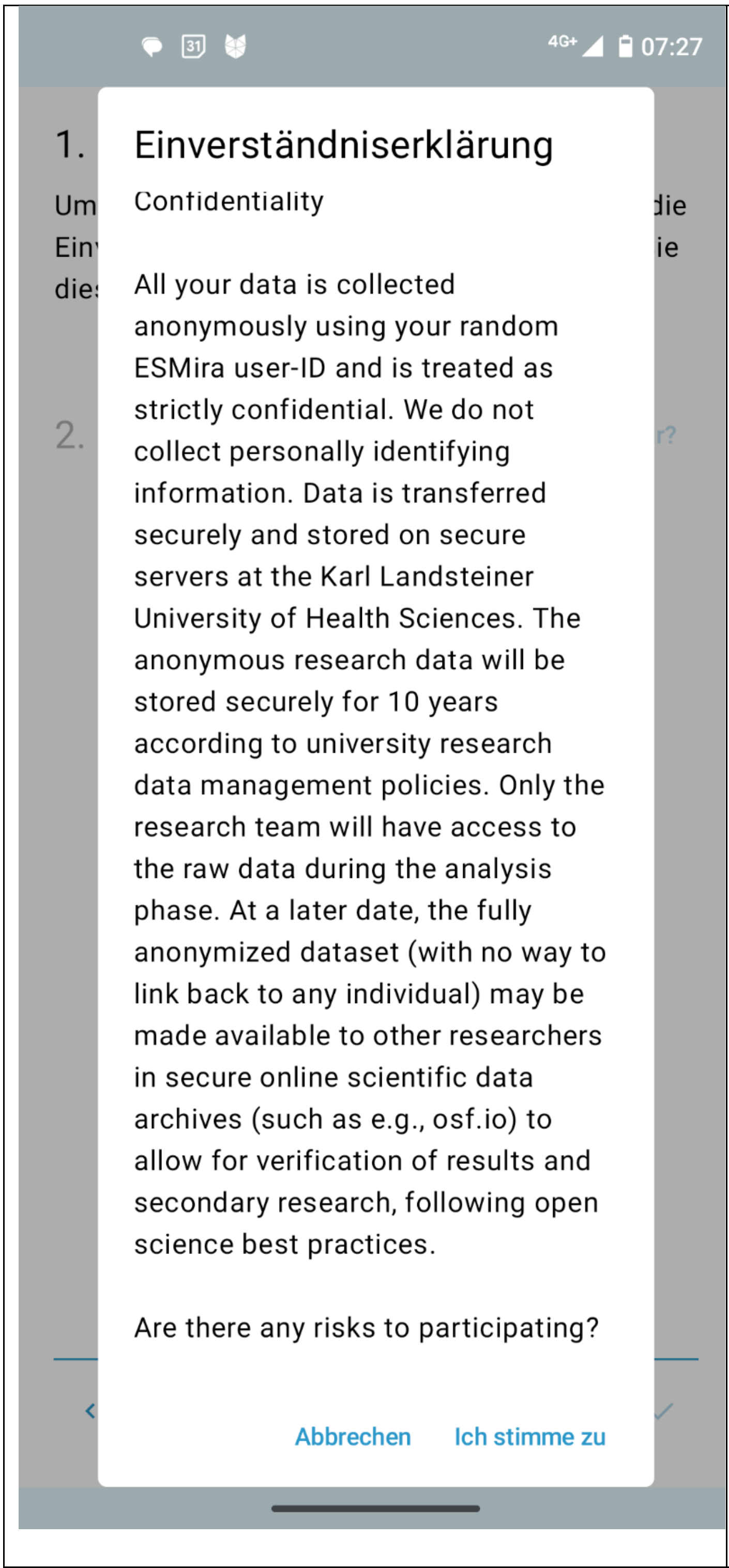

Einverständniserklärung

Confidentiality

All your data is collected anonymously using your random ESMira user-ID and is treated as strictly confidential. We do not collect personally identifying information. Data is transferred securely and stored on secure servers at the Karl Landsteiner University of Health Sciences. The anonymous research data will be stored securely for 10 years according to university research data management policies. Only the research team will have access to the raw data during the analysis phase. At a later date, the fully anonymized dataset (with no way to link back to any individual) may be made available to other researchers in secure online scientific data archives (such as e.g., osf.io) to allow for verification of results and secondary research, following open science best practices.

Are there any risks to participating?

Abbrechen Ich stimme zu

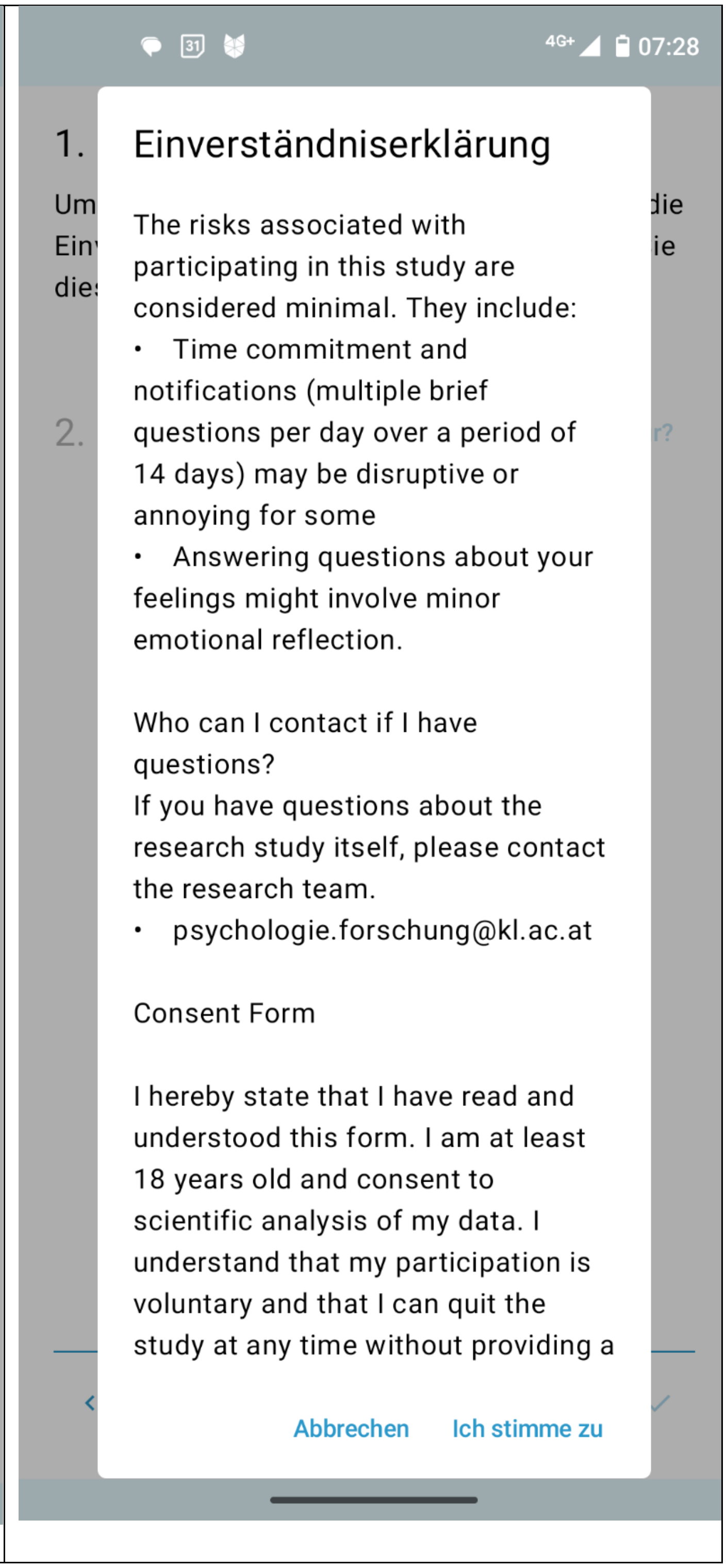

Einverständniserklärung

The risks associated with participating in this study are considered minimal. They include:

- Time commitment and notifications (multiple brief questions per day over a period of 14 days) may be disruptive or annoying for some
- Answering questions about your feelings might involve minor emotional reflection.

Who can I contact if I have questions?
If you have questions about the research study itself, please contact the research team.

- psychologie.forschung@kl.ac.at

Consent Form

I hereby state that I have read and understood this form. I am at least 18 years old and consent to scientific analysis of my data. I understand that my participation is voluntary and that I can quit the study at any time without providing a

Abbrechen Ich stimme zu

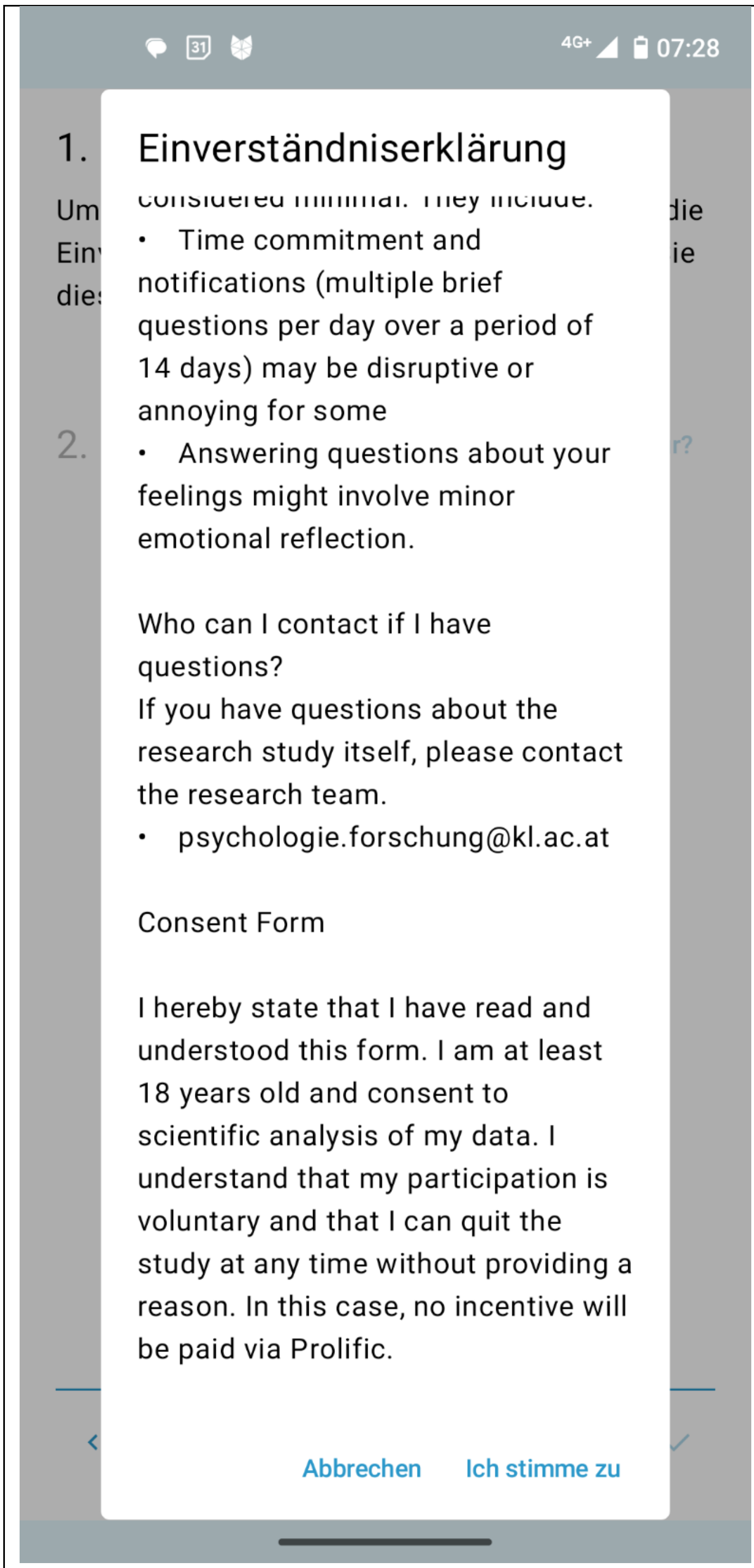
07:28
1. Einverständniserklärung
considered minimal. They include:
• Time commitment and notifications (multiple brief questions per day over a period of 14 days) may be disruptive or annoying for some
• Answering questions about your feelings might involve minor emotional reflection.
Who can I contact if I have questions?
If you have questions about the research study itself, please contact the research team.
• psychologie.forschung@kl.ac.at
Consent Form
I hereby state that I have read and understood this form. I am at least 18 years old and consent to scientific analysis of my data. I understand that my participation is voluntary and that I can quit the study at any time without providing a reason. In this case, no incentive will be paid via Prolific.
Abbrechen
Ich stimme zu

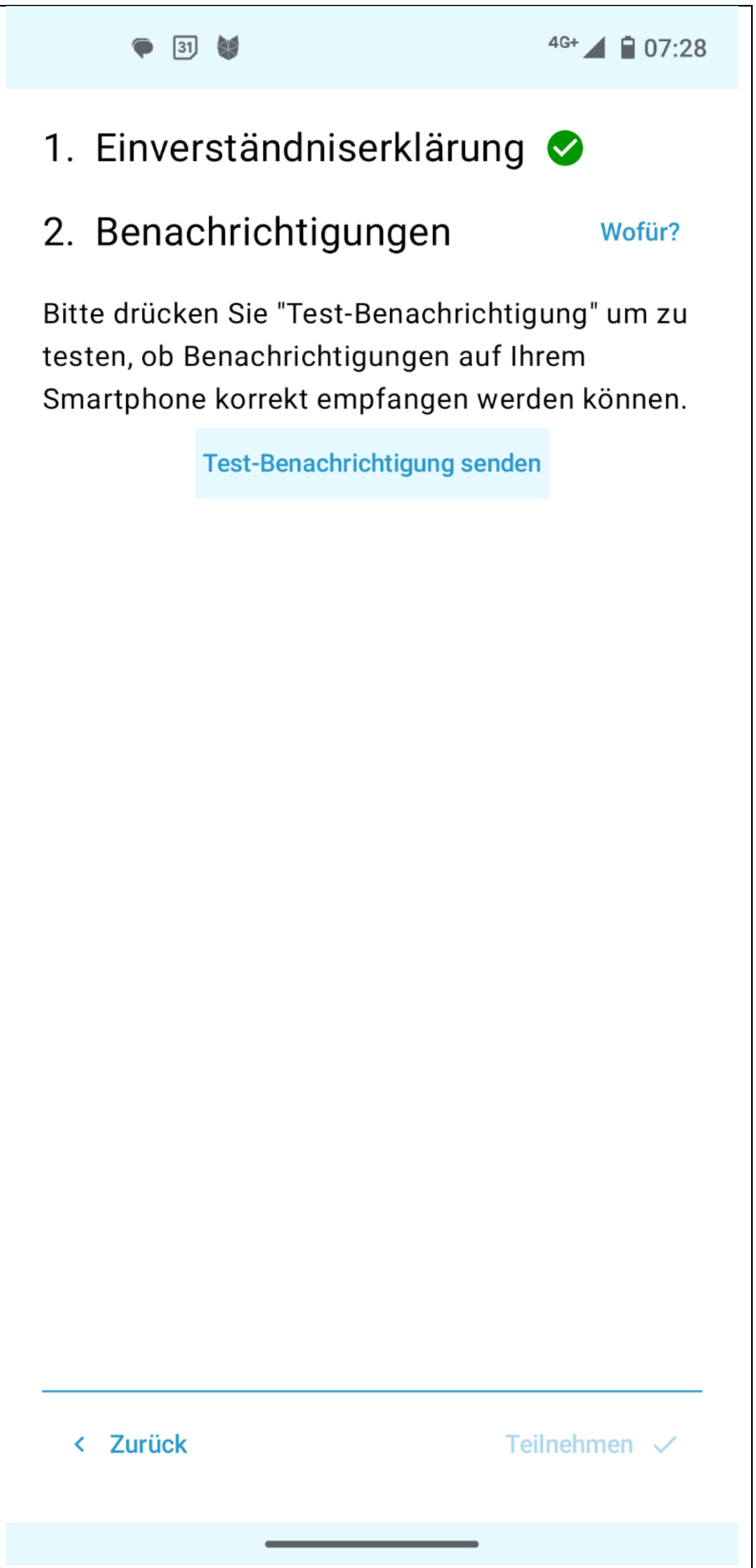
07:28
1. Einverständniserklärung
2. Benachrichtigungen
Wofür?
Bitte drücken Sie "Test-Benachrichtigung" um zu testen, ob Benachrichtigungen auf Ihrem Smartphone korrekt empfangen werden können.
Test-Benachrichtigung senden
Zurück
Teilnehmen

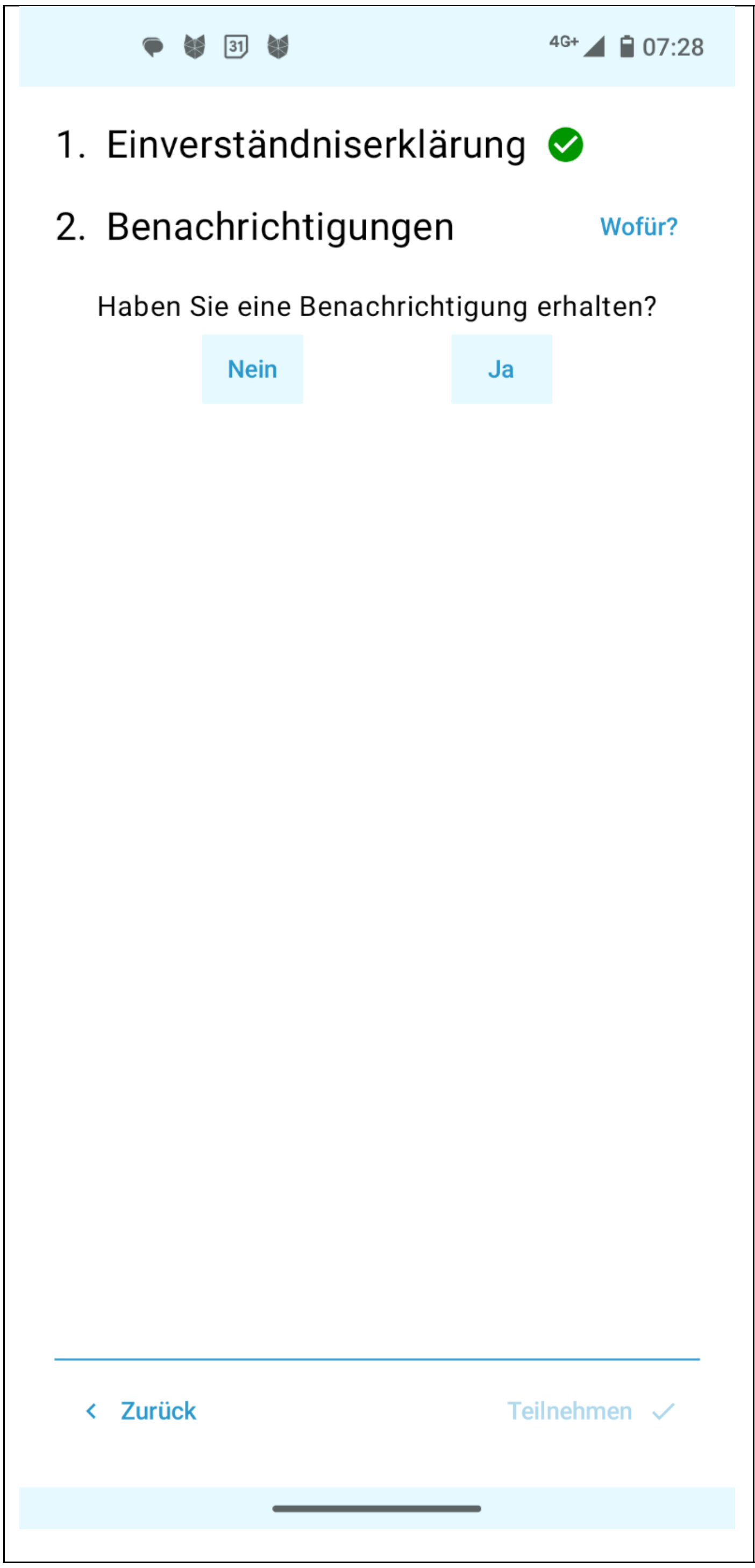
4G+ 07:28
1. Einverständniserklärung
2. Benachrichtigungen
Wofür?
Haben Sie eine Benachrichtigung erhalten?
Nein
Ja
Zurück
Teilnehmen

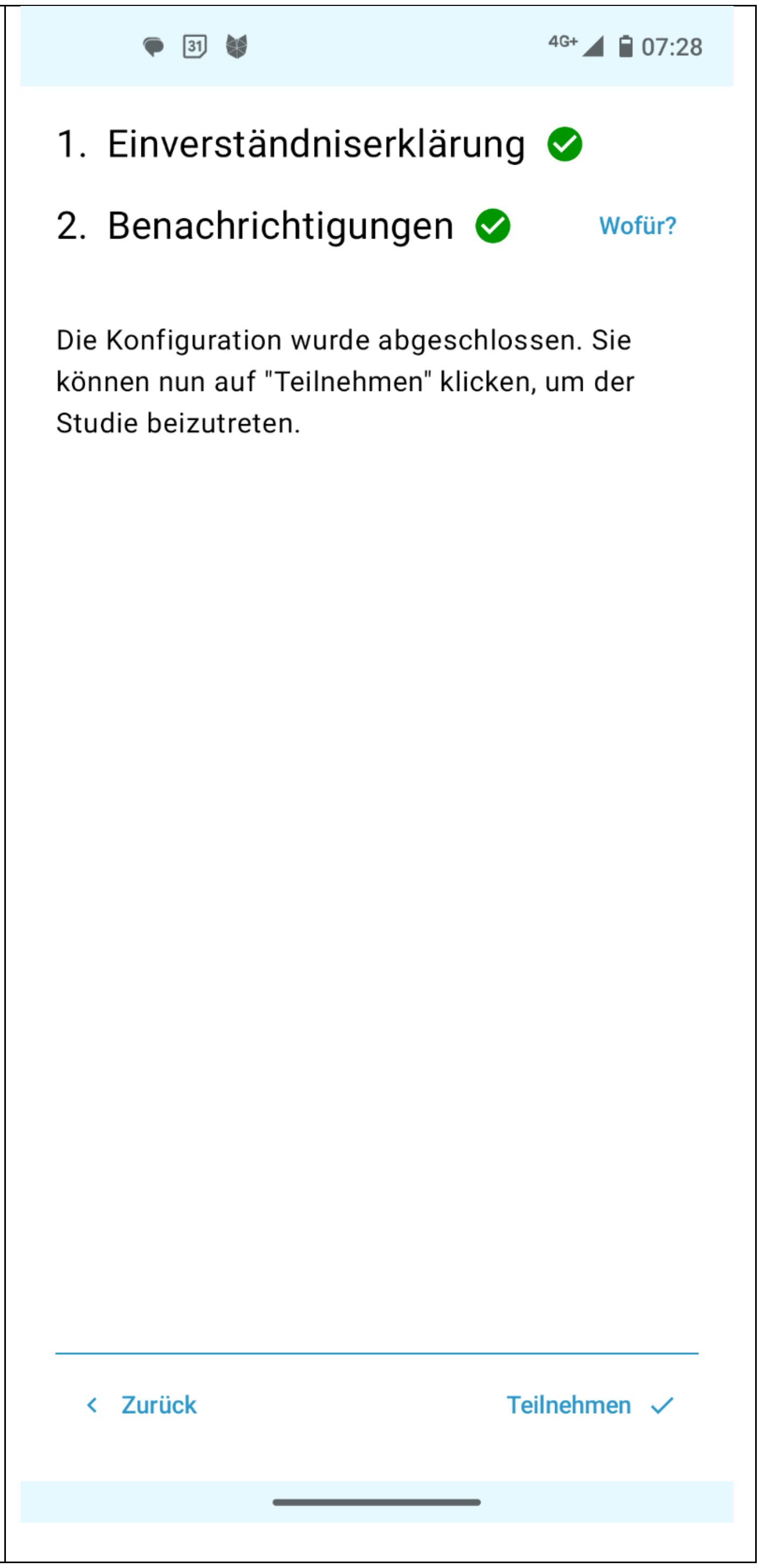
4G+ 07:28
1. Einverständniserklärung
2. Benachrichtigungen
Wofür?
Die Konfiguration wurde abgeschlossen. Sie können nun auf "Teilnehmen" klicken, um der Studie beizutreten.
Zurück
Teilnehmen

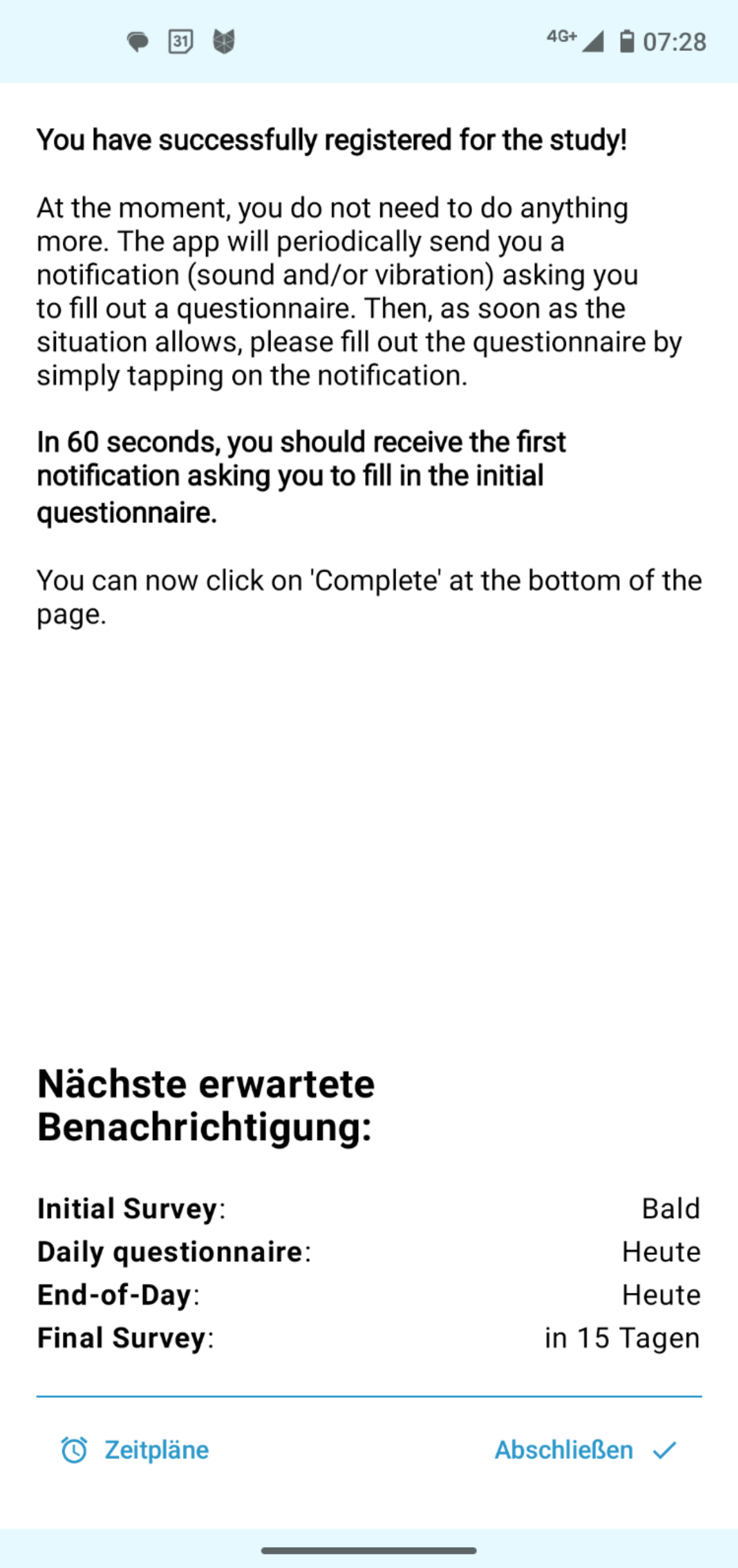
07:28
You have successfully registered for the study!
At the moment, you do not need to do anything more. The app will periodically send you a notification (sound and/or vibration) asking you to fill out a questionnaire. Then, as soon as the situation allows, please fill out the questionnaire by simply tapping on the notification.
In 60 seconds, you should receive the first notification asking you to fill in the initial questionnaire.
You can now click on 'Complete' at the bottom of the page.
Nächste erwartete Benachrichtigung:
Initial Survey: Bald
Daily questionnaire: Heute
End-of-Day: Heute
Final Survey: in 15 Tagen
Zeitpläne
Abschließen

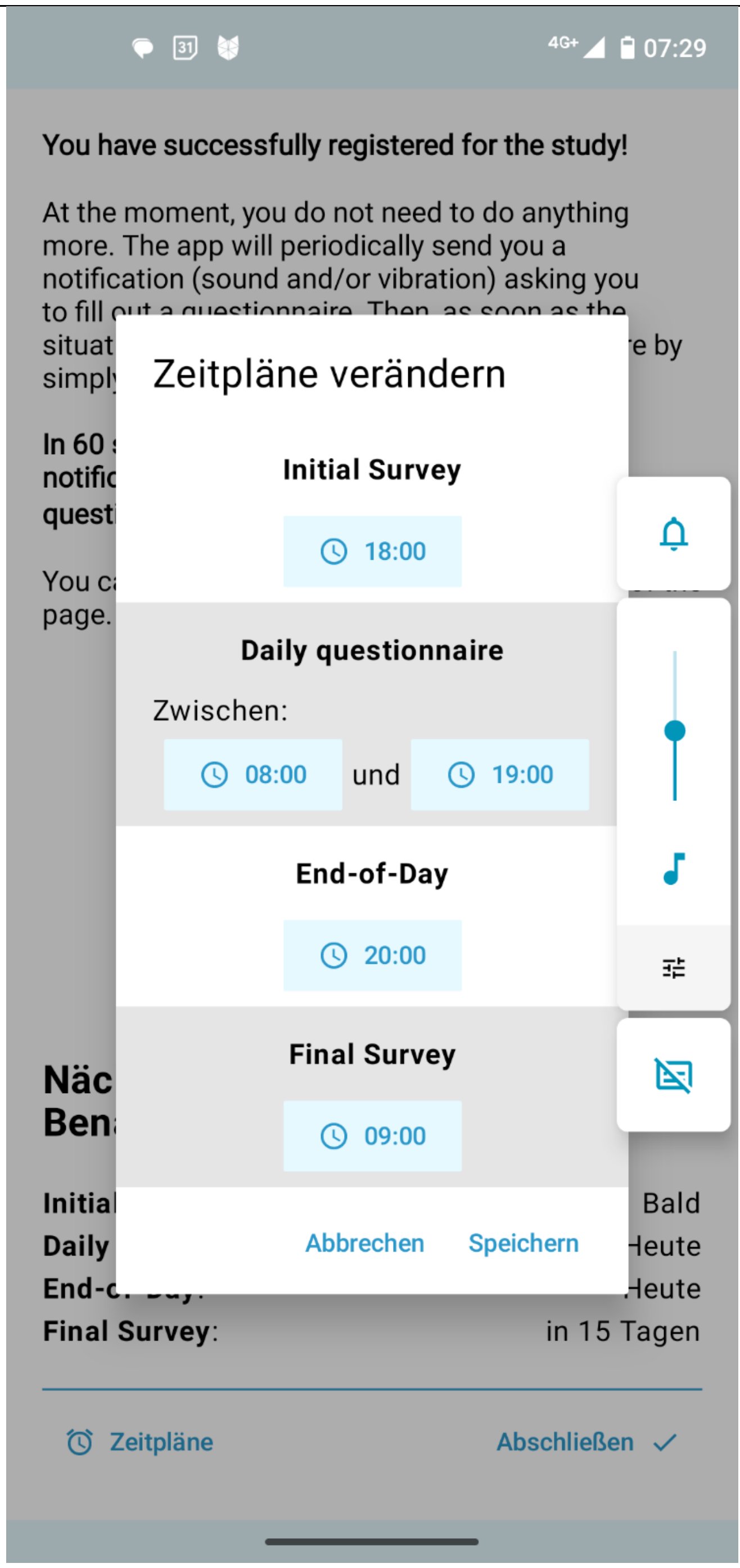
07:29
Zeitpläne verändern
Initial Survey
18:00
Daily questionnaire
Zwischen:
08:00
und
19:00
End-of-Day
20:00
Final Survey
09:00
Abbrechen
Speichern
Final Survey: in 15 Tagen
Zeitpläne
Abschließen

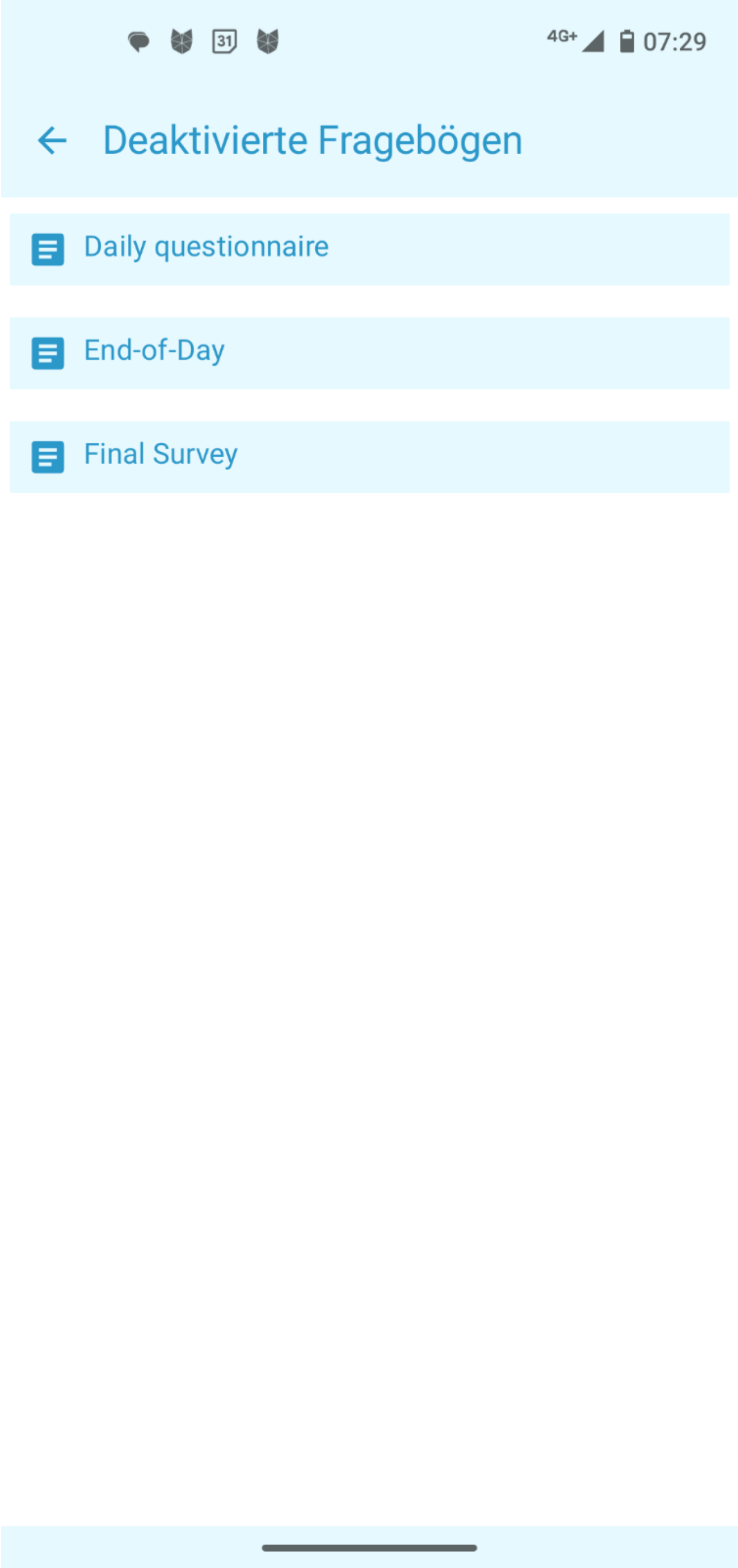
4G+ 07:29
Deaktivierte Fragebögen
Daily questionnaire
End-of-Day
Final Survey

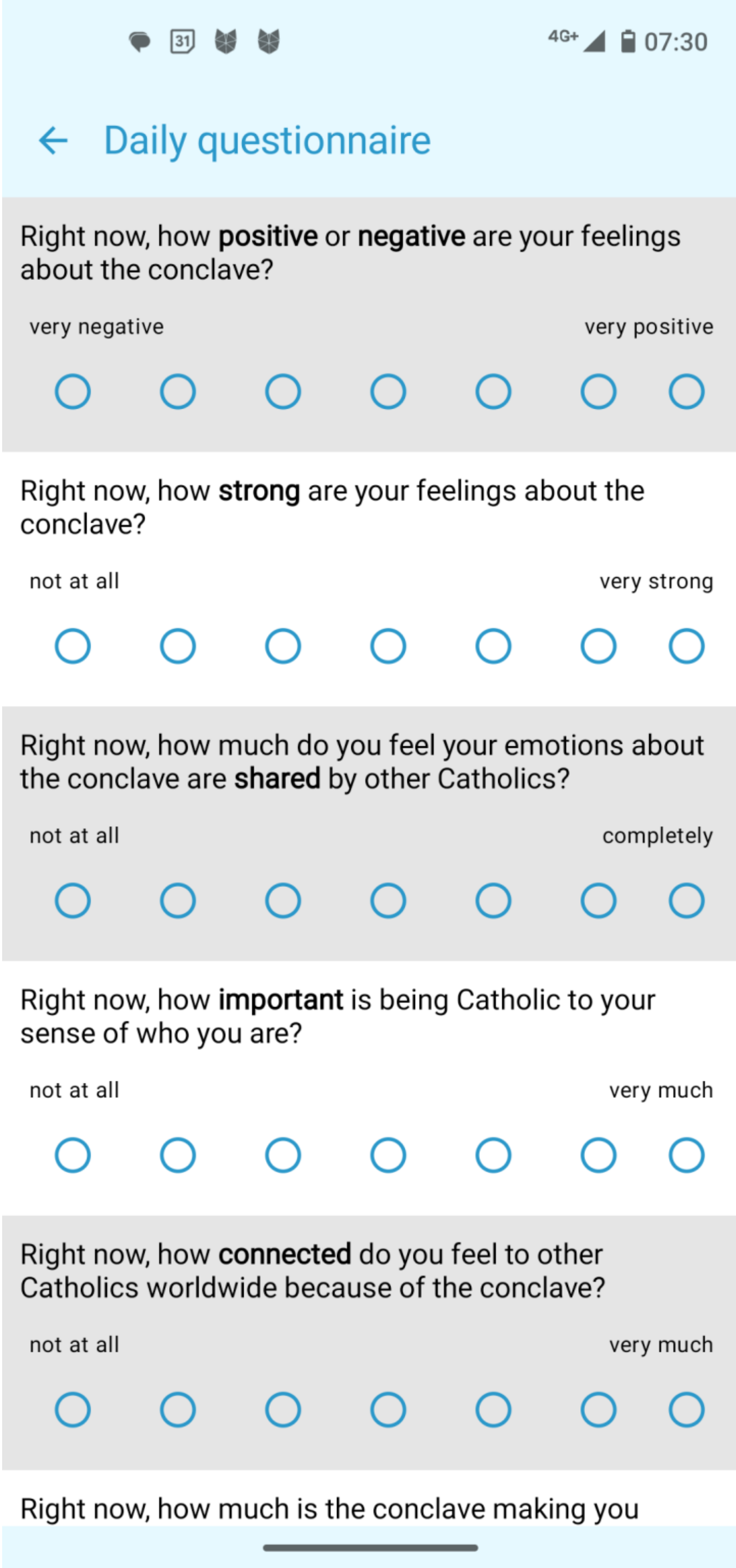
4G+ 07:30
Daily questionnaire
Right now, how **positive** or **negative** are your feelings about the conclave?
very negative
very positive
Right now, how **strong** are your feelings about the conclave?
not at all
very strong
Right now, how much do you feel your emotions about the conclave are **shared** by other Catholics?
not at all
completely
Right now, how **important** is being Catholic to your sense of who you are?
not at all
very much
Right now, how **connected** do you feel to other Catholics worldwide because of the conclave?
not at all
very much
Right now, how much is the conclave making you

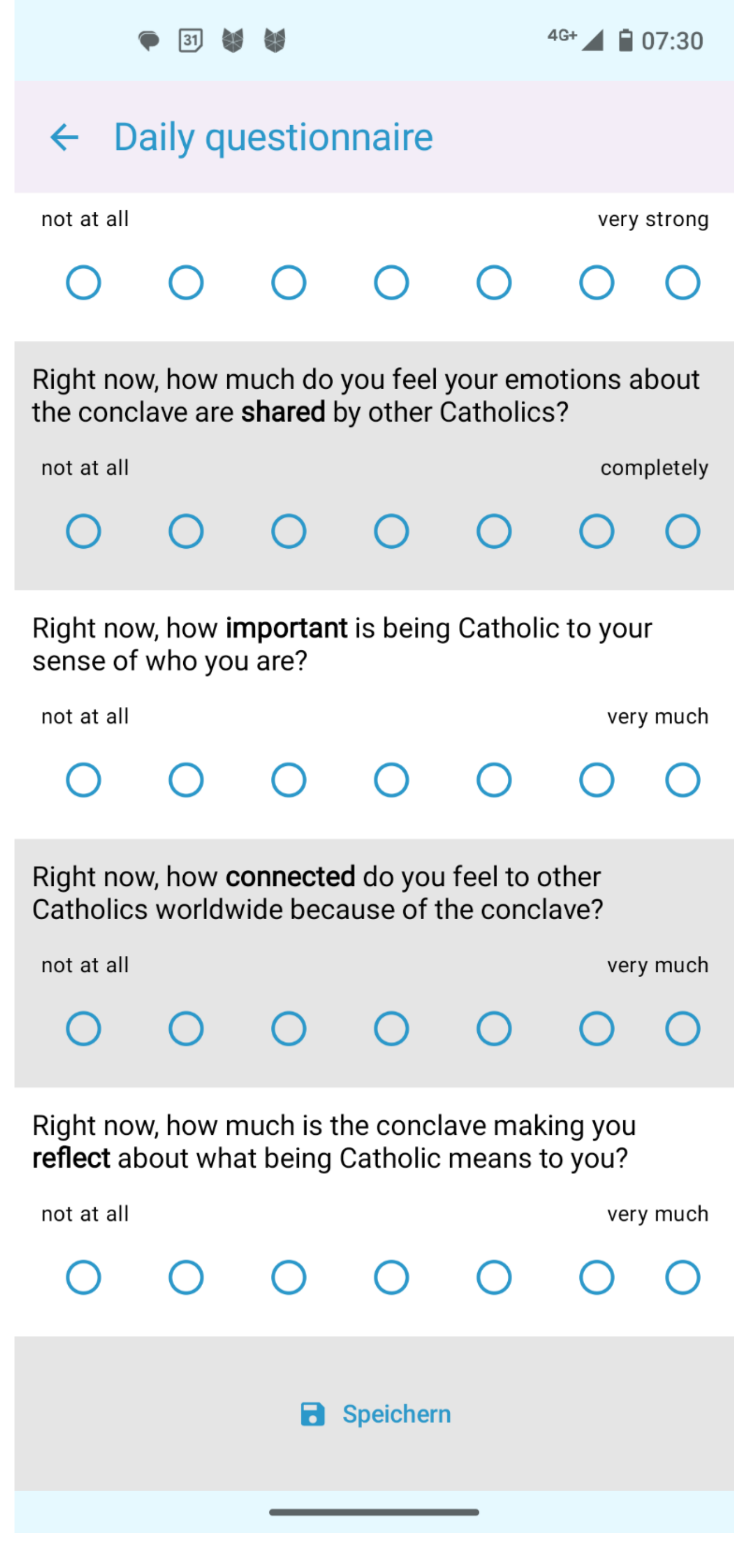

4G+ 07:30

Daily questionnaire

not at all — very strong

Right now, how much do you feel your emotions about the conclave are **shared** by other Catholics?

not at all — completely

Right now, how **important** is being Catholic to your sense of who you are?

not at all — very much

Right now, how **connected** do you feel to other Catholics worldwide because of the conclave?

not at all — very much

Right now, how much is the conclave making you **reflect** about what being Catholic means to you?

not at all — very much

Speichern

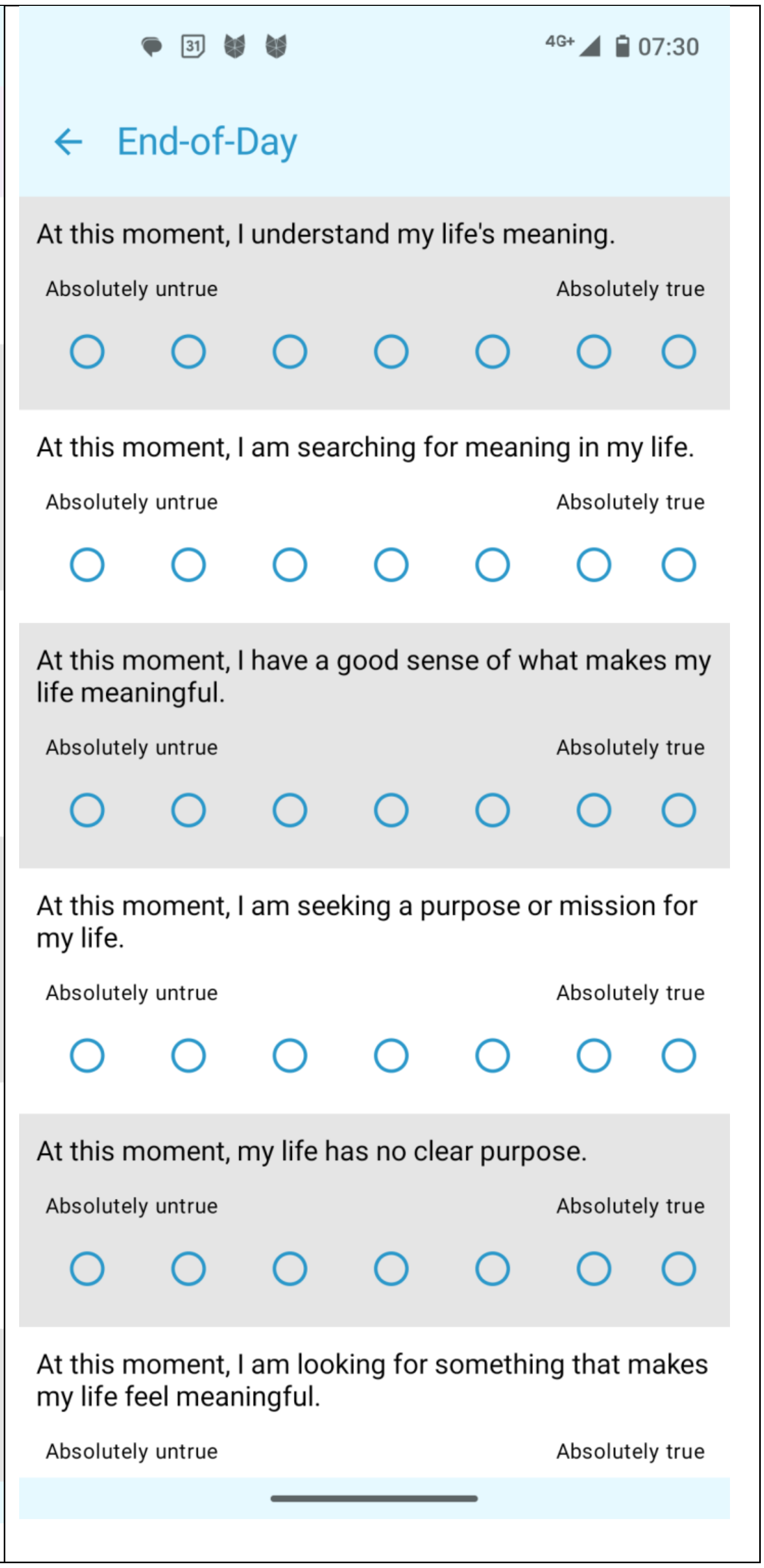

4G+ 07:30

End-of-Day

At this moment, I understand my life's meaning.

Absolutely untrue — Absolutely true

At this moment, I am searching for meaning in my life.

Absolutely untrue — Absolutely true

At this moment, I have a good sense of what makes my life meaningful.

Absolutely untrue — Absolutely true

At this moment, I am seeking a purpose or mission for my life.

Absolutely untrue — Absolutely true

At this moment, my life has no clear purpose.

Absolutely untrue — Absolutely true

At this moment, I am looking for something that makes my life feel meaningful.

Absolutely untrue — Absolutely true

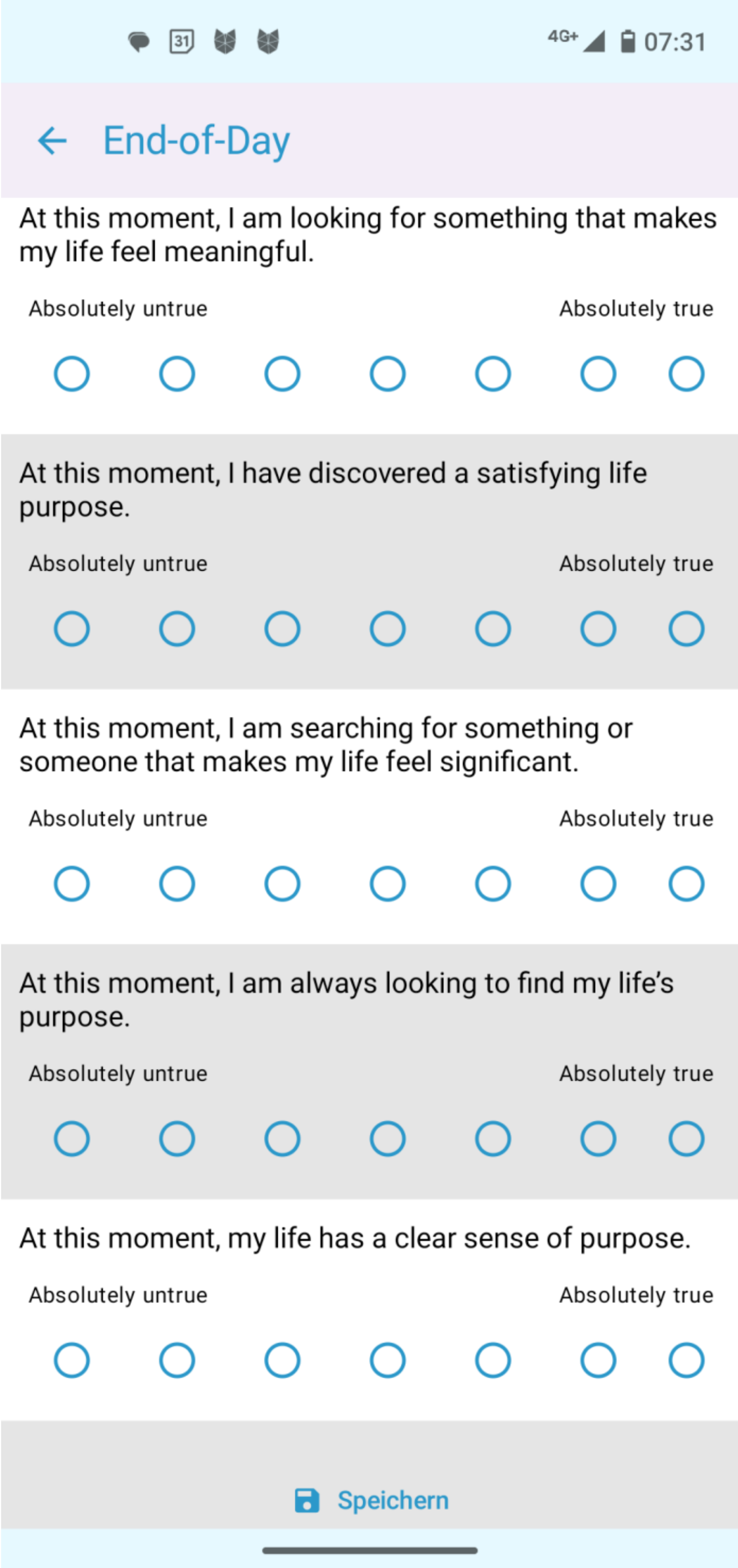
07:31
End-of-Day
At this moment, I am looking for something that makes my life feel meaningful.
Absolutely untrue
Absolutely true
At this moment, I have discovered a satisfying life purpose.
Absolutely untrue
Absolutely true
At this moment, I am searching for something or someone that makes my life feel significant.
Absolutely untrue
Absolutely true
At this moment, I am always looking to find my life's purpose.
Absolutely untrue
Absolutely true
At this moment, my life has a clear sense of purpose.
Absolutely untrue
Absolutely true
Speichern

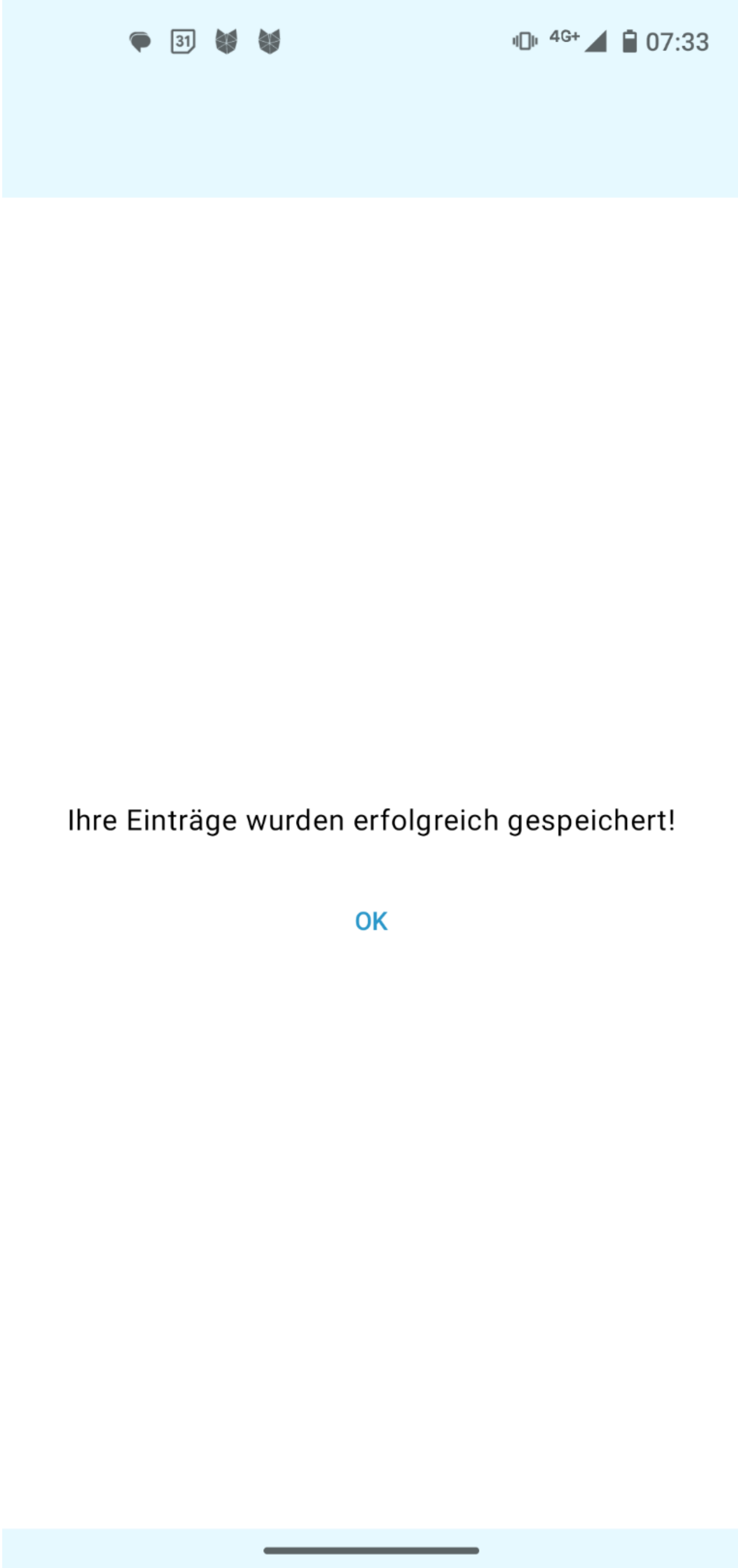
07:33
Ihre Einträge wurden erfolgreich gespeichert!
OK

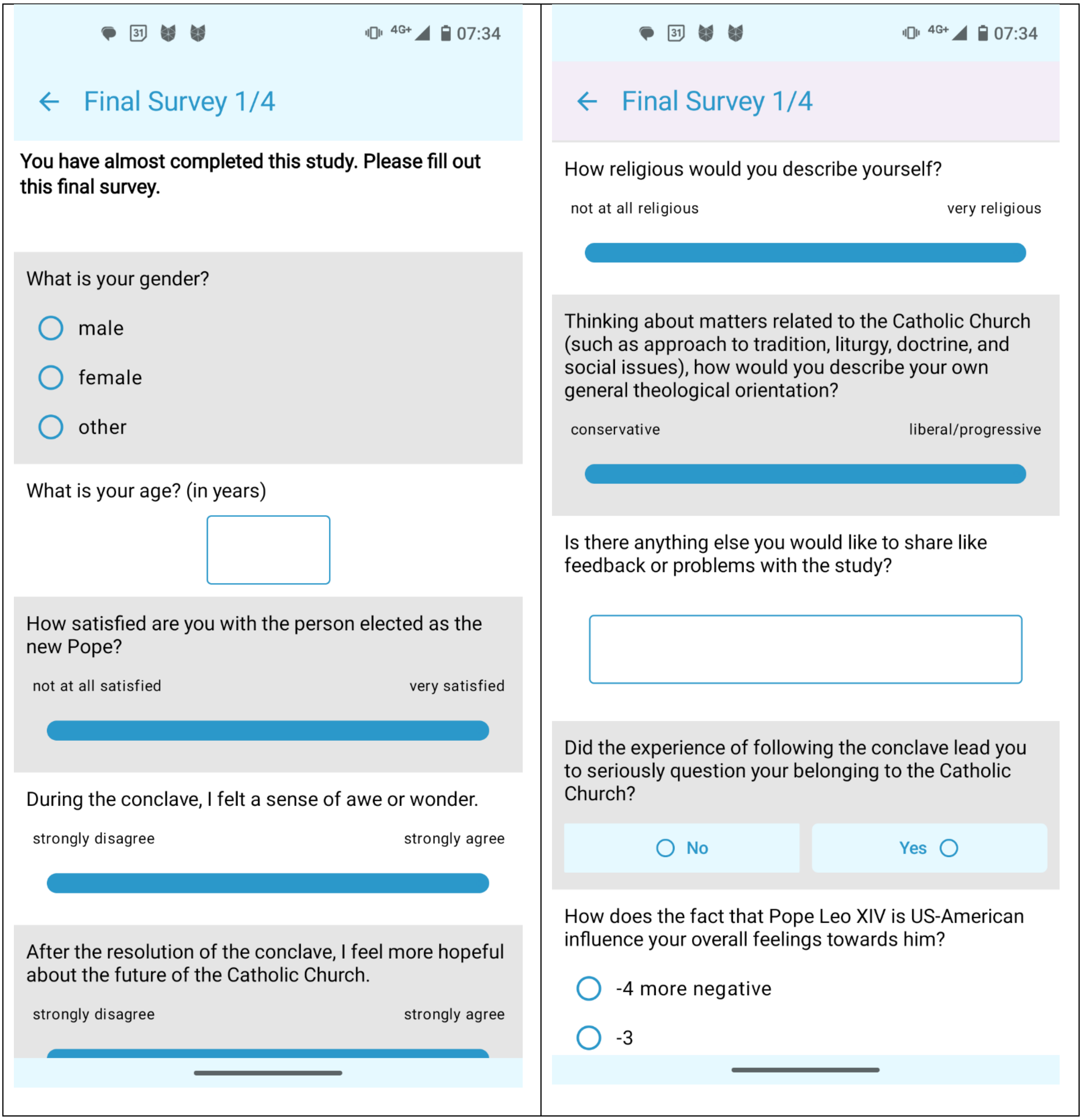
07:34
Final Survey 1/4
You have almost completed this study. Please fill out this final survey.
What is your gender?
male
female
other
What is your age? (in years)
How satisfied are you with the person elected as the new Pope?
not at all satisfied
very satisfied
During the conclave, I felt a sense of awe or wonder.
strongly disagree
strongly agree
After the resolution of the conclave, I feel more hopeful about the future of the Catholic Church.
strongly disagree
strongly agree
07:34
Final Survey 1/4
How religious would you describe yourself?
not at all religious
very religious
Thinking about matters related to the Catholic Church (such as approach to tradition, liturgy, doctrine, and social issues), how would you describe your own general theological orientation?
conservative
liberal/progressive
Is there anything else you would like to share like feedback or problems with the study?
Did the experience of following the conclave lead you to seriously question your belonging to the Catholic Church?
No
Yes
How does the fact that Pope Leo XIV is US-American influence your overall feelings towards him?
-4 more negative
-3

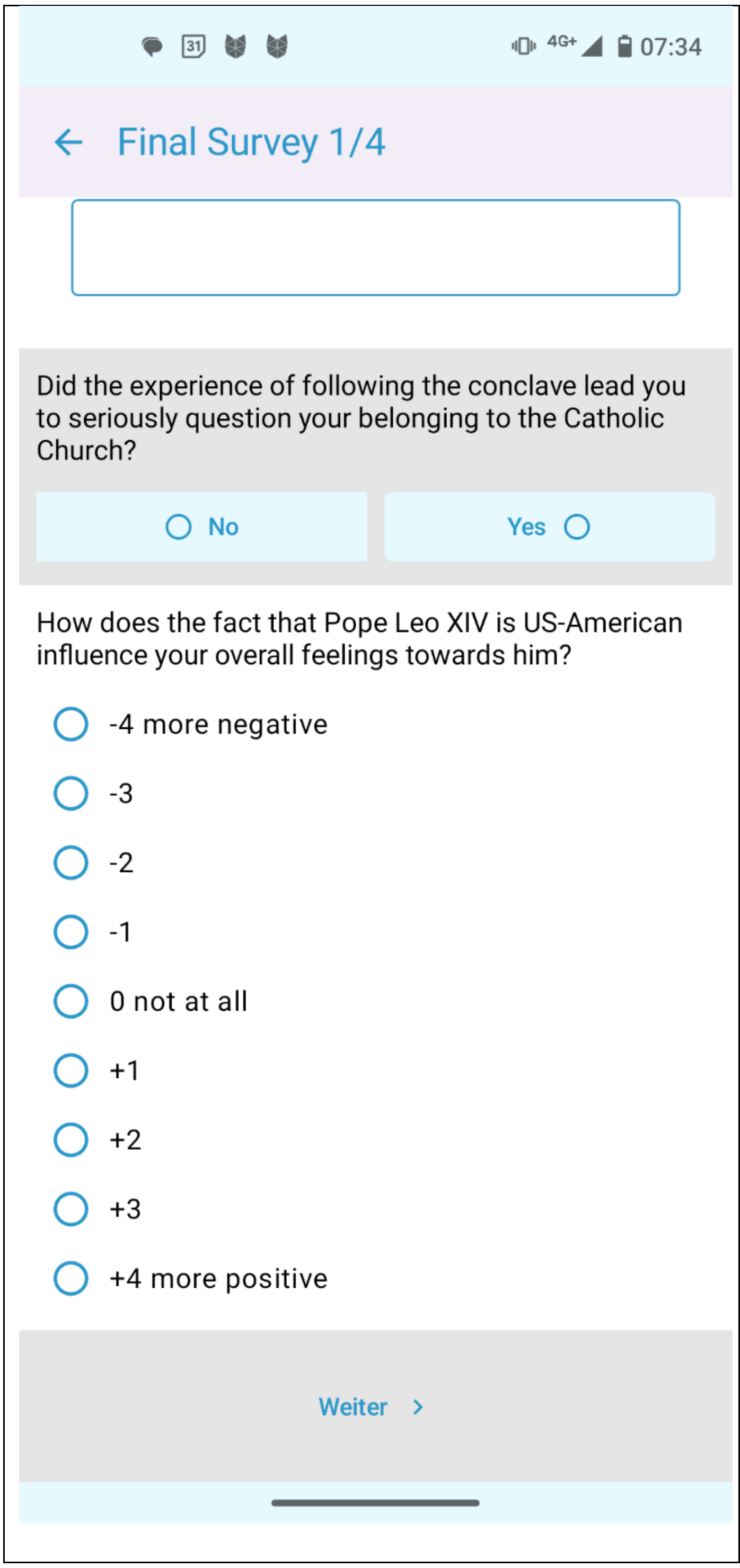

07:34

Final Survey 1/4

Did the experience of following the conclave lead you to seriously question your belonging to the Catholic Church?

No | Yes

How does the fact that Pope Leo XIV is US-American influence your overall feelings towards him?

- -4 more negative
- -3
- -2
- -1
- 0 not at all
- +1
- +2
- +3
- +4 more positive

Weiter >

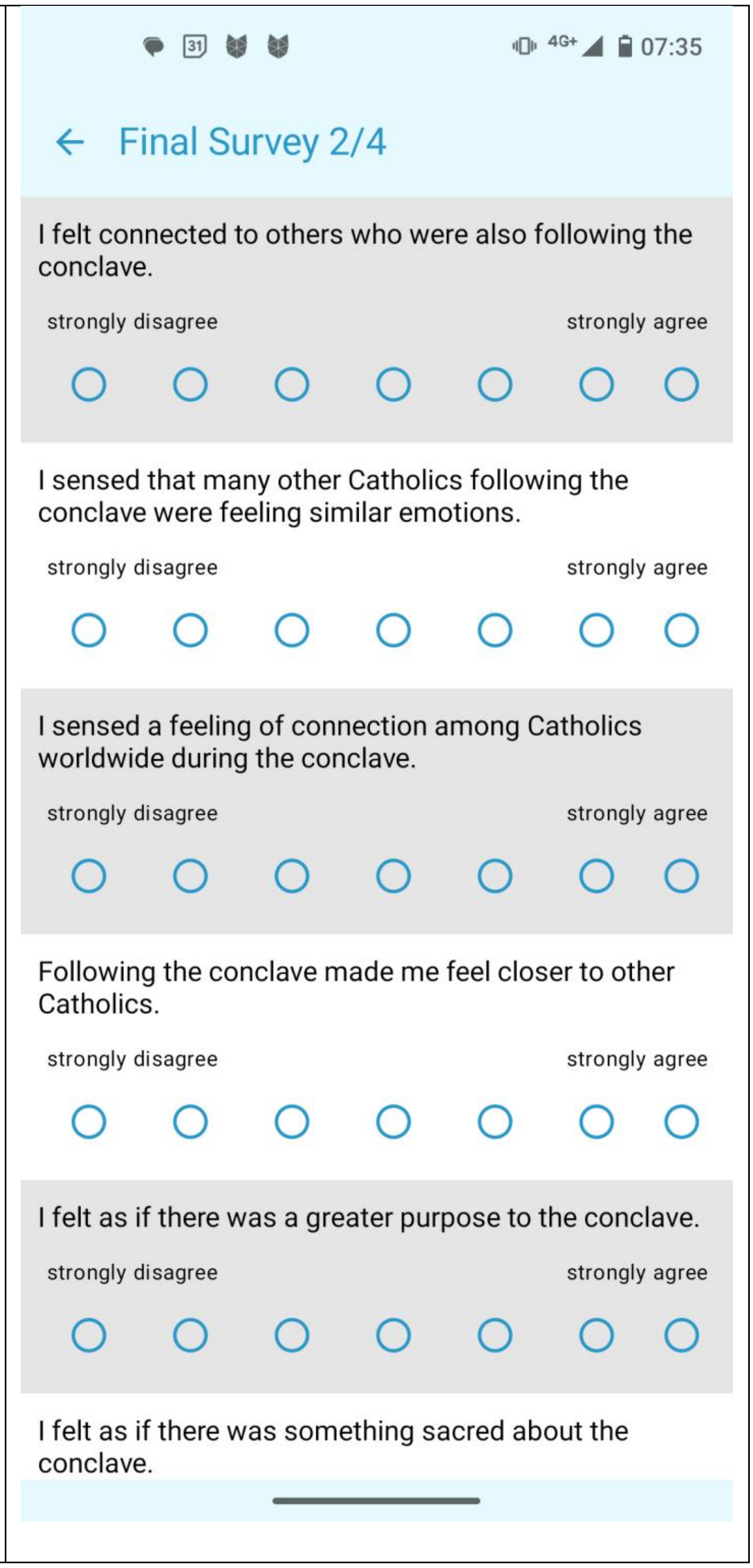

07:35

Final Survey 2/4

I felt connected to others who were also following the conclave.

strongly disagree — strongly agree

I sensed that many other Catholics following the conclave were feeling similar emotions.

strongly disagree — strongly agree

I sensed a feeling of connection among Catholics worldwide during the conclave.

strongly disagree — strongly agree

Following the conclave made me feel closer to other Catholics.

strongly disagree — strongly agree

I felt as if there was a greater purpose to the conclave.

strongly disagree — strongly agree

I felt as if there was something sacred about the conclave.

07:35

Final Survey 2/4

Catholics.

strongly disagree — strongly agree

○ ○ ○ ○ ○ ○ ○

I felt as if there was a greater purpose to the conclave.

strongly disagree — strongly agree

○ ○ ○ ○ ○ ○ ○

I felt as if there was something sacred about the conclave.

strongly disagree — strongly agree

○ ○ ○ ○ ○ ○ ○

I felt as if there was something special about the conclave.

strongly disagree — strongly agree

○ ○ ○ ○ ○ ○ ○

I felt as if following the conclave changed me in some way.

strongly disagree — strongly agree

○ ○ ○ ○ ○ ○ ○

Weiter >

07:35

Final Survey 3/4

Thinking back over the conclave period, how positive or negative were your feelings related to it, on average?

Interested

Very slightly or not at all — Extremely

○ ○ ○ ○ ○

Excited

Very slightly or not at all — Extremely

○ ○ ○ ○ ○

Strong

Very slightly or not at all — Extremely

○ ○ ○ ○ ○

Enthusiastic

Very slightly or not at all — Extremely

○ ○ ○ ○ ○

Proud

Very slightly or not at all — Extremely

○ ○ ○ ○ ○

Alert

Very slightly or not at all — Extremely

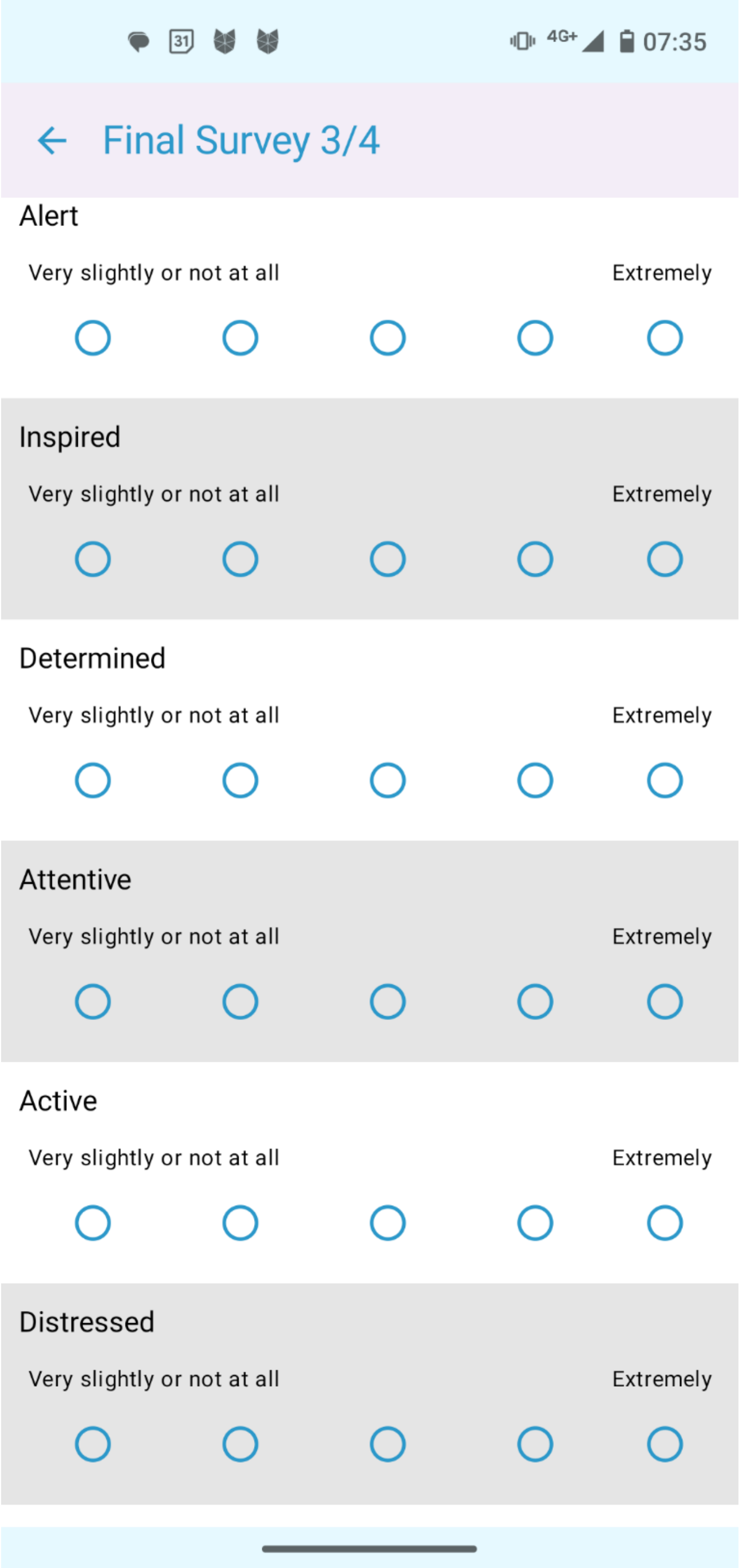
07:35
Final Survey 3/4
Alert
Very slightly or not at all
Extremely
Inspired
Very slightly or not at all
Extremely
Determined
Very slightly or not at all
Extremely
Attentive
Very slightly or not at all
Extremely
Active
Very slightly or not at all
Extremely
Distressed
Very slightly or not at all
Extremely

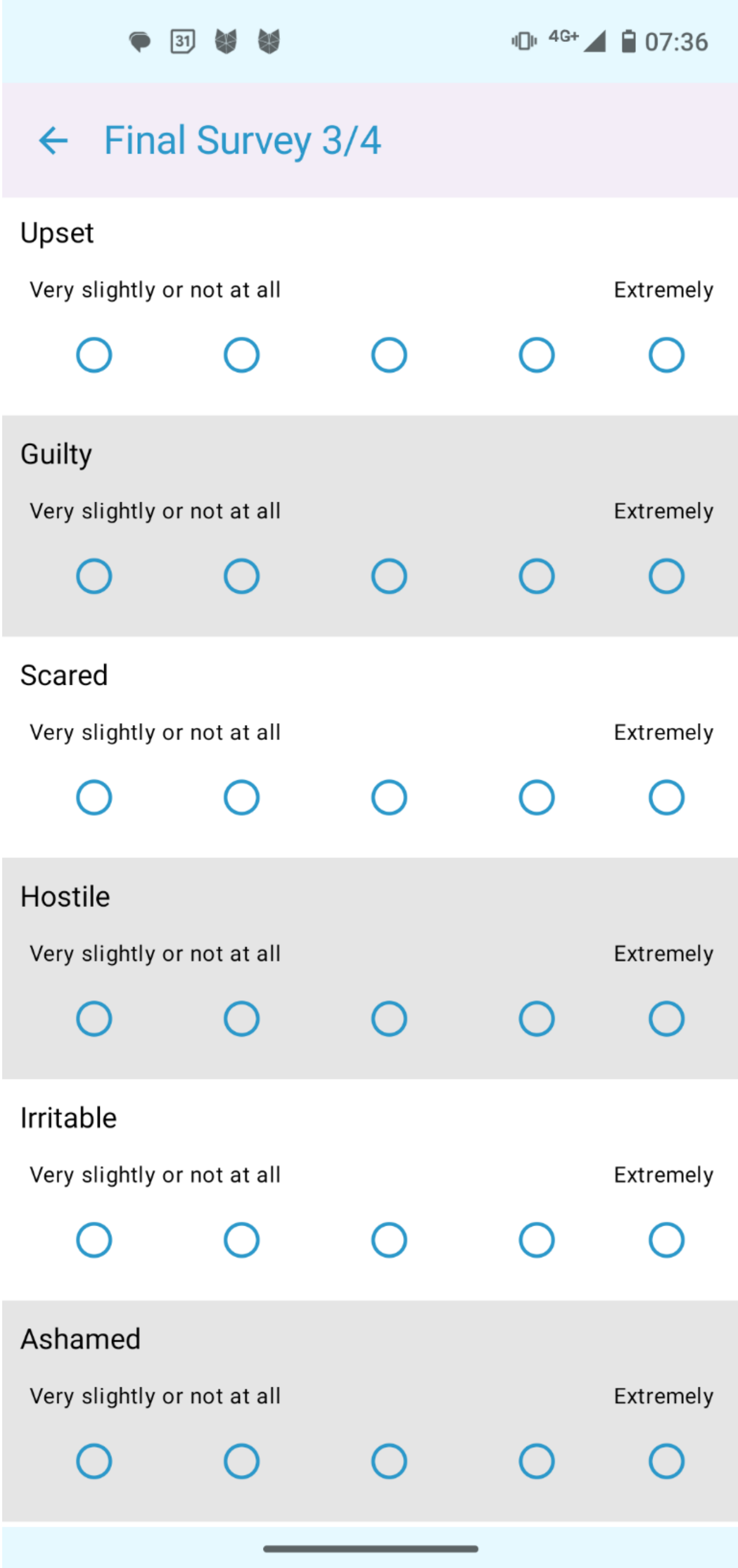
07:36
Final Survey 3/4
Upset
Very slightly or not at all
Extremely
Guilty
Very slightly or not at all
Extremely
Scared
Very slightly or not at all
Extremely
Hostile
Very slightly or not at all
Extremely
Irritable
Very slightly or not at all
Extremely
Ashamed
Very slightly or not at all
Extremely

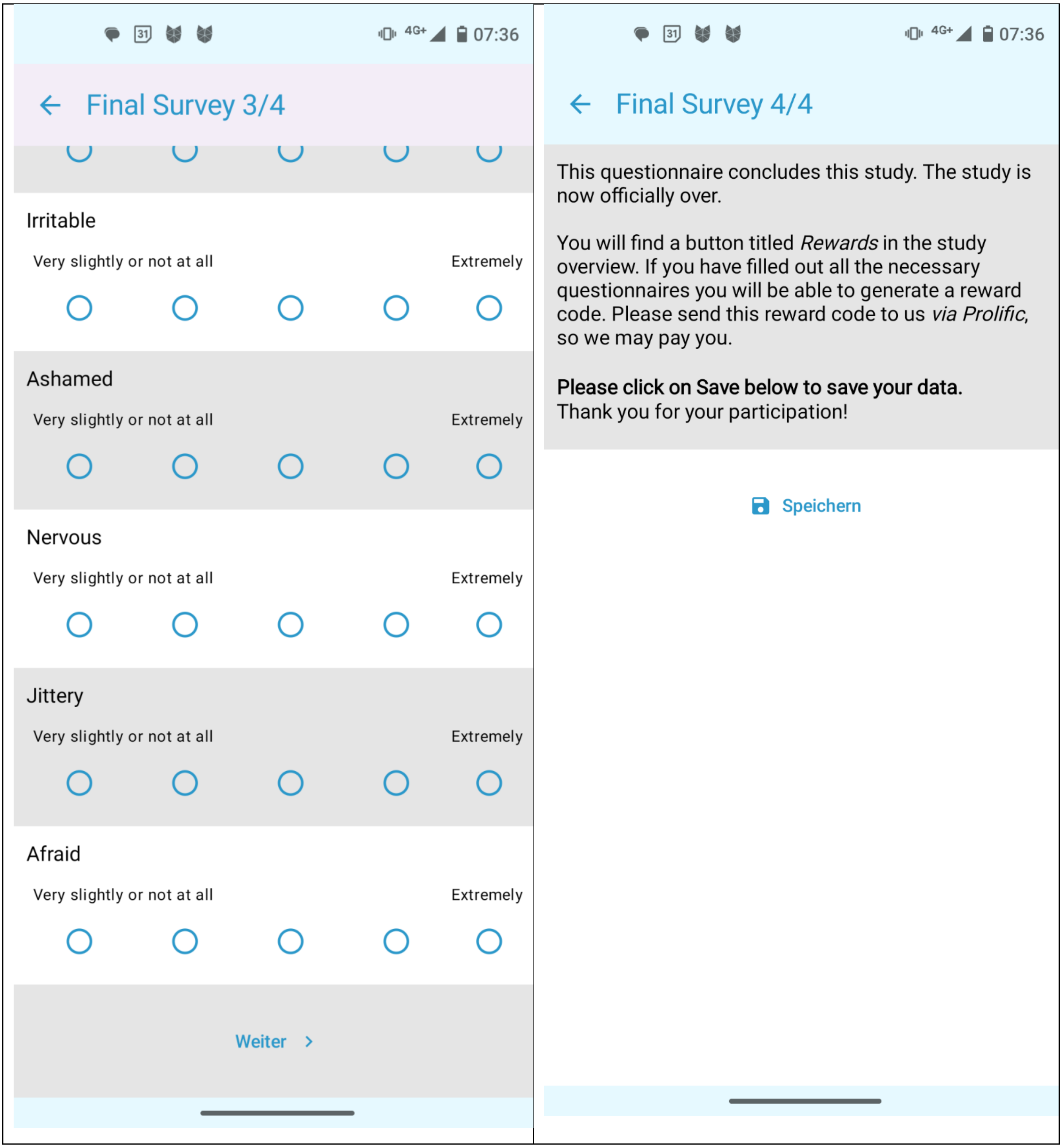
07:36
Final Survey 3/4
Irritable
Very slightly or not at all
Extremely
Ashamed
Very slightly or not at all
Extremely
Nervous
Very slightly or not at all
Extremely
Jittery
Very slightly or not at all
Extremely
Afraid
Very slightly or not at all
Extremely
Weiter
07:36
Final Survey 4/4
This questionnaire concludes this study. The study is now officially over.
You will find a button titled *Rewards* in the study overview. If you have filled out all the necessary questionnaires you will be able to generate a reward code. Please send this reward code to us *via Prolific*, so we may pay you.
Please click on Save below to save your data.
Thank you for your participation!
Speichern

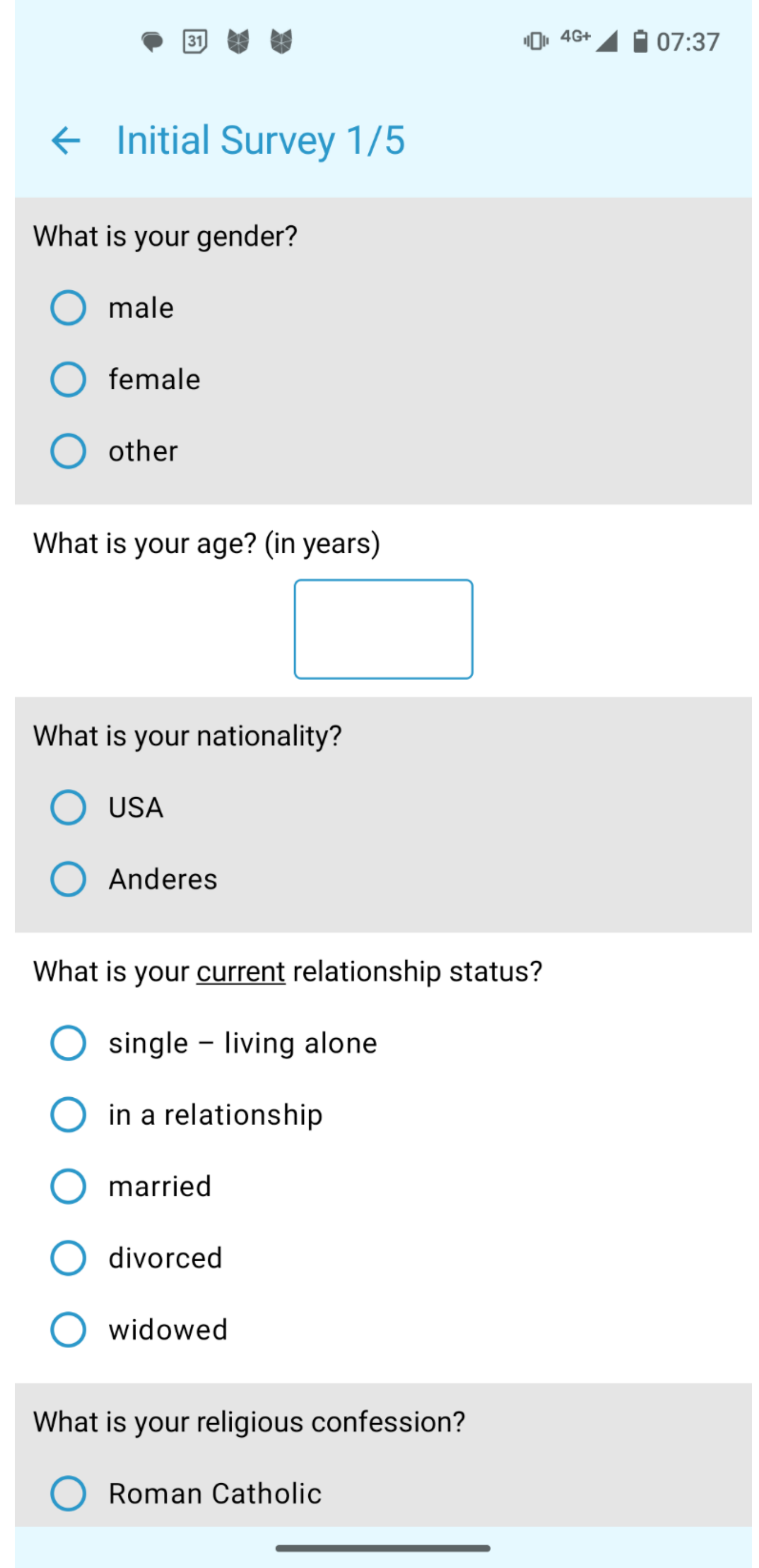

07:37

Initial Survey 1/5

What is your gender?

- male
- female
- other

What is your age? (in years)

What is your nationality?

- USA
- Anderes

What is your current relationship status?

- single – living alone
- in a relationship
- married
- divorced
- widowed

What is your religious confession?

- Roman Catholic

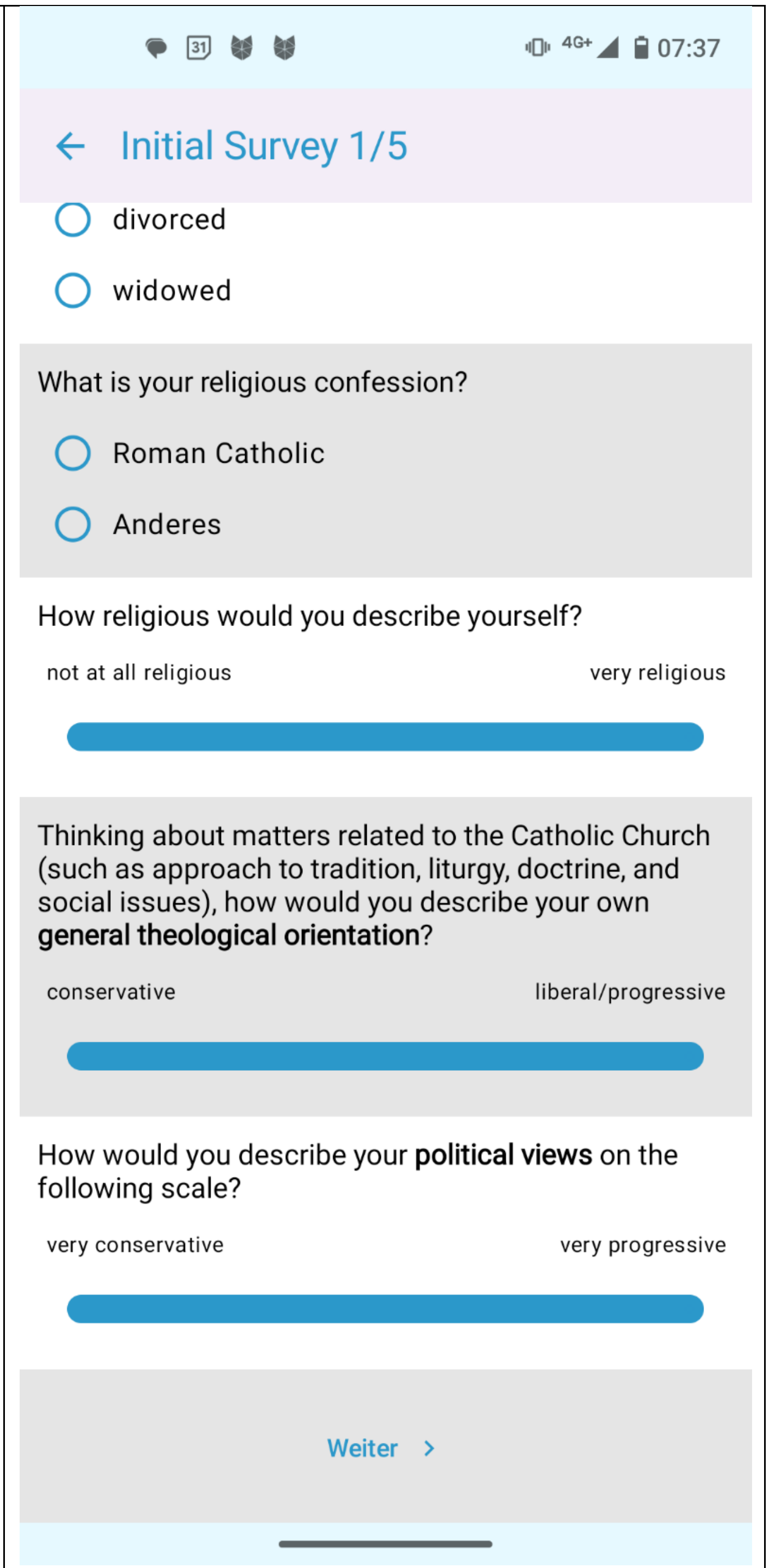

07:37

Initial Survey 1/5

- divorced
- widowed

What is your religious confession?

- Roman Catholic
- Anderes

How religious would you describe yourself?

not at all religious — very religious

Thinking about matters related to the Catholic Church (such as approach to tradition, liturgy, doctrine, and social issues), how would you describe your own **general theological orientation**?

conservative — liberal/progressive

How would you describe your **political views** on the following scale?

very conservative — very progressive

Weiter ›

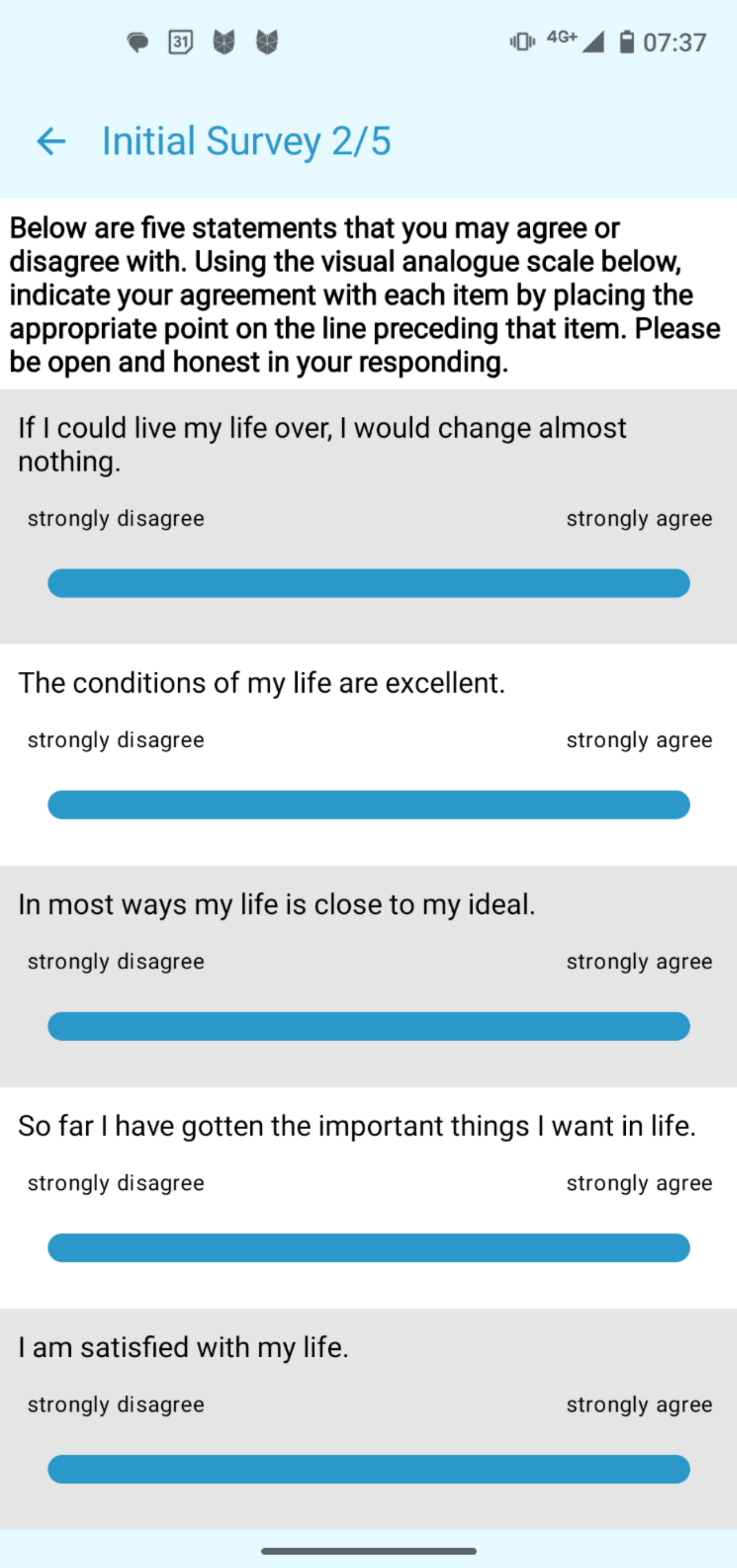
07:37
Initial Survey 2/5
Below are five statements that you may agree or disagree with. Using the visual analogue scale below, indicate your agreement with each item by placing the appropriate point on the line preceding that item. Please be open and honest in your responding.
If I could live my life over, I would change almost nothing.
strongly disagree
strongly agree
The conditions of my life are excellent.
strongly disagree
strongly agree
In most ways my life is close to my ideal.
strongly disagree
strongly agree
So far I have gotten the important things I want in life.
strongly disagree
strongly agree
I am satisfied with my life.
strongly disagree
strongly agree

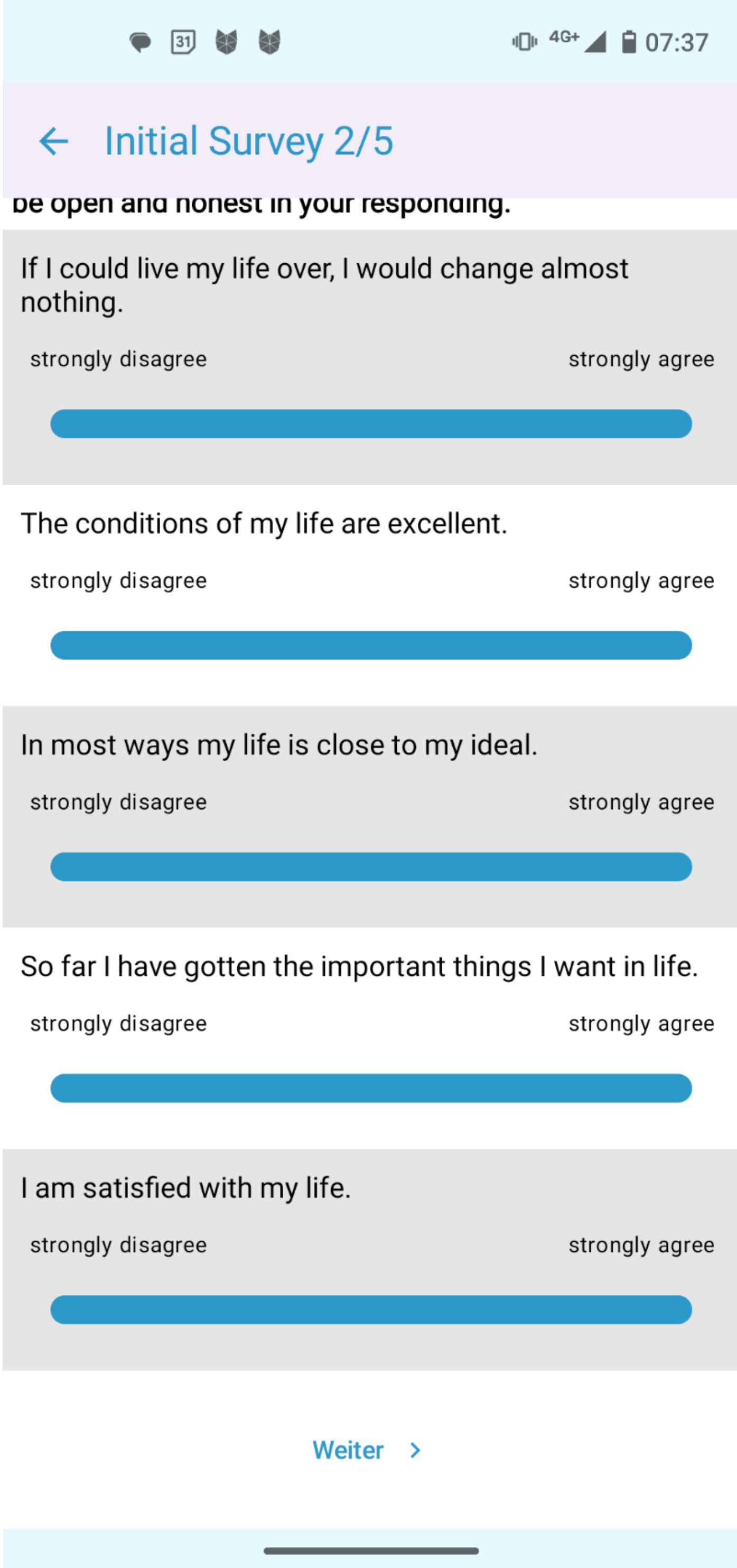
07:37
Initial Survey 2/5
be open and honest in your responding.
If I could live my life over, I would change almost nothing.
strongly disagree
strongly agree
The conditions of my life are excellent.
strongly disagree
strongly agree
In most ways my life is close to my ideal.
strongly disagree
strongly agree
So far I have gotten the important things I want in life.
strongly disagree
strongly agree
I am satisfied with my life.
strongly disagree
strongly agree
Weiter

11:21

Initial Survey 3/5

Now a few questions about your religiosity.

I make financial contributions to my religious organization.

not at all true for me — totally true to me

It is important to me to spend periods of time in private religious thought and reflection.

not at all true for me — totally true to me

Religion is especially important to me because it answers many questions about the meaning of life.

not at all true for me — totally true to me

Religious beliefs influence all my dealings in life.

not at all true for me — totally true to me

My religious beliefs lie behind my whole approach to life.

not at all true for me — totally true to me

11:21

Initial Survey 3/5

I enjoy working in the activities of my religious organization.

not at all true for me — totally true to me

I spend time trying to grow in understanding of my faith.

not at all true for me — totally true to me

I often read books and magazines about my faith.

not at all true for me — totally true to me

I enjoy spending time with others of my religious affiliation.

not at all true for me — totally true to me

I keep well informed about my local religious group and have some influence in its decisions.

not at all true for me — totally true to me

Weiter >

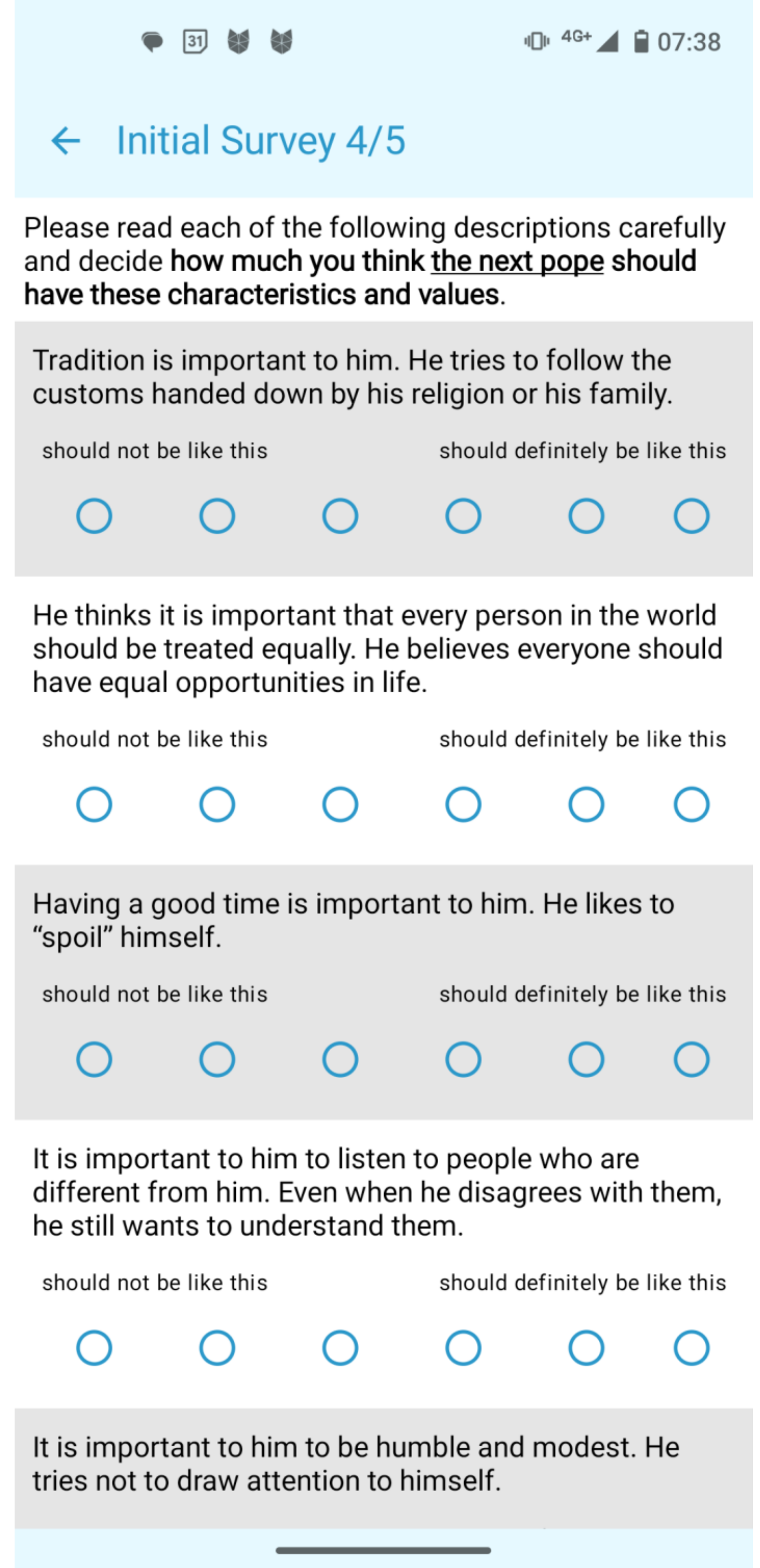

07:38

Initial Survey 4/5

Please read each of the following descriptions carefully and decide **how much you think the next pope should have these characteristics and values**.

Tradition is important to him. He tries to follow the customs handed down by his religion or his family.

should not be like this — should definitely be like this

○ ○ ○ ○ ○ ○

He thinks it is important that every person in the world should be treated equally. He believes everyone should have equal opportunities in life.

should not be like this — should definitely be like this

○ ○ ○ ○ ○ ○

Having a good time is important to him. He likes to "spoil" himself.

should not be like this — should definitely be like this

○ ○ ○ ○ ○ ○

It is important to him to listen to people who are different from him. Even when he disagrees with them, he still wants to understand them.

should not be like this — should definitely be like this

○ ○ ○ ○ ○ ○

It is important to him to be humble and modest. He tries not to draw attention to himself.

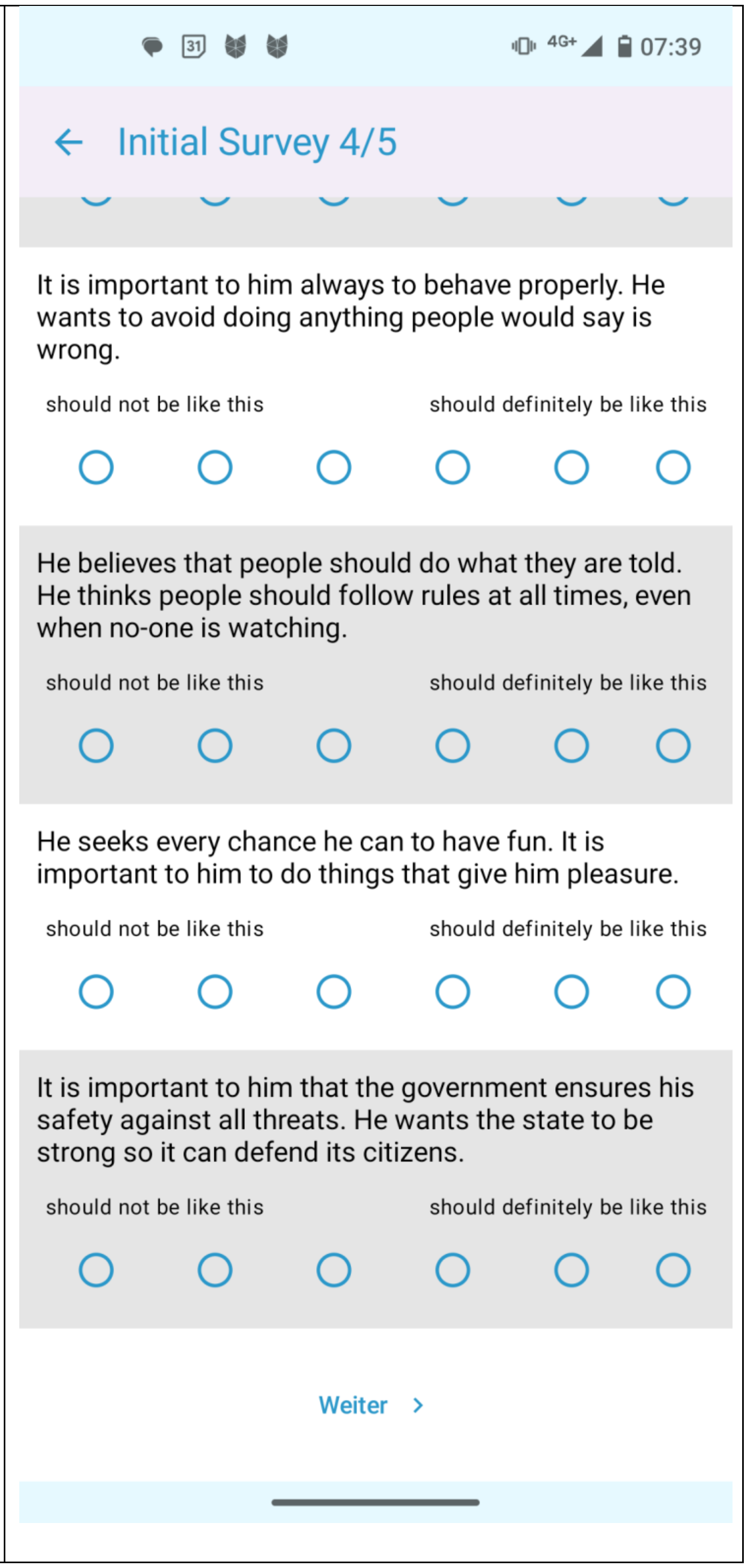

07:39

Initial Survey 4/5

It is important to him always to behave properly. He wants to avoid doing anything people would say is wrong.

should not be like this — should definitely be like this

○ ○ ○ ○ ○ ○

He believes that people should do what they are told. He thinks people should follow rules at all times, even when no-one is watching.

should not be like this — should definitely be like this

○ ○ ○ ○ ○ ○

He seeks every chance he can to have fun. It is important to him to do things that give him pleasure.

should not be like this — should definitely be like this

○ ○ ○ ○ ○ ○

It is important to him that the government ensures his safety against all threats. He wants the state to be strong so it can defend its citizens.

should not be like this — should definitely be like this

○ ○ ○ ○ ○ ○

Weiter ›

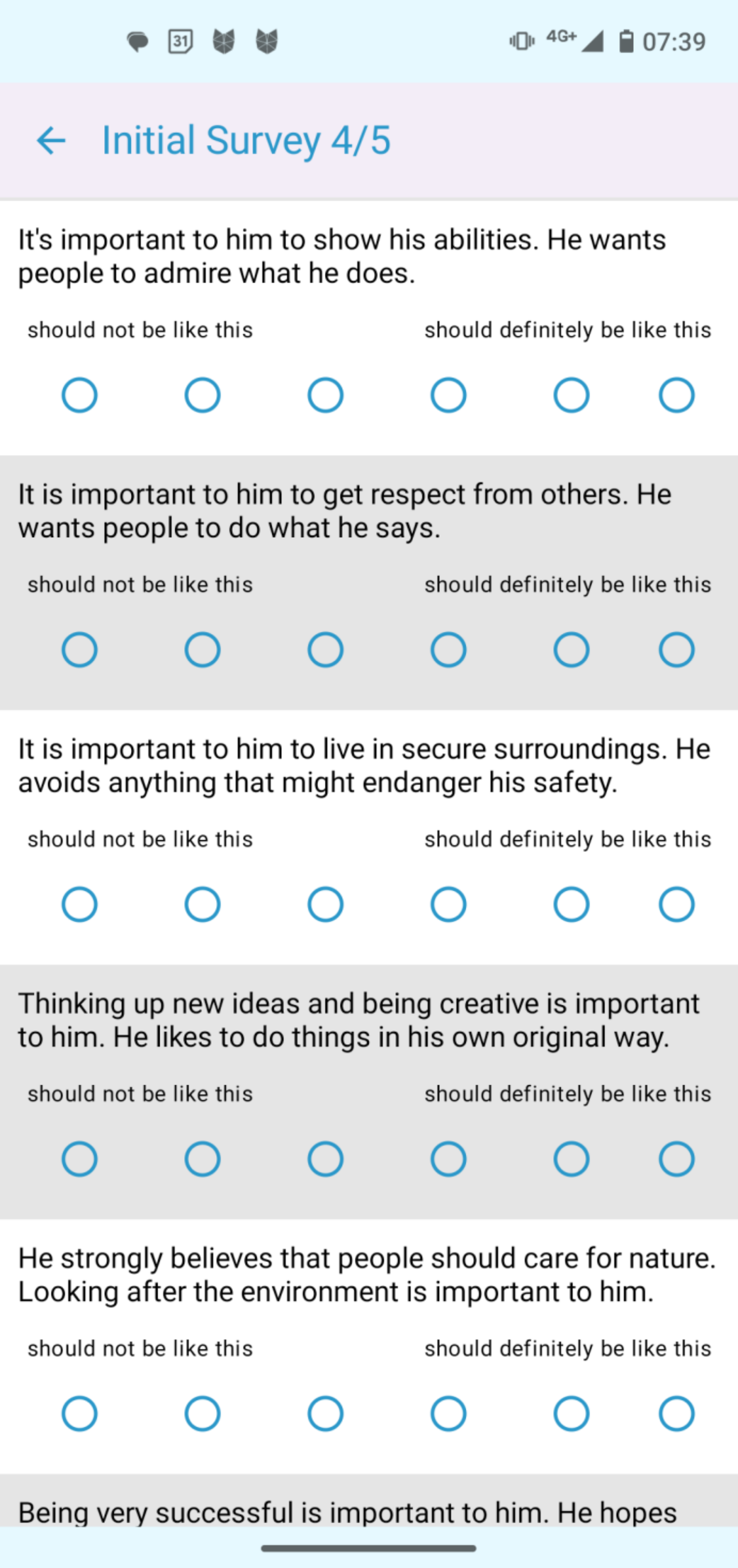

07:39

Initial Survey 4/5

It's important to him to show his abilities. He wants people to admire what he does.

should not be like this — should definitely be like this

It is important to him to get respect from others. He wants people to do what he says.

should not be like this — should definitely be like this

It is important to him to live in secure surroundings. He avoids anything that might endanger his safety.

should not be like this — should definitely be like this

Thinking up new ideas and being creative is important to him. He likes to do things in his own original way.

should not be like this — should definitely be like this

He strongly believes that people should care for nature. Looking after the environment is important to him.

should not be like this — should definitely be like this

Being very successful is important to him. He hopes

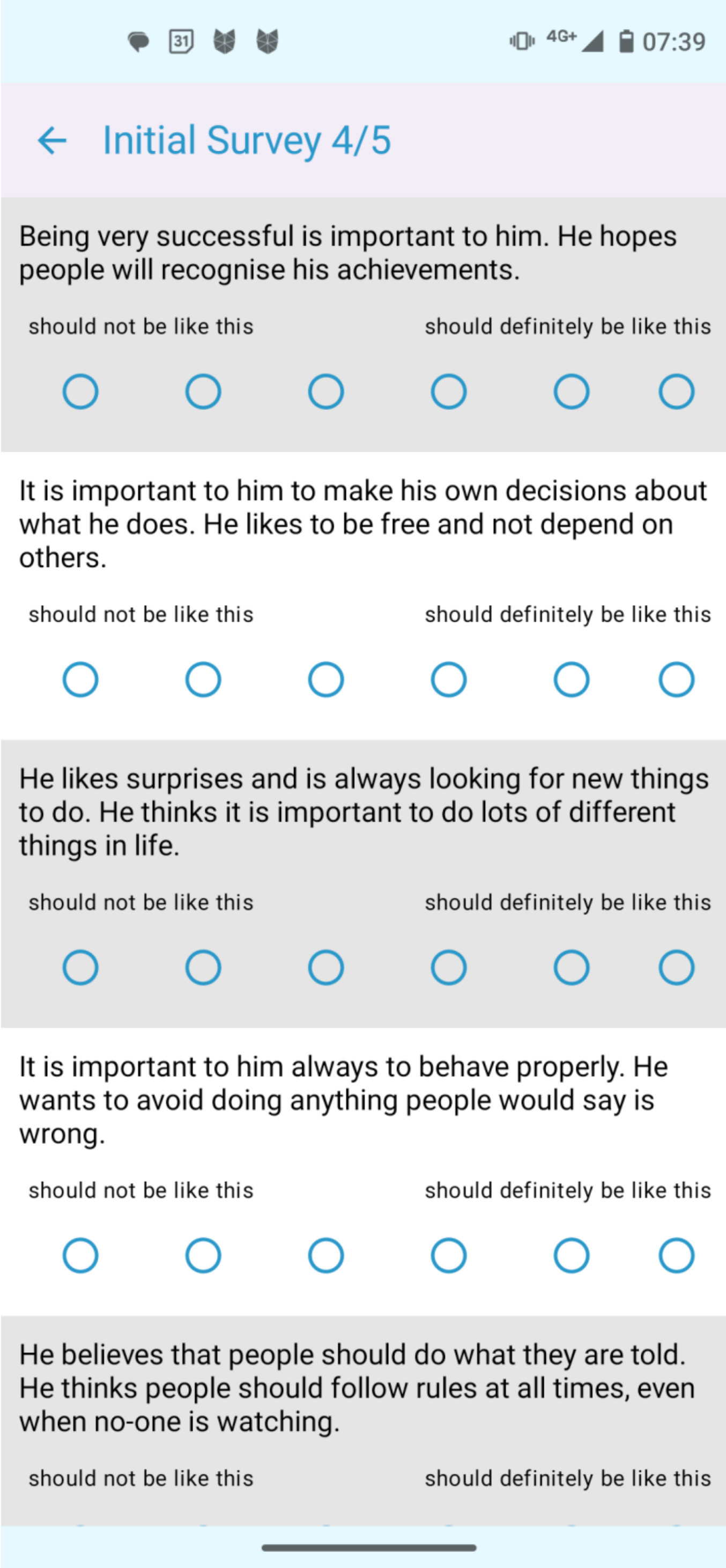

07:39

Initial Survey 4/5

Being very successful is important to him. He hopes people will recognise his achievements.

should not be like this — should definitely be like this

It is important to him to make his own decisions about what he does. He likes to be free and not depend on others.

should not be like this — should definitely be like this

He likes surprises and is always looking for new things to do. He thinks it is important to do lots of different things in life.

should not be like this — should definitely be like this

It is important to him always to behave properly. He wants to avoid doing anything people would say is wrong.

should not be like this — should definitely be like this

He believes that people should do what they are told. He thinks people should follow rules at all times, even when no-one is watching.

should not be like this — should definitely be like this

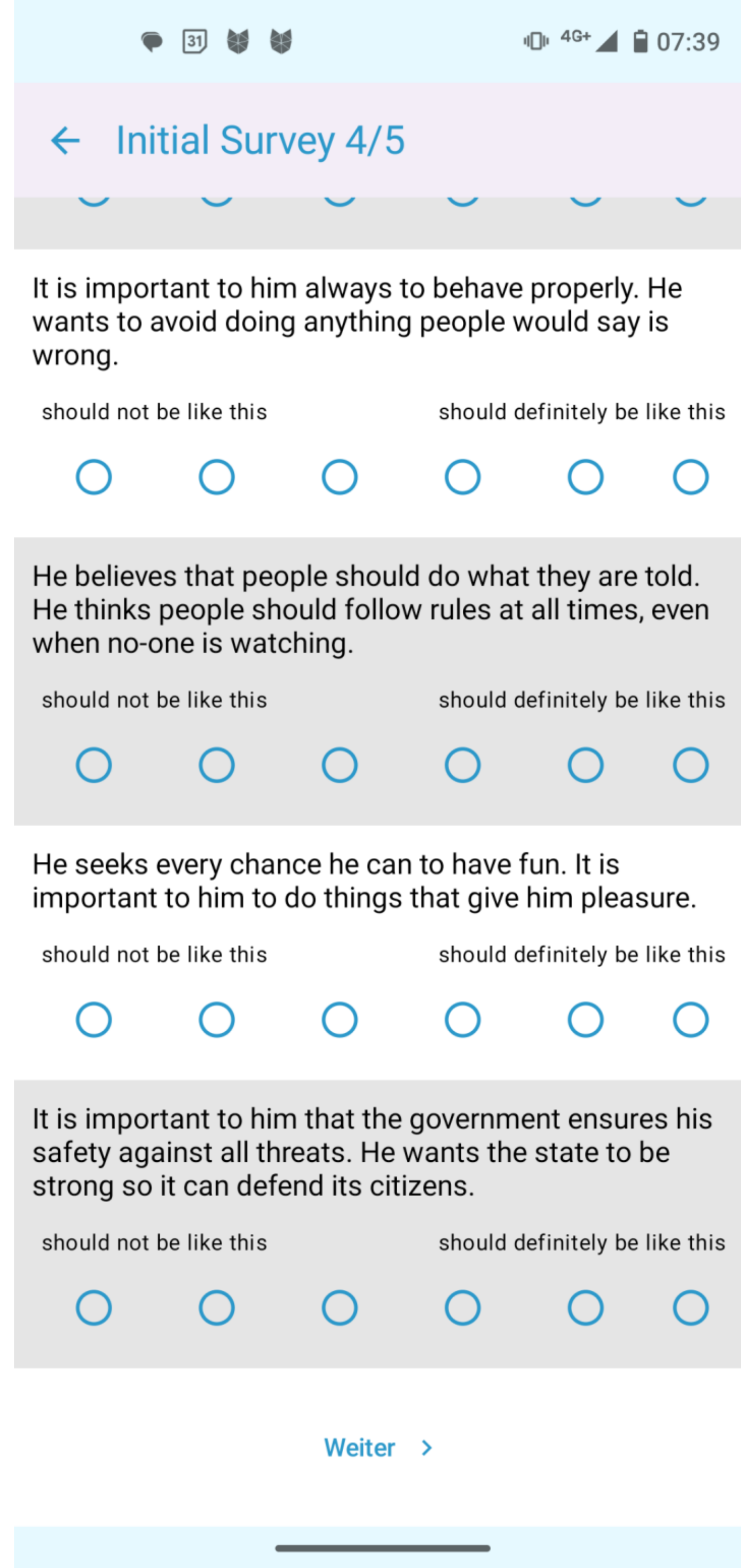
07:39
Initial Survey 4/5
It is important to him always to behave properly. He wants to avoid doing anything people would say is wrong.
should not be like this
should definitely be like this
He believes that people should do what they are told. He thinks people should follow rules at all times, even when no-one is watching.
should not be like this
should definitely be like this
He seeks every chance he can to have fun. It is important to him to do things that give him pleasure.
should not be like this
should definitely be like this
It is important to him that the government ensures his safety against all threats. He wants the state to be strong so it can defend its citizens.
should not be like this
should definitely be like this
Weiter

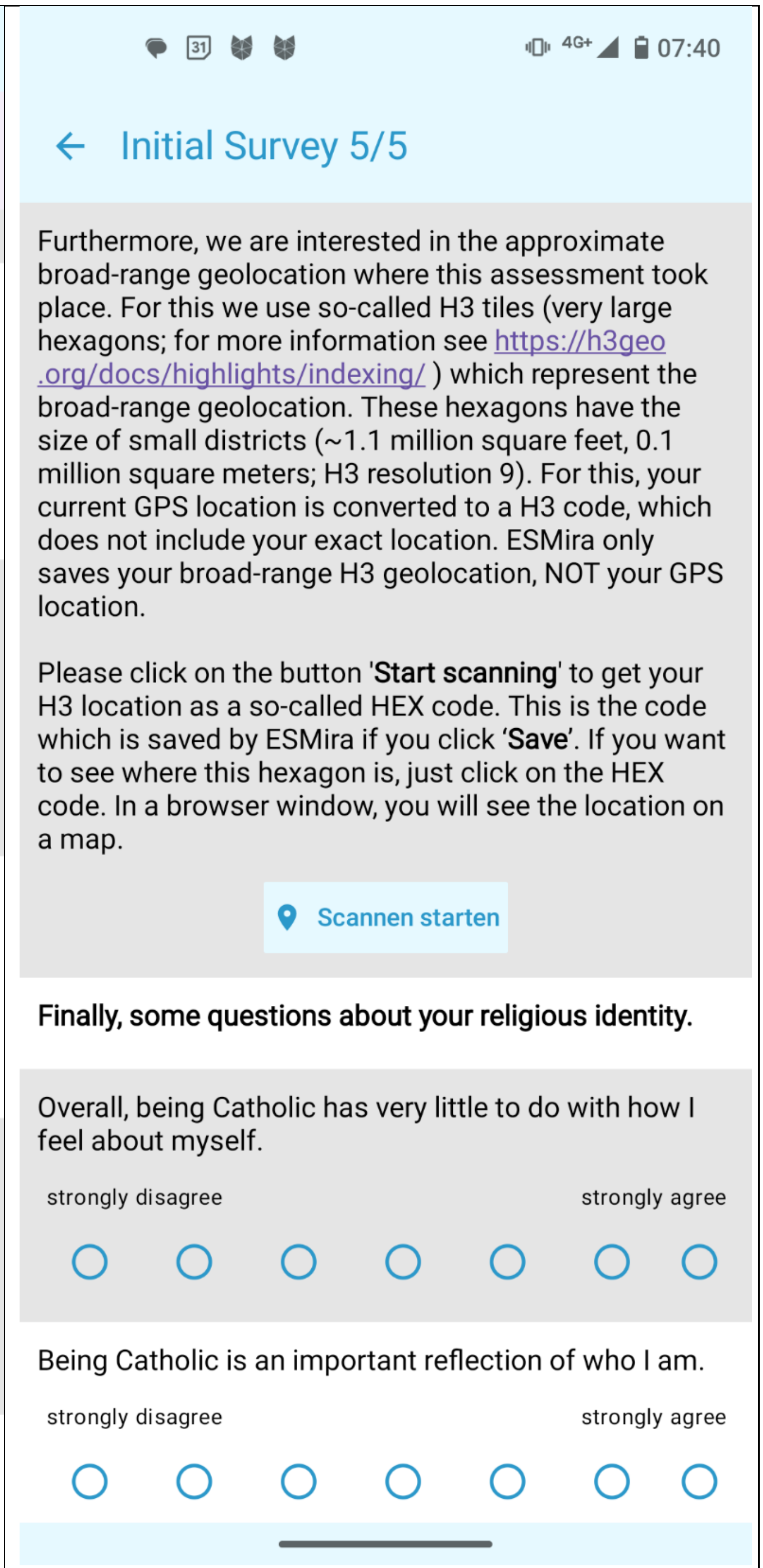
07:40
Initial Survey 5/5
Furthermore, we are interested in the approximate broad-range geolocation where this assessment took place. For this we use so-called H3 tiles (very large hexagons; for more information see https://h3geo.org/docs/highlights/indexing/) which represent the broad-range geolocation. These hexagons have the size of small districts (~1.1 million square feet, 0.1 million square meters; H3 resolution 9). For this, your current GPS location is converted to a H3 code, which does not include your exact location. ESMira only saves your broad-range H3 geolocation, NOT your GPS location.
Please click on the button 'Start scanning' to get your H3 location as a so-called HEX code. This is the code which is saved by ESMira if you click 'Save'. If you want to see where this hexagon is, just click on the HEX code. In a browser window, you will see the location on a map.
Scannen starten
Finally, some questions about your religious identity.
Overall, being Catholic has very little to do with how I feel about myself.
strongly disagree
strongly agree
Being Catholic is an important reflection of who I am.
strongly disagree
strongly agree

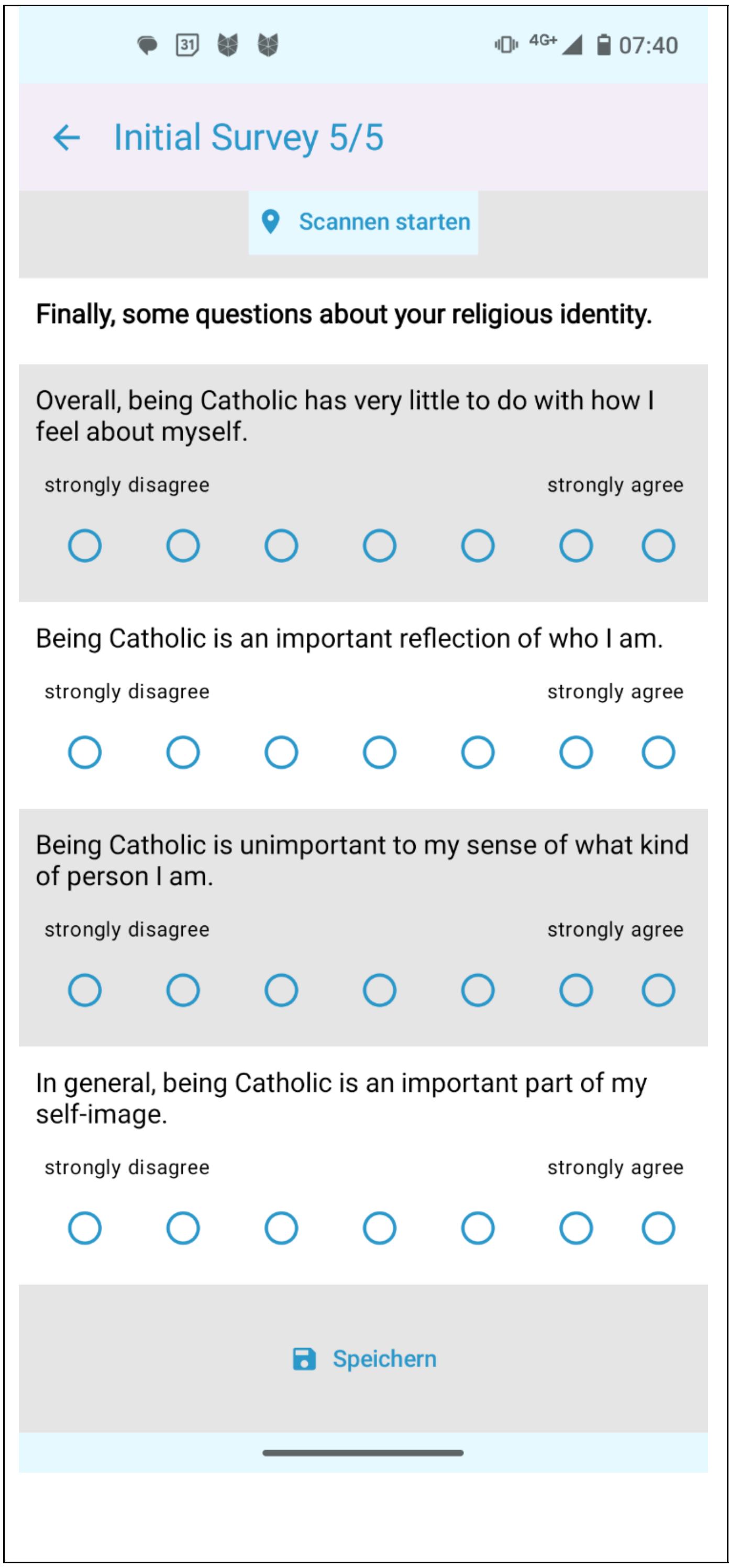
07:40
Initial Survey 5/5
Scannen starten
Finally, some questions about your religious identity.
Overall, being Catholic has very little to do with how I feel about myself.
strongly disagree
strongly agree
Being Catholic is an important reflection of who I am.
strongly disagree
strongly agree
Being Catholic is unimportant to my sense of what kind of person I am.
strongly disagree
strongly agree
In general, being Catholic is an important part of my self-image.
strongly disagree
strongly agree
Speichern

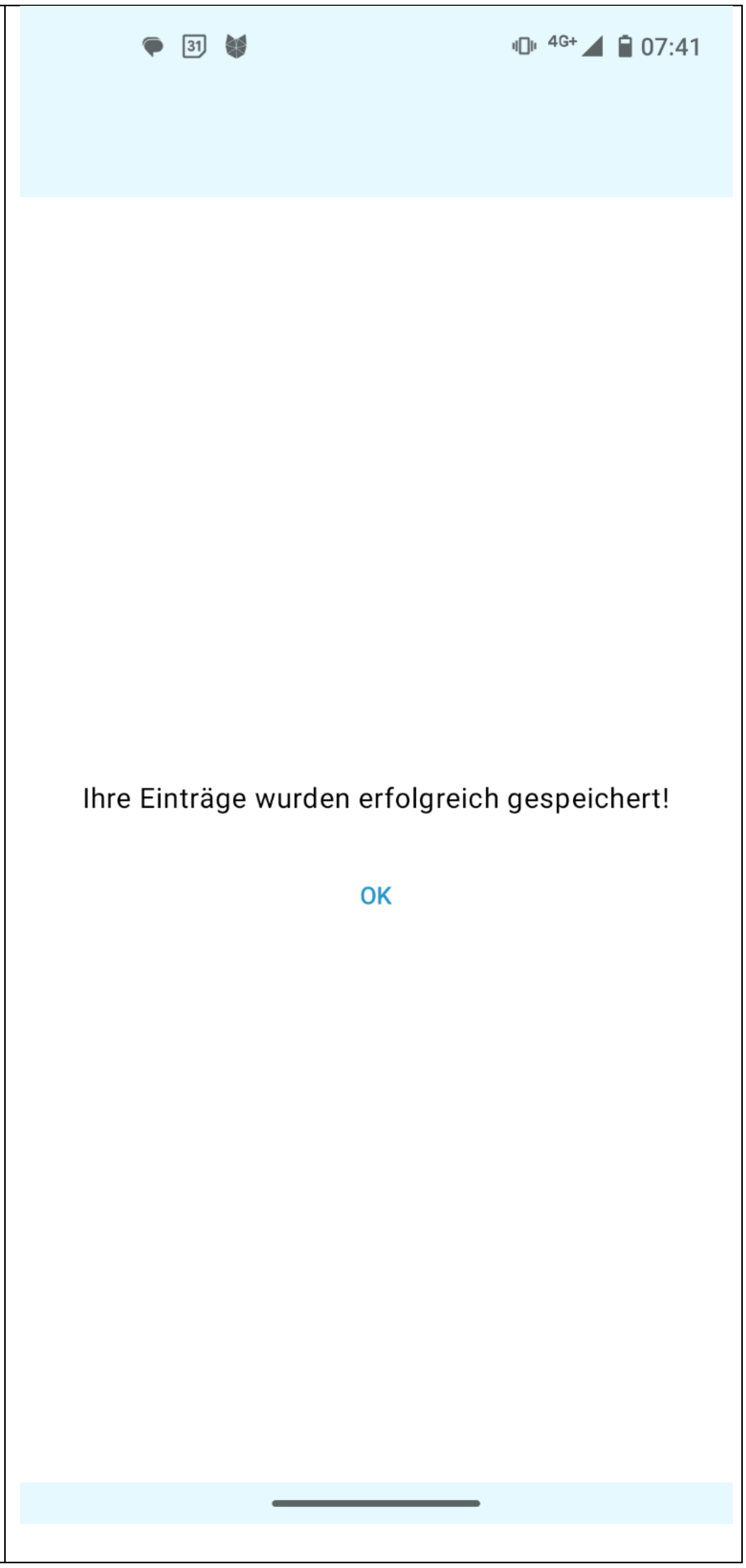
07:41
Ihre Einträge wurden erfolgreich gespeichert!
OK

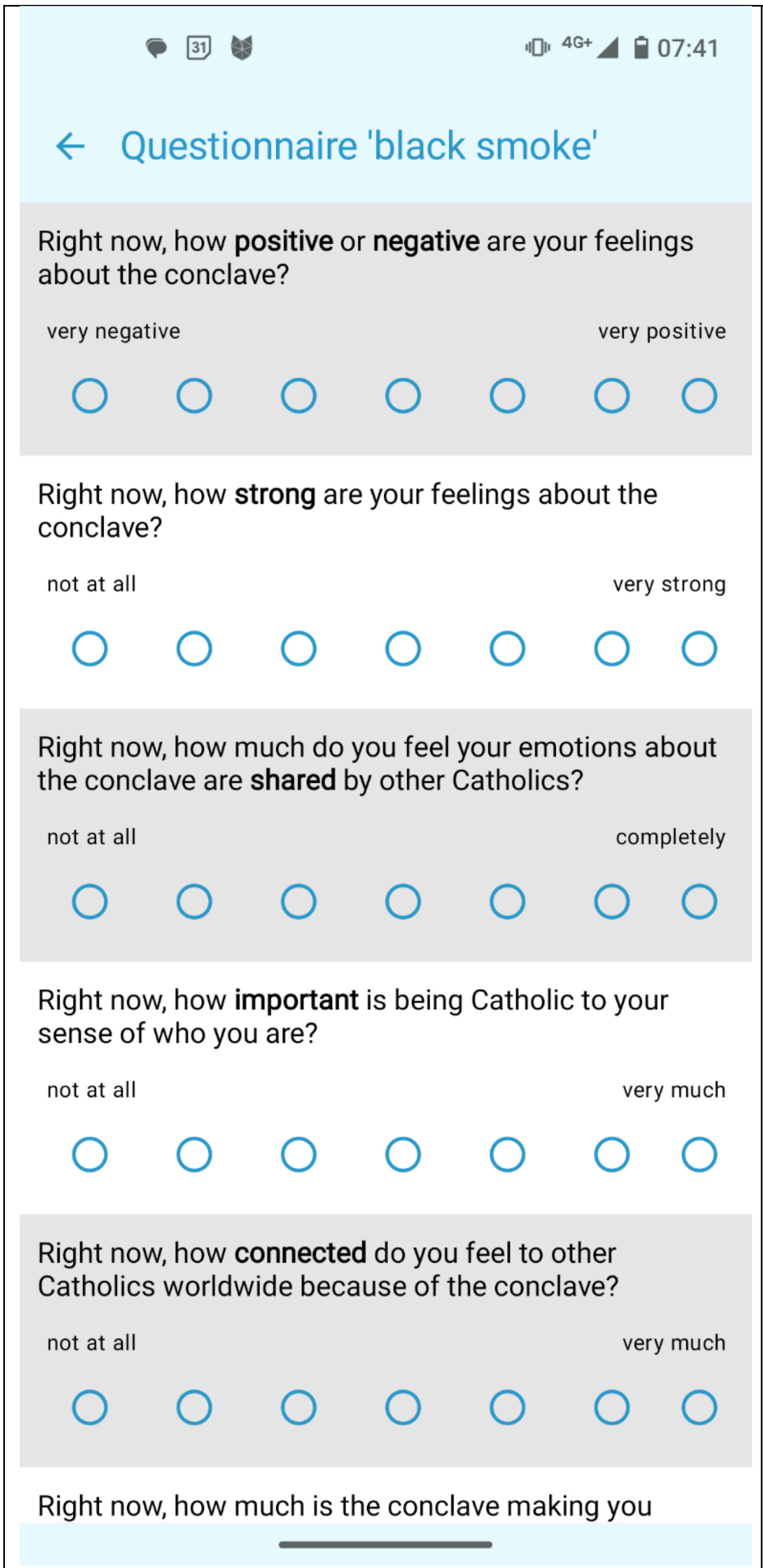
07:41
Questionnaire 'black smoke'
Right now, how **positive** or **negative** are your feelings about the conclave?
very negative
very positive
Right now, how **strong** are your feelings about the conclave?
not at all
very strong
Right now, how much do you feel your emotions about the conclave are **shared** by other Catholics?
not at all
completely
Right now, how **important** is being Catholic to your sense of who you are?
not at all
very much
Right now, how **connected** do you feel to other Catholics worldwide because of the conclave?
not at all
very much
Right now, how much is the conclave making you

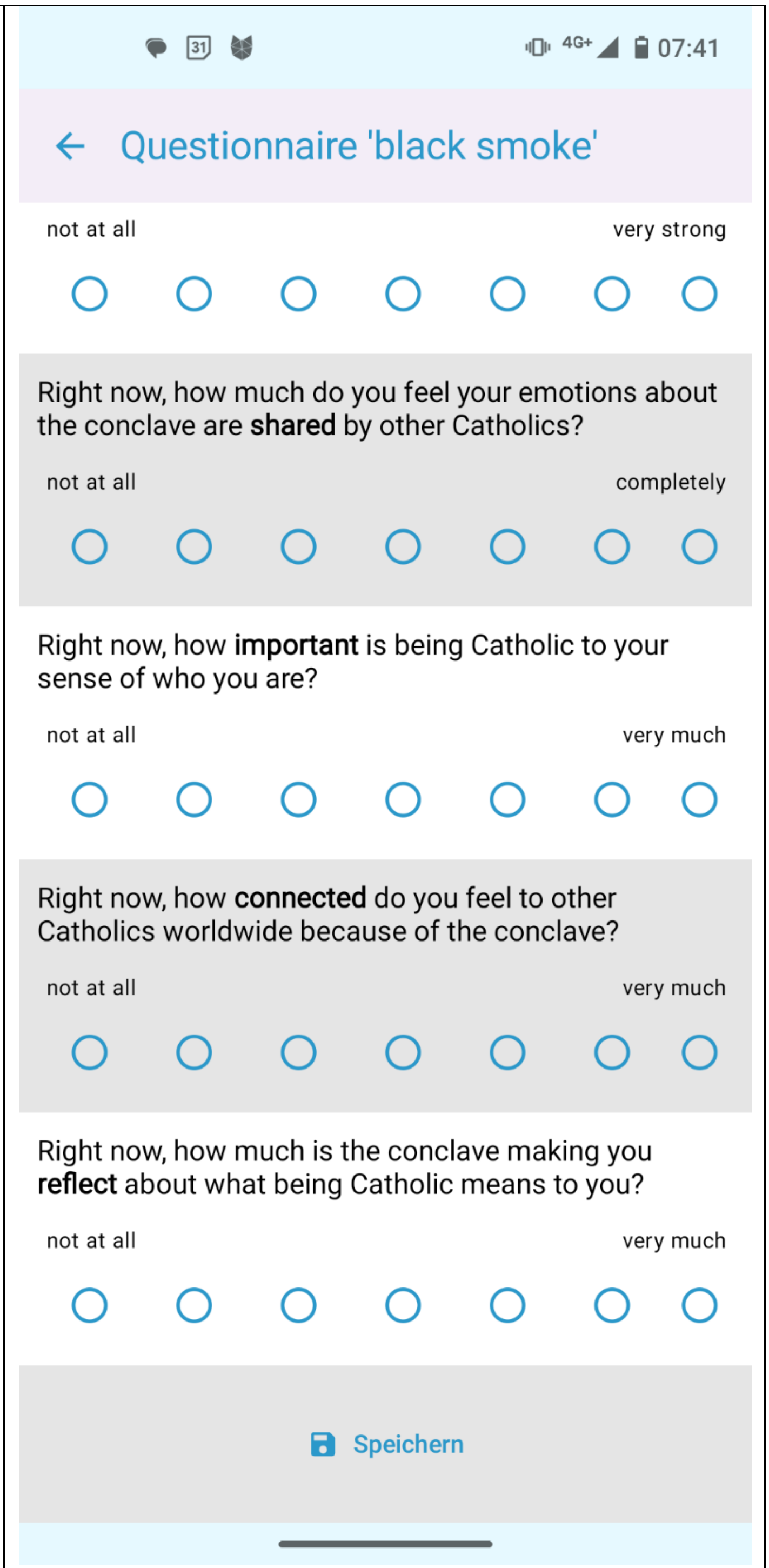
07:41
Questionnaire 'black smoke'
not at all
very strong
Right now, how much do you feel your emotions about the conclave are **shared** by other Catholics?
not at all
completely
Right now, how **important** is being Catholic to your sense of who you are?
not at all
very much
Right now, how **connected** do you feel to other Catholics worldwide because of the conclave?
not at all
very much
Right now, how much is the conclave making you **reflect** about what being Catholic means to you?
not at all
very much
Speichern

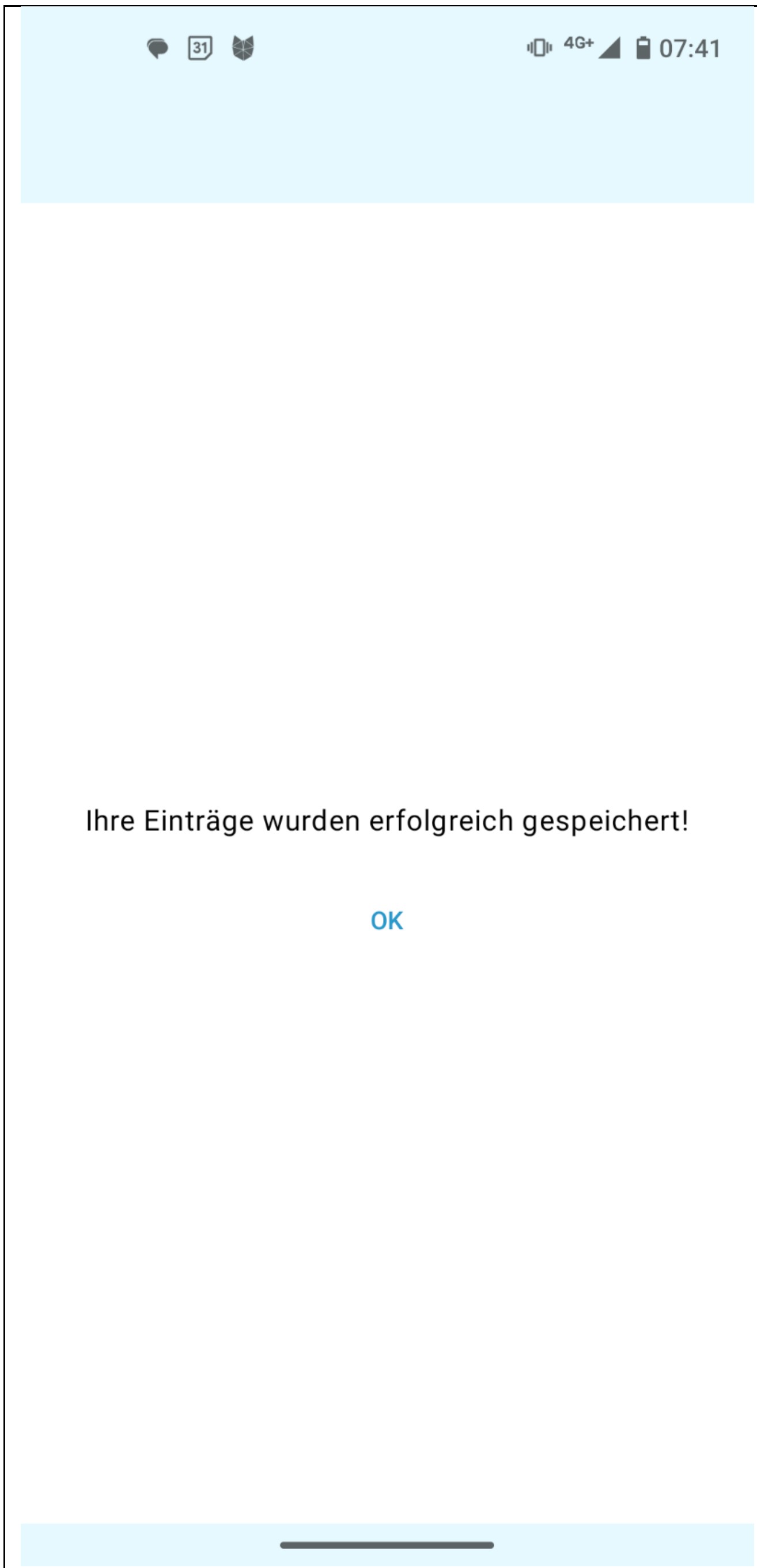
07:41
Ihre Einträge wurden erfolgreich gespeichert!
OK

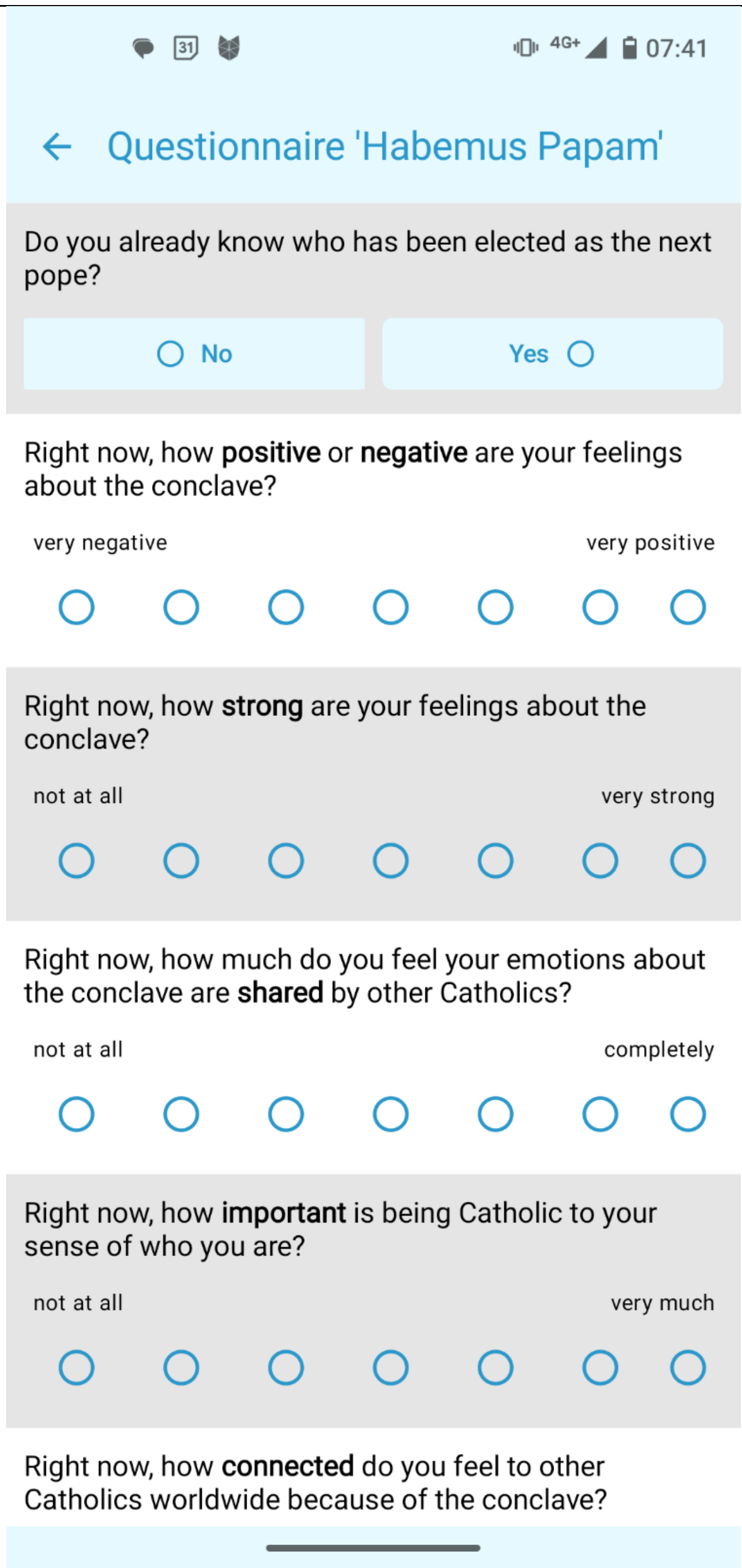
07:41
Questionnaire 'Habemus Papam'
Do you already know who has been elected as the next pope?
No
Yes
Right now, how **positive** or **negative** are your feelings about the conclave?
very negative
very positive
Right now, how **strong** are your feelings about the conclave?
not at all
very strong
Right now, how much do you feel your emotions about the conclave are **shared** by other Catholics?
not at all
completely
Right now, how **important** is being Catholic to your sense of who you are?
not at all
very much
Right now, how **connected** do you feel to other Catholics worldwide because of the conclave?

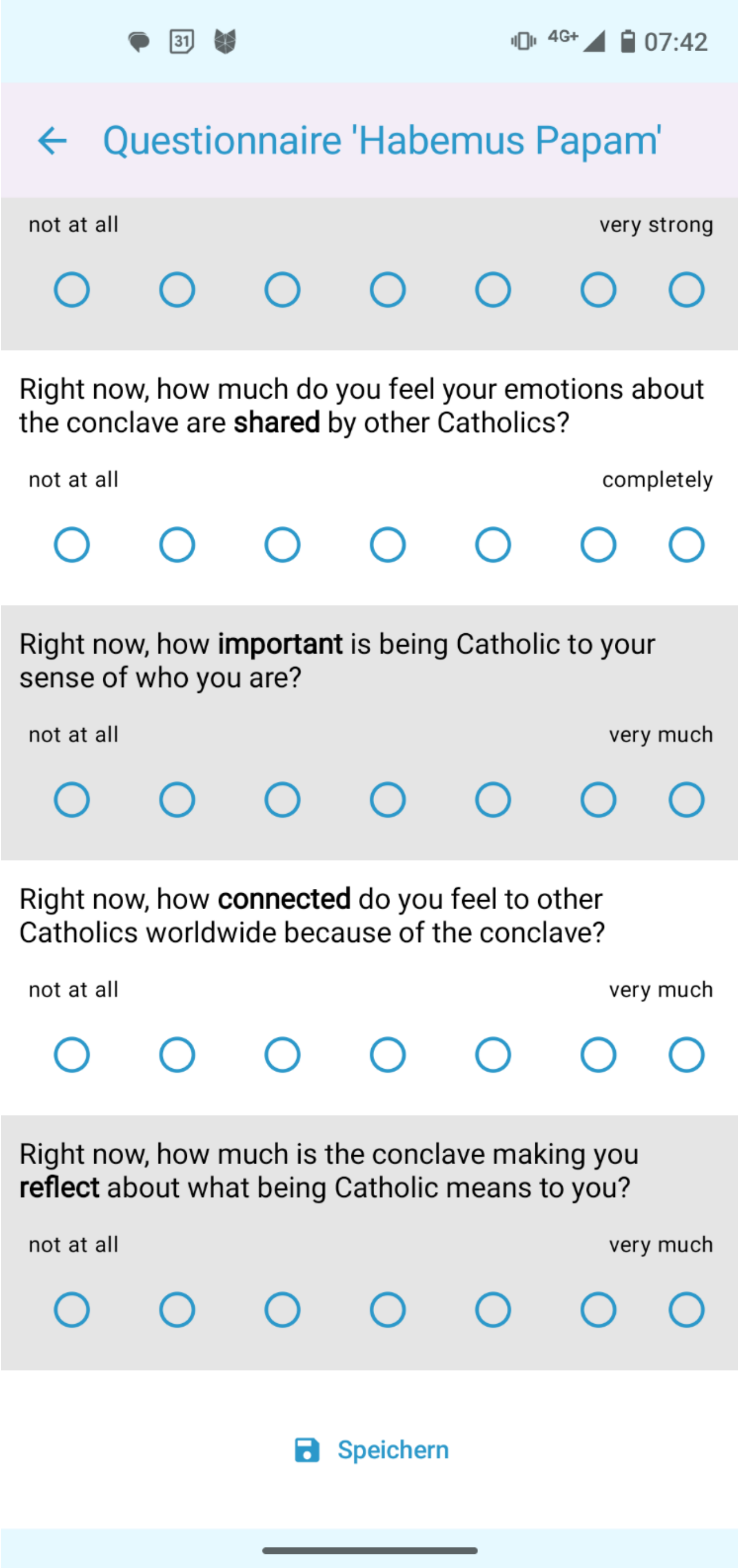

07:42

Questionnaire 'Habemus Papam'

not at all — very strong

Right now, how much do you feel your emotions about the conclave are **shared** by other Catholics?

not at all — completely

Right now, how **important** is being Catholic to your sense of who you are?

not at all — very much

Right now, how **connected** do you feel to other Catholics worldwide because of the conclave?

not at all — very much

Right now, how much is the conclave making you **reflect** about what being Catholic means to you?

not at all — very much

Speichern

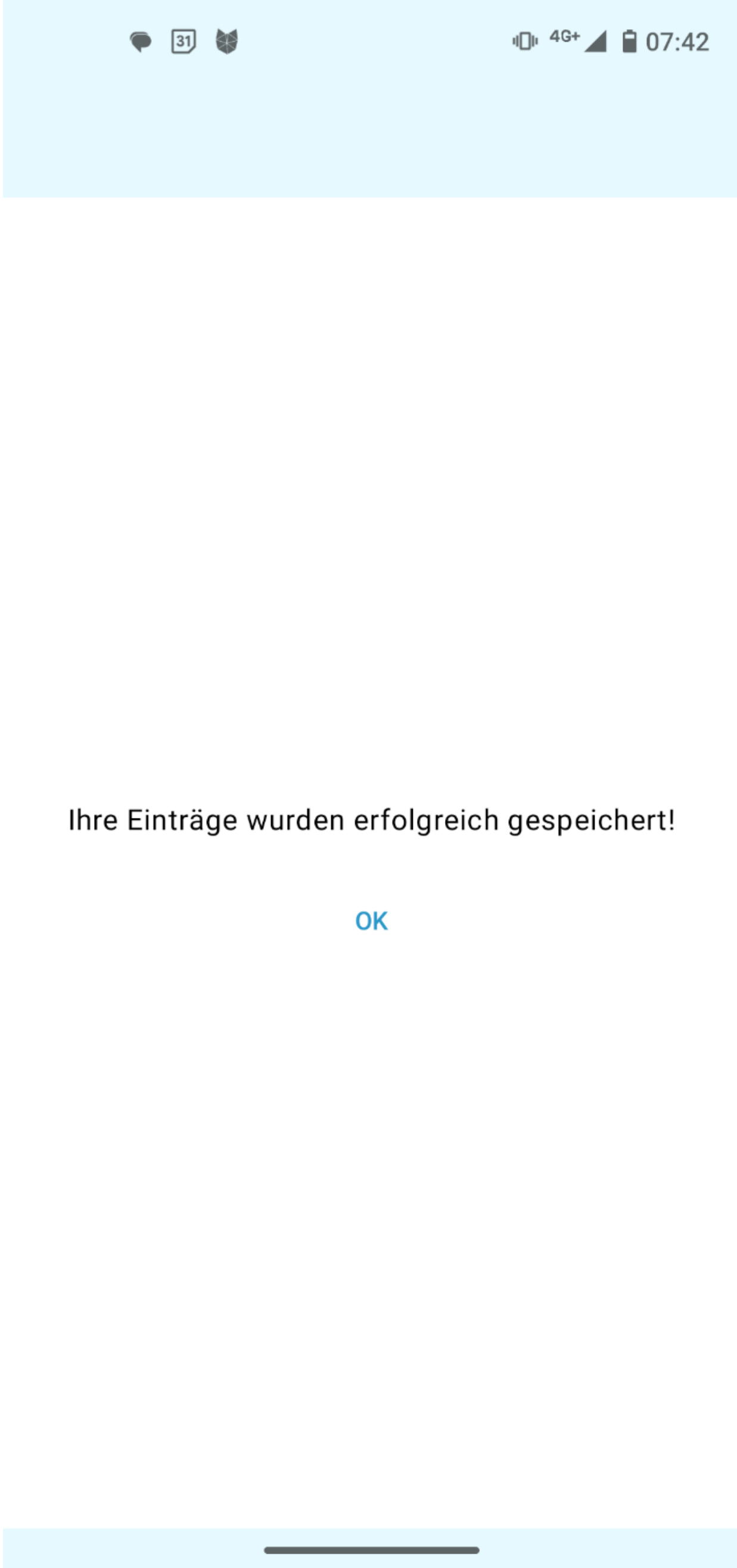

07:42

Ihre Einträge wurden erfolgreich gespeichert!

OK

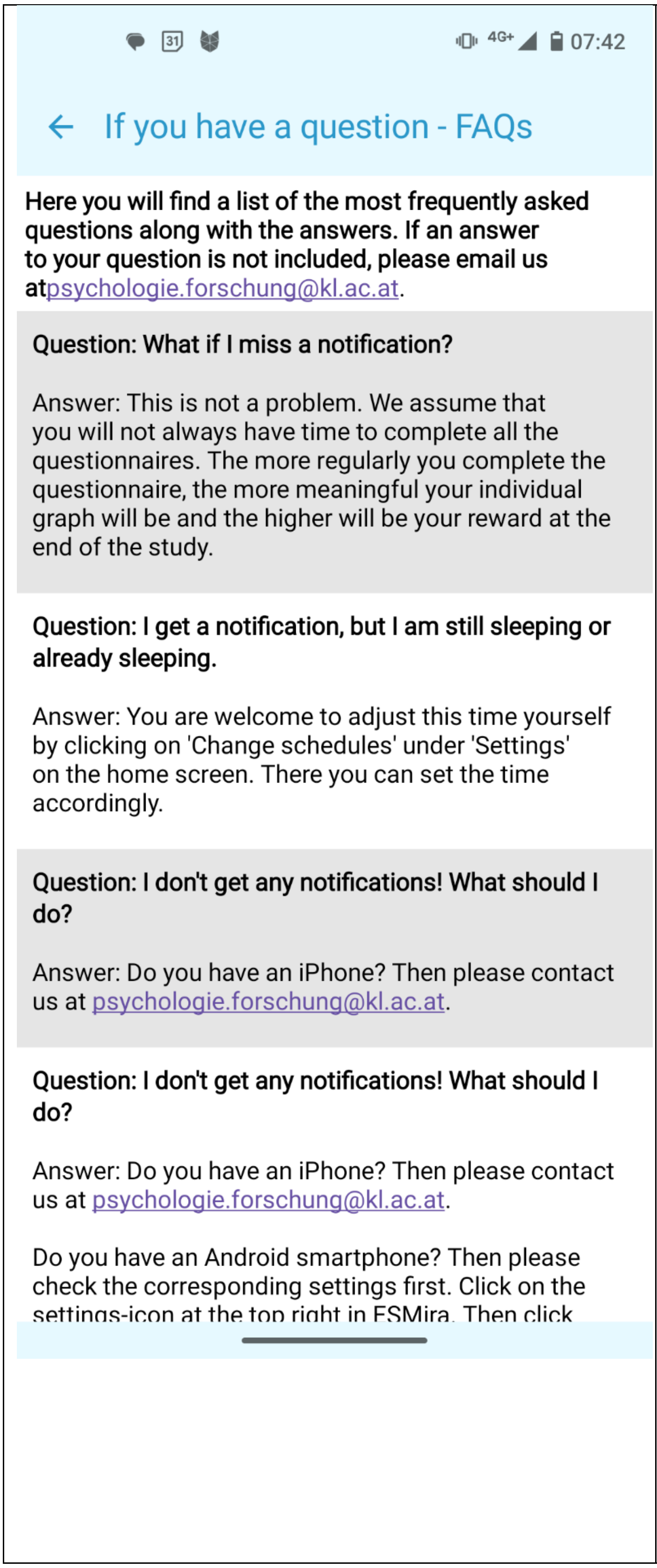
07:42
If you have a question - FAQs
Here you will find a list of the most frequently asked questions along with the answers. If an answer to your question is not included, please email us atpsychologie.forschung@kl.ac.at.
Question: What if I miss a notification?
Answer: This is not a problem. We assume that you will not always have time to complete all the questionnaires. The more regularly you complete the questionnaire, the more meaningful your individual graph will be and the higher will be your reward at the end of the study.
Question: I get a notification, but I am still sleeping or already sleeping.
Answer: You are welcome to adjust this time yourself by clicking on 'Change schedules' under 'Settings' on the home screen. There you can set the time accordingly.
Question: I don't get any notifications! What should I do?
Answer: Do you have an iPhone? Then please contact us at psychologie.forschung@kl.ac.at.
Question: I don't get any notifications! What should I do?
Answer: Do you have an iPhone? Then please contact us at psychologie.forschung@kl.ac.at.
Do you have an Android smartphone? Then please check the corresponding settings first. Click on the

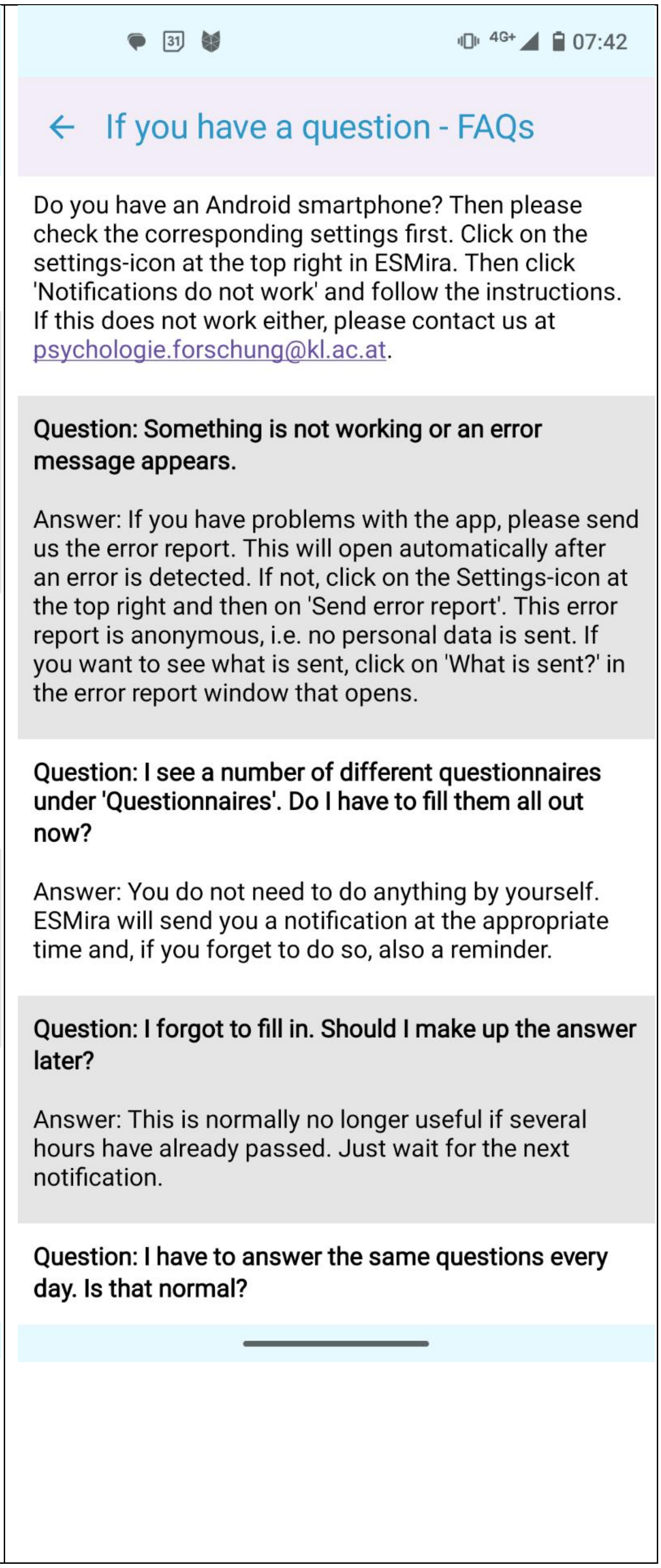
07:42
If you have a question - FAQs
Do you have an Android smartphone? Then please check the corresponding settings first. Click on the settings-icon at the top right in ESMira. Then click 'Notifications do not work' and follow the instructions. If this does not work either, please contact us at psychologie.forschung@kl.ac.at.
Question: Something is not working or an error message appears.
Answer: If you have problems with the app, please send us the error report. This will open automatically after an error is detected. If not, click on the Settings-icon at the top right and then on 'Send error report'. This error report is anonymous, i.e. no personal data is sent. If you want to see what is sent, click on 'What is sent?' in the error report window that opens.
Question: I see a number of different questionnaires under 'Questionnaires'. Do I have to fill them all out now?
Answer: You do not need to do anything by yourself. ESMira will send you a notification at the appropriate time and, if you forget to do so, also a reminder.
Question: I forgot to fill in. Should I make up the answer later?
Answer: This is normally no longer useful if several hours have already passed. Just wait for the next notification.
Question: I have to answer the same questions every day. Is that normal?

07:42

← If you have a question - FAQs

Question: I have to answer the same questions every day. Is that normal?

Answer: Yes, it is intended that way. The purpose of the repetitive questions is to collect a stable mood picture over a longer period of time and to see if it changes over time.

Question: I would like to continue the study on another smartphone. Is that possible?

Answer: In principle, yes. In order for us to fully analyze your data, we ask that you ensure that the same user-ID is used on both smartphones. If you use the same Google account on both Android smartphones, the subject number should be transferred automatically. You can check this by clicking on 'Study information'. At the very top is the 'User ID'. This should be identical for both smartphones. If not, please send us an e-mail at psychologie.forschung@kl.ac.at.

Question: How long does the study actually take?

Answer: 14 days. ESMira will then stop automatically and send further information how to get your payment in Prolific.

Question: I do not want to participate anymore. What should I do?

Answer: In order for you to opt out of the study, click on 'Leave study'. This will end your participation in the study, meaning you will no longer receive notifications. You can still access your graphs - they will not be deleted immediately. If you would like to delete them as well - click on 'Delete data'. The graphics will then

07:43

← If you have a question - FAQs

as well - click on 'Delete data'. The graphics will then be deleted. If you also want to uninstall ESMira, simply uninstall via the Appstore. Please note that if you leave the study early, we will not be able to pay you your incentive.

Question: Where can I find my user-ID and is it anonymous?

Answer: Your user-ID is randomly generated after installing ESMira, i.e. by the app itself, and is completely anonymous. It is only used to be able to link your individual questionnaires with each other. You can find your user-ID under 'Study information' at the top.

Question: What does the menu item 'Update studies' under the settings mean?

Answer: We create the questionnaires centrally on the ESMira server. The ESMira app fetches these questionnaires once after they have been entered in the selected study. In the meantime, however, we may change small things on the questionnaire at the ESMira server, such as correcting typos. Therefore, when you start the ESMira app, it automatically checks whether there is an update. You can also start the study update manually via the menu item 'Update studies'.

Question: What does the menu item 'upload protocol' mean?

Answer: Normally, the data of the completed questionnaires are immediately sent to the ESMira server and saved by clicking on 'Save' in the respective questionnaire. If your smartphone should ever have no internet connection, this is not a problem - the data will first be saved locally and, after a connection

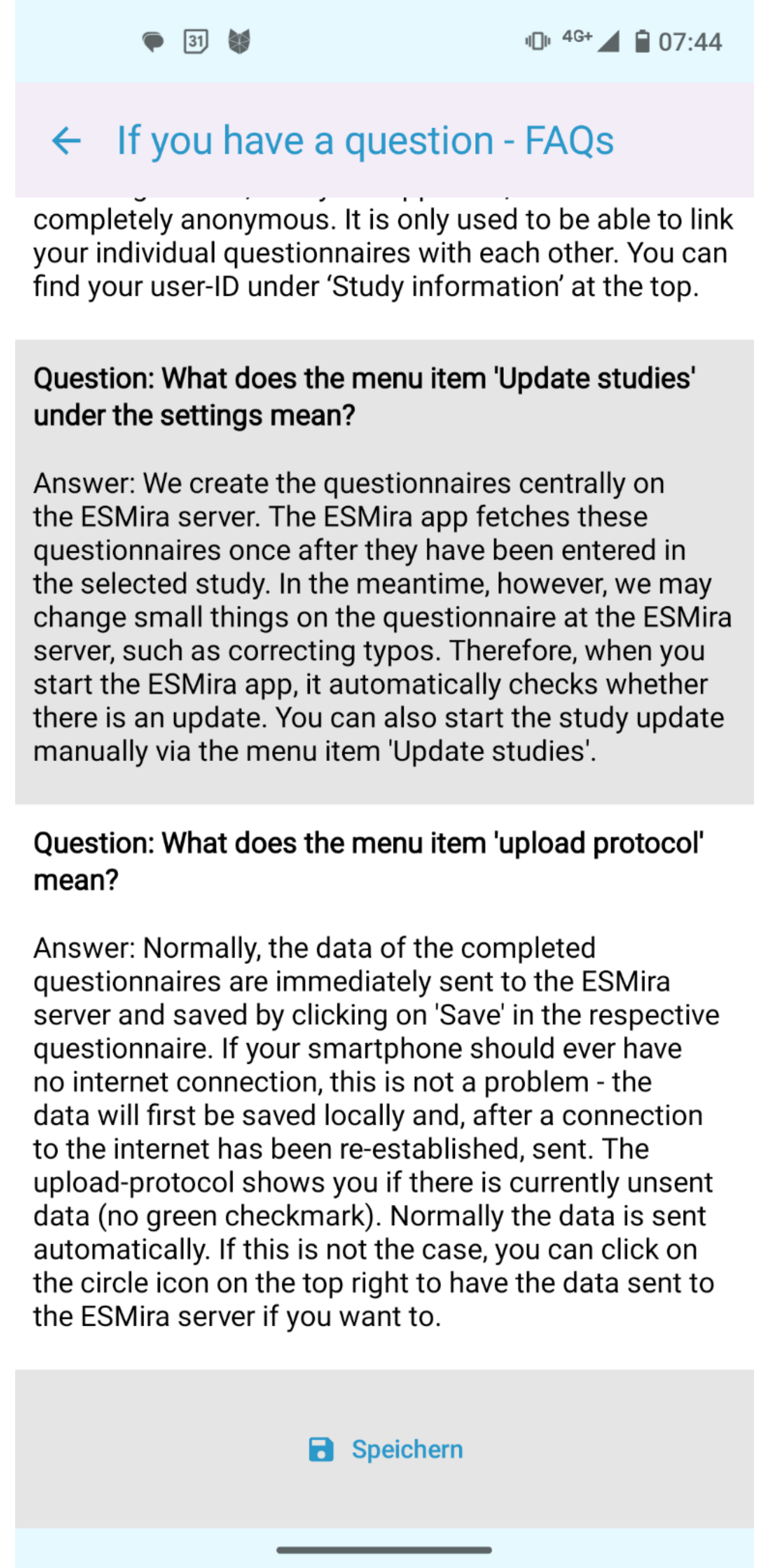
07:44

If you have a question - FAQs

completely anonymous. It is only used to be able to link your individual questionnaires with each other. You can find your user-ID under 'Study information' at the top.

Question: What does the menu item 'Update studies' under the settings mean?

Answer: We create the questionnaires centrally on the ESMira server. The ESMira app fetches these questionnaires once after they have been entered in the selected study. In the meantime, however, we may change small things on the questionnaire at the ESMira server, such as correcting typos. Therefore, when you start the ESMira app, it automatically checks whether there is an update. You can also start the study update manually via the menu item 'Update studies'.

Question: What does the menu item 'upload protocol' mean?

Answer: Normally, the data of the completed questionnaires are immediately sent to the ESMira server and saved by clicking on 'Save' in the respective questionnaire. If your smartphone should ever have no internet connection, this is not a problem - the data will first be saved locally and, after a connection to the internet has been re-established, sent. The upload-protocol shows you if there is currently unsent data (no green checkmark). Normally the data is sent automatically. If this is not the case, you can click on the circle icon on the top right to have the data sent to the ESMira server if you want to.

Speichern

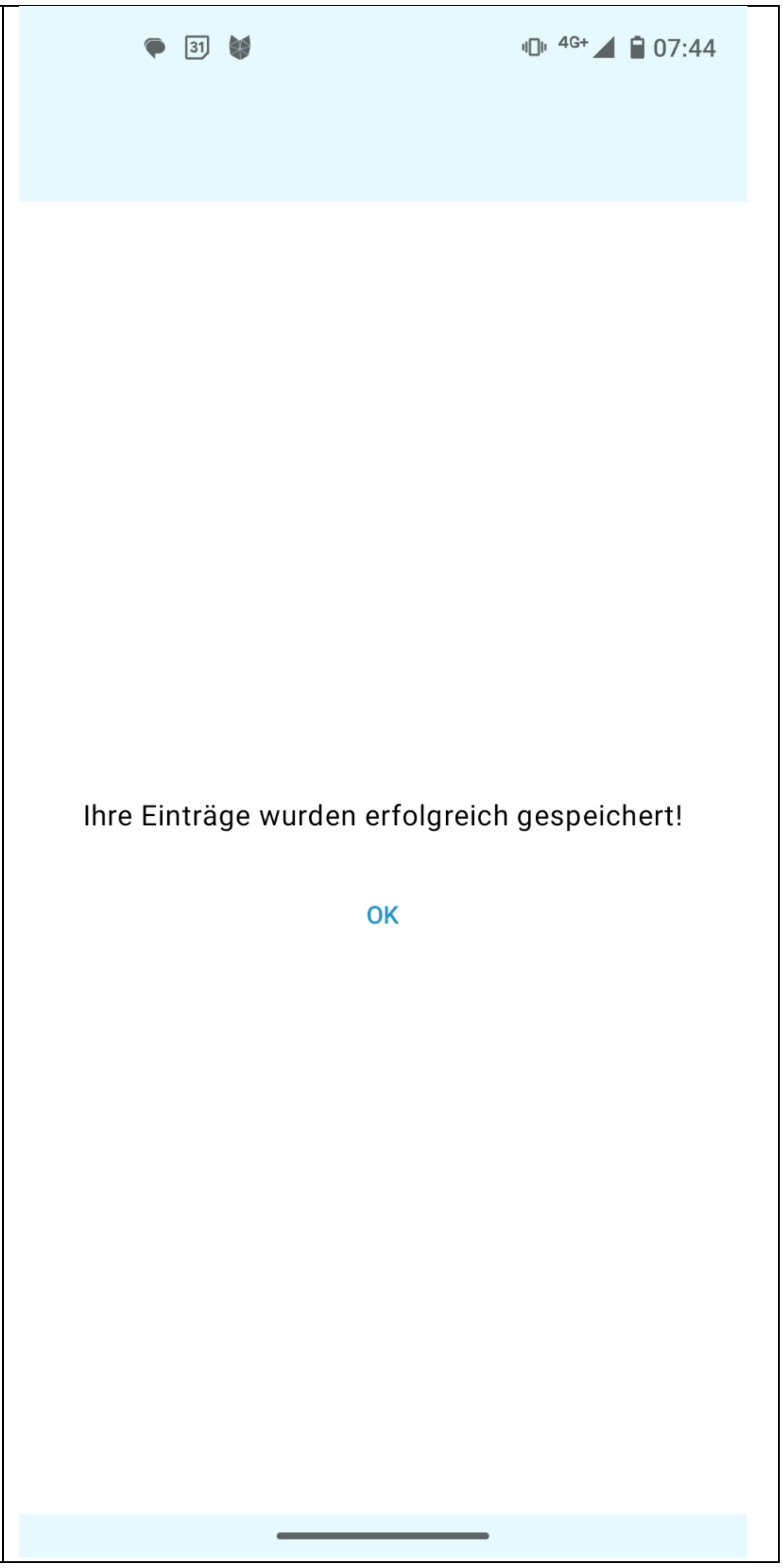
07:44

Ihre Einträge wurden erfolgreich gespeichert!

OK
